# Venous identity requires BMP signalling through ALK3

**DOI:** 10.1038/s41467-019-08315-w

**Published:** 2019-01-28

**Authors:** Alice Neal, Svanhild Nornes, Sophie Payne, Marsha D. Wallace, Martin Fritzsche, Pakavarin Louphrasitthiphol, Robert N. Wilkinson, Kira M. Chouliaras, Ke Liu, Karen Plant, Radhika Sholapurkar, Indrika Ratnayaka, Wiebke Herzog, Gareth Bond, Tim Chico, George Bou-Gharios, Sarah De Val

**Affiliations:** 1 Ludwig Institute for Cancer Research Ltd, Nuffield Department of Medicine, University of Oxford, Oxford, OX3 7DQ UK; 2 Department of Physiology, Anatomy and Genetics, University of Oxford, Oxford, OX1 3PT UK; 3 Department of Infection, Immunity and Cardiovascular Disease and Bateson Centre, University of Sheffield, Sheffield, S10 2TN UK; 4 Institute of Aging and Chronic Disease, University of Liverpool, Liverpool, L7 8TX UK; 5 University of Muenster, Schlossplatz 2, Muenster, 48149 Germany; 6 Cells-in Motion Cluster of Excellence EXC1003-CiM, University of Muenster, Waldeyerstraße 15, Muenster, 48149 Germany

## Abstract

Venous endothelial cells are molecularly and functionally distinct from their arterial counterparts. Although veins are often considered the default endothelial state, genetic manipulations can modulate both acquisition and loss of venous fate, suggesting that venous identity is the result of active transcriptional regulation. However, little is known about this process. Here we show that BMP signalling controls venous identity via the ALK3/BMPR1A receptor and SMAD1/SMAD5. Perturbations to TGF-β and BMP signalling in mice and zebrafish result in aberrant vein formation and loss of expression of the venous-specific gene *Ephb4*, with no effect on arterial identity. Analysis of a venous endothelium-specific enhancer for *Ephb4* shows enriched binding of SMAD1/5 and a requirement for SMAD binding motifs. Further, our results demonstrate that BMP/SMAD-mediated *Ephb4* expression requires the venous-enriched BMP type I receptor ALK3/BMPR1A. Together, our analysis demonstrates a requirement for BMP signalling in the establishment of *Ephb4* expression and the venous vasculature.

## Abstract (web summary)

The establishment of functional vasculatures requires the specification of newly formed vessels into veins and arteries. Here, Neal et al. use a combination of genetic approaches in mice and zebrafish to show that BMP signalling, via ALK3 and SMAD1/5, is required for venous specification during blood vessel development.

## Introduction

Arteriovenous differentiation begins prior to the onset of blood flow, indicating an important role for genetic fate determination^1^. Mammalian arterial–venous fate is acquired in a stepwise manner: arterial identity is established first, while the initial venous structures express both arterial and venous markers prior to embryonic day (E) 9.0, when full venous differentiation occurs concurrent with the expression of *Eph receptor B4* (*Ephb4*)^2^. *Ephb4*, encoding a transmembrane tyrosine kinase, is highly expressed in definitive venous endothelium but is not found in arterial endothelium. Consequently, it is often treated as the definitive venous endothelial identity marker, although some expression is also detected in capillaries and in lymphatic valves^3,4^. Loss of *Ephb4* expression results in embryonic lethality by E10.5, with significant defects in the formation of the cardinal vein while the dorsal aorta is relatively unaffected^5^.

It has been hypothesized that endothelial cells (ECs) are venous by default while arterial identity is acquired; however, growing evidence suggests that venous EC identity is dependent upon dynamic gene regulation. For example, the phosphoinositide-3 kinase-AKT pathway downstream of vascular endothelial growth factor (VEGF-A) actively promotes venous differentiation through inhibition of extracellular signal–regulated kinase/mitogen-activated protein kinase (ERK/MAPK)^6^, whereas the venous-specific orphan nuclear receptor Coup-TFII (*Nr2f2*) actively represses arterial gene expression^7^. However, relatively little is known about the regulatory mechanisms that control venous differentiation: no venous specific enhancers or promoters have been described in the literature, and it is unclear whether venous gene expression is actively stimulated in veins or repressed in arteries.

While the roles of the Notch and VEGF-A signalling pathways in arteriovenous differentiation have been thoroughly investigated, the involvement of other vascular signalling networks in this process is less well understood. In particular, the precise role played by transforming growth factor (TGF)-β signalling in arteriovenous differentiation has been challenging to determine. The TGF-β superfamily of pleiotropic cytokines, including three TGF-β and multiple bone morphogenetic protein (BMP) ligands, are widely found in blood vessels, as are their cognate type II and type I transmembrane receptor kinases^8^. Ligand binding results in the intracellular phosphorylation of the receptor-regulated SMADs (R-SMADs), which form heteromeric complexes with the common-mediator SMAD4 and translocate to the nucleus where they directly bind DNA^9^.

Mutations in the BMP receptor *ALK1(ACVRL1)*, *SMAD4* and the accessory type III receptor *ENG* are associated with the human condition Hereditary Hemorrhagic Telangiectasia (HHT), characterized by arteriovenous malformations and mucocutaneous telangiectasias^10^. However, although gene ablation studies in mice support a crucial role for TGF-β and BMP signalling in the vasculature^11–16^, the use of different Cre lines, confounding effects of cardiac valve defects and inconsistent analysis of arteriovenous differentiation in these mutants has made conclusive analysis of the role of these pathways in early arterial and venous identity challenging. Furthermore, while studies in zebrafish demonstrate a role for BMP signalling through the receptor BMPR2 in venous-specific angiogenic sprouting^1,17,18^, the requirement for BMP signalling in dorsal–ventral axis specification prior to vascular specification has thus far prevented analysis at stages relevant to arterial or venous identity.

In this paper, we investigate arteriovenous differentiation after EC-specific deletion of SMAD4 in both mice and fish, demonstrating a requirement for SMAD4 in the acquisition of venous but not arterial identity. Further, we conduct a comprehensive analysis of the transcriptional regulation of the essential venous identity gene *Ephb4*, identifying a venous endothelial-specific enhancer containing essential SMAD-binding motifs and binding SMAD1/5 in ECs. Analysis of SMAD1/5 chromatin immunoprecipitation–sequencing (ChIP-seq) data finds similar SMAD1/5-binding peaks around other venous-associated genes, including *Coup-TFII/Nr2f2*. Lastly, we demonstrate that the BMP type I receptor ALK3/BMPR1A is specifically expressed in early venous ECs and is required for venous identity.

## Results

### Absence of endothelial Smad4 results in the loss of Ephb4

**Fig. 1:**
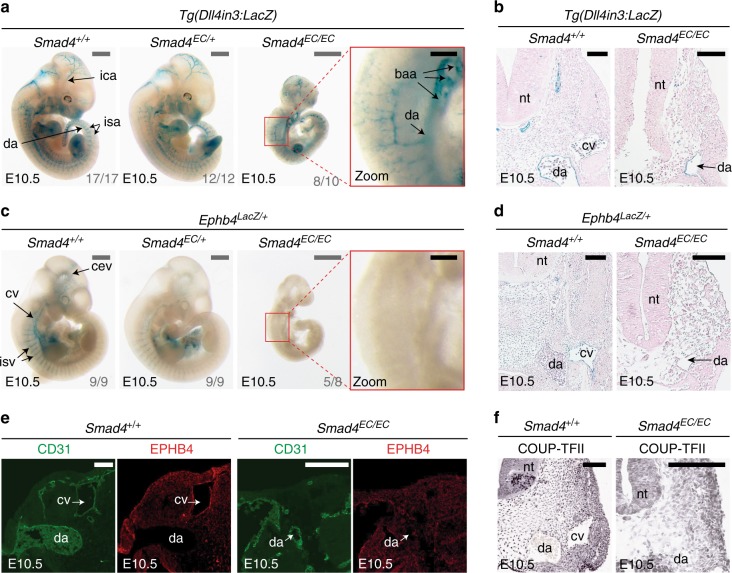
Endothelial-specific knockout of *Smad4* does not affect arterial identity but results in the loss of *Ephb4* expression. **a**, **b** Representative E10.5 whole-mount images (**a**) and transverse sections (**b**) from wild-type *Smad4*^*+/+*^ (*n* = 17), heterozygous *Smad4*^*EC/+;*^ (*n* = 12) and homozygous *Smad4*^*EC/EC*^ (*n* = 10) embryos all expressing the arterial Dll4in3:*LacZ* transgene (five litters in total). Robust transgene expression, specific to arterial endothelial cells, was seen in all embryos regardless of *Smad4* genotype. Grey scale bars are 500 μm, black scale bars are 100 μm. **c**, **d** Representative E10.5 whole-mount images (**c**) and transverse sections (**d**) from wild-type *Smad4*^*+/+*^ (*n* = 9), heterozygous *Smad4*^*EC/+;*^ (*n* = 9) and homozygous *Smad4*^*EC/EC*^ (*n* = 8) embryos also transgenic for the venous marker *Ephb4*^*LacZ*^ (four litters total). Robust X-gal activity is detected in the veins of *Smad4*^*+/+*^ embryos but is reduced in *Smad4*^*EC/+*^ embryos and absent in *Smad4*^*EC/EC*^. Red box denotes zoomed region, grey numbers on bottom right denote the number of embryos similar to picture shown. Grey scale bars are 500 μm, black scale bars are 100 μm. Outliers are shown in Supplementary Figure 1e. **e**, **f** Expression of the venous endothelial cell markers EPHB4 (**e**) and COUP-TFII (**f**) in transverse sections from E10.5 *Smad4*^*+/+*^ and *Smad4*^*EC/EC*^ embryos. In addition to venous endothelial cells, COUP-TFII is expressed by arterial smooth muscle cells and other mesenchymal cells (as reported by You et al.^7^). White scale bars are 100 μm. ^EC^ indicates Tie2:Cre-mediated deletion, ^+/+^ indicates Cre−, ^EC/+^ indicates Cre+,Smad4^fl/+^ and ^EC/EC^ indicates Cre+;Smad4^fl/fl^. da = dorsal aorta, ica = internal carotid artery, isa = intersomitic arteries, isv = intersomitic vessel, baa = branchial arch arteries, nt = neural tube, cv = cardinal vein, cev = branches of cerebral venous plexus. See also Supplementary Figure 1

To investigate the role of the canonical TGF-β and BMP pathways in arteriovenous identity, we re-examined the consequences of loss of the common mediator protein SMAD4 in ECs. As reported by Lan et al.^14^, mouse embryos with endothelial-specific deletion of *Smad4* (*Tie2:Cre;Smad4*^*fl/fl*^, referred to here as *Smad4*^*EC/EC*^) die between E9.5 and E10.5 and exhibit growth retardation and gross vascular defects. Analyses of wild-type (WT) and mutant mouse embryos at E10.5 suggest that early arterial identity is unperturbed after *Smad4* deletion: the dorsal aorta could be clearly detected by morphological analysis, arterial markers DLL4 and NRP1 were detected in all *Smad4*^*EC/EC*^ embryos and expression of the arterial Dll4in3:*LacZ* enhancer transgene^19–21^ was clearly detected in the apparent dorsal aorta in even severely growth retarded *Smad4*^*EC/EC*^*; Dll4in3:LacZ* embryos (Fig. 1a, b and Supplementary Fig. 1a–d).

To analyse venous formation in the absence of SMAD4, knock-in *Ephb4*^*LacZ/+*^ mice^5^ (which express *LacZ* specifically in *Ephb4*+ venous ECs) were crossed into the *Smad4*^*EC/EC*^ line. Strikingly, very little *Ephb4*^*LacZ*^ expression was detected in *Smad4*^*EC/EC*^ embryos by E10.5 (Fig. 1c, d and Supplementary Fig. 1e). Transverse sections through E10.5 *Smad4*^*EC/EC*^*;Ephb4*^*LacZ/+*^ embryos confirmed the lack of *Ephb4*^*LacZ*^ expression and revealed a morphological absence of a discernible cardinal vein (Fig. 1d), a phenotype shared with *Ephb4* null embryos^5^. Loss of differentiated venous endothelium was further confirmed by immunohistochemical analysis of endogenous EPHB4 and COUP-TFII (a venous-specific orphan nuclear receptor) expression relative to the pan-endothelial CD31 marker in *Smad4*^*+/+*^ and *Smad4*^*EC/EC*^ embryos (Fig. 1e, f). The requirement to use the endothelial-specific Tie2:Cre driver (which is not consistently expressed in early embryo littermates, Supplementary Fig. 1f) makes it challenging to establish from these results whether SMAD4 is required for initiation or maintenance of *Ephb4* expression: some venous-positioned vessels were detected in some E9.5 *Smad4*^*EC/EC*^ embryos (Supplementary Fig. 1g), potentially reflecting *Smad4* expression in early vessels prior to Tie2:Cre activity or indicating that SMAD4 is not required for initial *Ephb4* expression. However, this result clearly demonstrates a requirement for SMAD4 in *Ephb4* expression as the early veins develop and differentiate and for the proper formation of the venous vasculature.

**Fig. 2:**
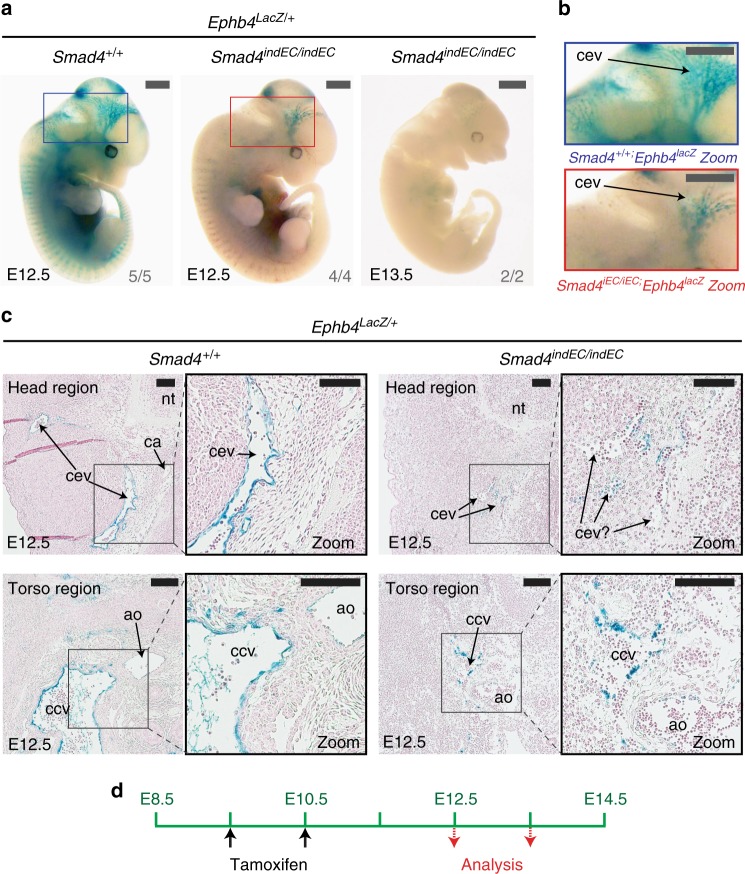
Induced endothelial-specific deletion of *Smad4* after E9.5 results in reduced *Ephb4* expression and dysfunctional venous structures. **a**–**c** Representative whole-mount images (**a**), zoomed head region images (**b**) and transverse sections through head and torso regions (**c**) from *Smad4*^*+/+*^ *and Smad4*^*indEC/indEC*^ embryos also transgenic for the venous marker *Ephb4*^*LacZ*^. Embryos investigated three and four days after tamoxifen induction at E9.5 and E10.5; *Smad4*^*+/+*^ control for E13.5 embryos is shown in Supplementary Fig 2b. *Ephb4*^*LacZ*^ expression was greatly decreased in *Smad4*^*indEC/indEC*^ embryos compared to wild-type (WT) control at E12.5 (WT *n* = 5, null *n* = 4 from two litters) and near-ablated in *Smad4*^*indEC/indEC*^ embryos by E13.5 (*n* = 2 from one litter). Grey scale bars are all 1000 μm, black scale bars are all 100 μm. **d** Schematic detailing tamoxifen regime and timing. Blue and red boxes denote zoomed region, numbers on bottom right-hand corner of each whole-mount image denote the number of embryos similar to picture shown. ^indEC^ indicates CDH5(PAC):Cre/ERT2-mediated deletion, ^+/+^ indicates Cre−. cev = branches of cerebral venous plexus, ca = cerebral artery, ccv = common cardinal vein, ao = aortal, nt = neural tube. See also Supplementary Figure 2

While these results strongly support a role for the TGF-β or BMP pathways in venous identity, these pathways have also been implicated in other endothelial functions, including angiogenic sprouting, smooth muscle recruitment, vascular stability and proliferation^14,15,18,22^. Therefore, it is possible that the venous phenotype seen in *Smad4*^*EC/EC*^ embryos is secondary to a more general vascular defect that more severely affects veins comparative to the earlier fated arteries. However, *Ephb4*^*LacZ*^ expression was also consistently reduced in the heterozygous *Smad4*^*EC/+*^*;Ephb4*^*LacZ*^ embryos (Fig. 1c), which were normal sized and exhibited no embryonic lethality. Similar sized (but younger) *Smad4*^*+/+*^*;Ephb4*^*LacZ/+*^ embryos also displayed clear venous *LacZ* expression (Supplementary Fig. 2a), suggesting growth retardation was unlikely to be the principle driver of reduced *Ephb4*^*LacZ*^. Additionally, induced endothelial-specific deletion of *Smad4* (referred to here as *indEC*) after the initial vasculature was formed (using induction of CDH5(PAC):CreERT2^23^ at either E9.5 or E11.5) also resulted in significant loss of *Ephb4*^*LacZ*^ activity and eventual embryonic death (Fig. 2 and Supplementary Fig. 2b–c). While some venous structures could be detected in these mice three days after Cre induction, they exhibited defective morphology and fewer *Ephb4*^*lacz*^+ cells (Fig. 2c).

**Fig. 3:**
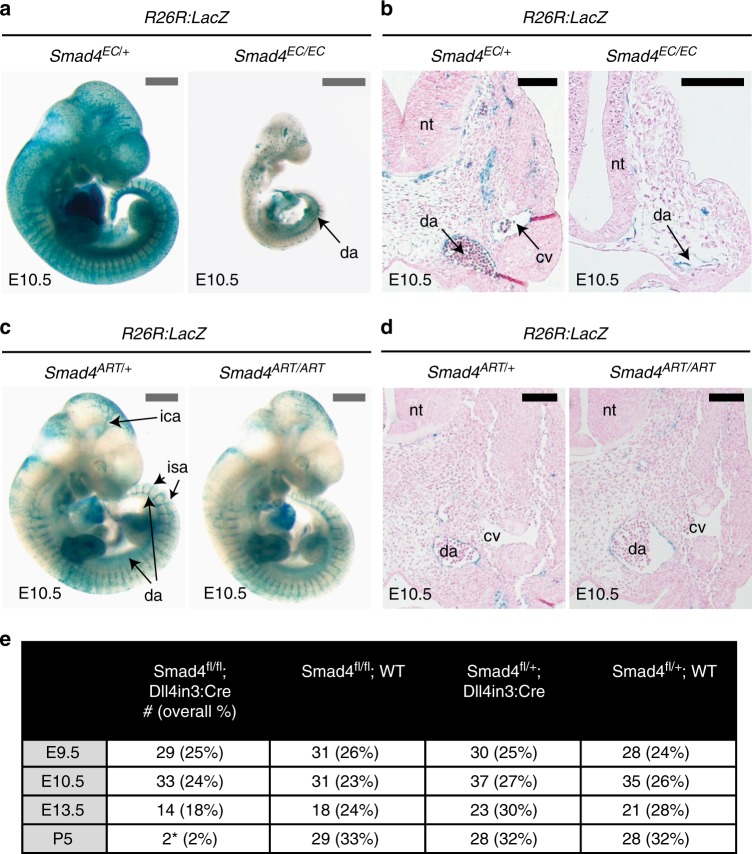
Deletion of *Smad4* specifically in arterial endothelial cells does not affect vascular patterning or early embryonic development. **a**, **b** Representative pan-endothelial *Tie2:Cre;Smad4*^*fl/+*^ and *Tie2:Cre;Smad4*^*fl/fl*^ whole-mount images (**a**) and transverse sections (**b**) from E10.5 embryos also transgenic for the Cre-reporter *Rosa26R:LacZ*. The cardinal vein (cv) cannot be identified in the *Smad4*^*fl/fl*^ embryo, although the dorsal aorta (da) can be seen in both. Grey scale bars are 500 μm, black scale bars are 100 μm. **c**, **d** Representative arterial-specific *Dll4in3:Cre;Smad4*^*fl/+*^ and *Dll4in3:Cre;Smad4*^*fl/fl*^ whole-mount images (**c**) and transverse sections (**d**) from E10.5 embryos also transgenic for the Cre-reporter *Rosa26R:LacZ*. Arterial-specific deletion of *Smad4* had no effect on vasculature development at E10.5. Grey scale bars are 500 μm, black scale bars are 100 μm. **e** Observed frequency of *Smad4*^*fl/fl*^*;Dll4in3:Cre* embryos from E9.5 to P5. Only two sick (*) P5 *Smad4*^*fl/fl*^*;Dll4in3:Cre* animals were recovered. ^EC^ indicates Tie2:Cre-mediated deletion, ^ART^ indicates Dll4in3:Cre-mediated deletion, da = dorsal aorta, nt = neural tube, cv = cardinal vein, ica = inner cerebral artery, isa = intersomitic arteries. See also Supplementary Figure 3

To further rule out the possibility that either a general vascular or arterial defect underlies the lack of venous identity in *Smad4*^*EC/EC*^, we examined the consequences of deleting *Smad4* specifically in a subset of ECs using a Dll4in3 enhancer-driven Cre transgenic mouse (Dll4in3:Cre). Like endogenous *Dll4*, the Dll4in3 enhancer is active in arterial but not in venous ECs and at the angiogenic front^19,21^. Consequently, Dll4in3:Cre is specifically active in these cell types (Supplementary Fig. 3a). This transgene is also active within the endocardium and valves of the heart in similar patterns to Tie2:Cre (Supplementary Fig. 3b–c). While analysis of the activity of the Cre reporter *Rosa26R:LacZ* (*R26R:LacZ*) in E10.5 *Tie2:Cre; Smad4*^*fl/fl*^ embryos recapitulated the venous defects reported in Figs. 1 and 2 (Fig. 3a, b), analysis of *R26R:LacZ* activity in *Dll4in3:Cre;Smad4*^*fl/fl*^ embryos (referred to here as *Smad4*^*ART/ART*^) found no vascular defects (Fig. 3c, d). Further, the observed frequency of *Smad4*^*ART/ART*^ embryos corresponded to expected Mendelian ratios until E13.5 (Fig. 3e). This further indicates that the early venous defects seen in *Smad4*^*EC/EC*^ embryos are not downstream of a general vascular phenotype or from arterial-specific defects and that SMAD4 is not required for arterial identity and differentiation in the early embryo.

### A venous endothelium-specific enhancer for the *Ephb4* gene

**Fig. 4:**
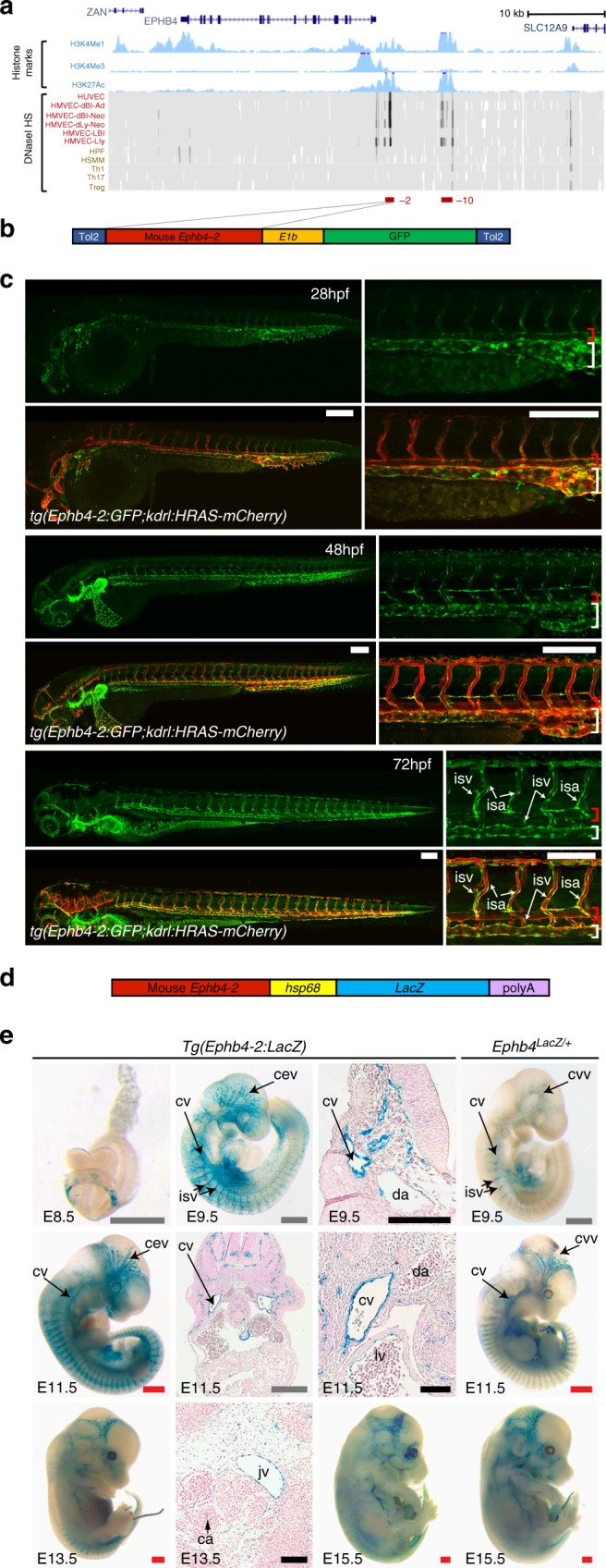
The vein-specific Ephb4-2 enhancer recapitulates endogenous *Ephb4* expression in fish and mouse. **a** Region around the human *EPHB4* gene as seen on UCSC Browser (http://genome.ucsc.edu). Within histone marks, the three tracks show the enhancer-associated H3K4Me1 and H3K27Ac and promoter-associated H3K4Me3 marks found in human umbilical vein endothelial cells as light blue peaks. Within DNaseI hypersensitivity, the heat maps show DNaseI hypersensitive (HS) regions found in different cell lines, with endothelial cells labelled in red and non-endothelial cells labelled in orange. The endothelial histone marks and endothelial-specific DNase I hypersensitivity indicate two potential endothelial enhancer regions (named -2 and -10, marked as red horizontal lines). **b**, **c** The Tol2 Ephb4-2:*E1b*:GFP transgene (**b**) directs venous expression of the green fluorescent protein (GFP) reporter gene in a transgenic zebrafish line also expressing the pan-endothelial kdrl:HRAS-mCherry (**c**). No enhancer activity (as detected by GFP expression) was seen in the dorsal aorta, whereas robust activity was detected in the cardinal and ventral veins. Expression is detected in all intersegmental vessels. White scale bars are 100 μm. Red bracket = dorsal artery; white bracket = axial veins; isv = intersegmental vein. **d**, **e** The Ephb4-2:*hsp68:LacZ* transgene (**d**) directs vein-specific expression of the *LacZ* reporter gene in transgenic mice (**e**) as compared to *Ephb4*^*LacZ/+*^. Black scale bars are 100 μm, grey scale bars are 500 μm, red scale bars are 1000 μm. isv = intersomitic vessel, cv = cardinal vein, da = dorsal aorta, jv = jugular vein, ca = carotid artery, ccv = common cardinal vein, cev = branches of the cerebral venous plexus, lv = left ventricle. See also Supplementary Figure 4

Our results clearly demonstrate that vascular ablation of SMAD4 results in the loss of *Ephb4* expression and venous development. This suggests a crucial role for TGF-β or BMP signalling in *Ephb4* expression and consequently in venous identity. However, analysis of *Smad4* mutant embryos cannot differentiate between direct and indirect targets of the TGF-β and BMP pathways. Therefore, to investigate whether SMAD4 (in combination with R-SMADs) was involved in *Ephb4* expression, we investigated the transcriptional regulation of *Ephb4* in the early vasculature. Complex spatial and temporal patterns of gene expression often involve the use of one or more gene enhancers^24^, and most endothelial-specific genes are known to be primarily regulated by distal enhancer elements^25^. Enhancers are *cis*-acting DNA sequences enriched for transcription factor-binding sites and are associated with regions of open chromatin with specific histone marks (e.g. H3K4Me1 and H3K27Ac)^26^. Analysis of the *Ephb4* locus for sequences rich in enhancer-associated histone modifications and DNaseI hypersensitivity sites^26–28^ identified only two regions containing these marks specifically in ECs (Fig. 4a and Supplementary Fig. 4a). We termed these putative regulatory elements the Ephb4-2 and Ephb4-10 enhancers, reflecting their distance in kb from the transcriptional start site (TSS). Sequence analysis after ClustalW alignment of the human and mouse sequences of Ephb4-2 and Ephb4-10 showed that both enhancers contained a number of conserved ETS-binding elements (EBE), which are known to be essential for endothelial enhancer activity^25^ (Supplementary Fig. 4b–d). ETS transcription factor binding at these two sites was also indicated by analysis of a human genome-wide map of the Ets family member ERG binding in human umbilical vein endothelial cells (HUVECs) generated by Fish et al.^29^ (Supplementary Figure 4e).

To establish whether the putative Ephb4-2 and Ephb4-10 enhancers functioned in vivo, we first cloned the mouse sequences upstream of the E1b minimal promoter^30^ and the green fluorescent protein (GFP) reporter gene (Fig. 4b) and examined their activity in transgenic zebrafish. The Ephb4-10 putative enhancer did not drive GFP expression in ECs (Supplementary Fig. 4f). The Ephb4-2 putative enhancer was able to direct robust GFP expression in transgenic fish specifically in the axial veins but not in the axial arteries from early venous specification through later stages of differentiation (Fig. 4c and Supplementary Fig. 4f–j). To determine whether this enhancer was also active in mice, we next cloned the same murine Ephb4-2 enhancer upstream of the *hsp68* minimal promoter and *LacZ* reporter gene and generated transgenic mice. As expected, the Ephb4-2 enhancer was able to drive reporter gene activity specifically to venous ECs during embryonic development (Fig. 4d, e). *Tg(Ephb4-2:LacZ)* mouse embryos showed patterns of *LacZ* expression strikingly similar to those seen in *Ephb4*^*LacZ/+*^ embryos (Fig. 4e), although the intensity of expression was greater in the enhancer line, reflecting the multiple copies of the Ephb4-2:*LacZ* transgene comparative to the single copy in *Ephb4*^*LacZ*/+^ knock-in. Of note, Ephb4-2:*LacZ* expression exactly correlated with the specific expression of endogenous *Ephb4* in the venous intersomitic vessels. This contrasts with the activity of Ephb4-2:GFP in zebrafish, where expression was seen in the orthologous intersegmental arteries and veins (Fig. 4). This discrepancy may be due to differences in the pathways driving the formation of these vessels between mammals and fish (as the Ephb4-2:GFP transgene contains the mouse sequence) or reflect the requirement for flow in the specification of these vessels in zebrafish or relate to the high levels of proliferation seen in these zebrafish vessels^31^. In conclusion, this enhancer analysis clearly demonstrates that the mouse Ephb4-2 sequence represents a venous-specific enhancer for *Ephb4* that closely mimics the expression of the endogenous gene in mice, providing us with a powerful tool to elucidate the regulators of *Ephb4* in the venous vasculature.

### The Ephb4-2 enhancer requires SMAD-binding motifs

**Fig. 5:**
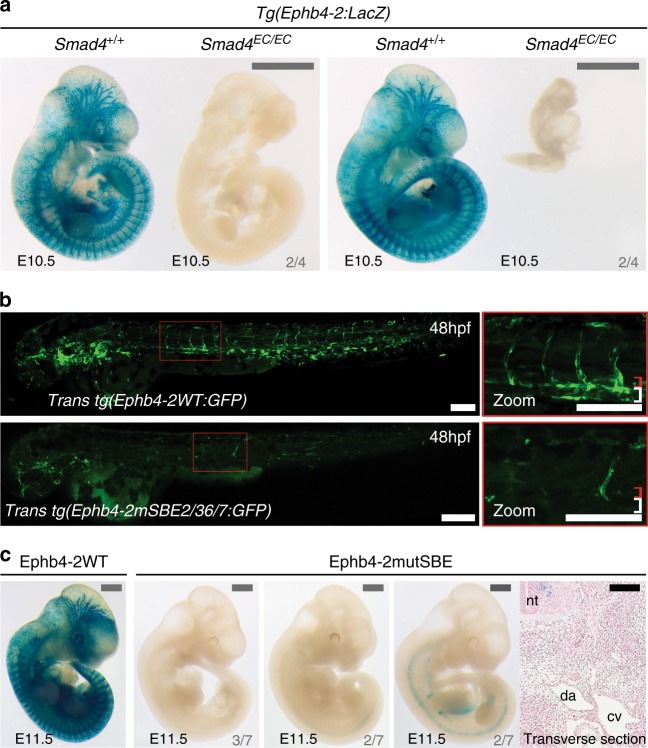
Activity of the Ephb4-2 enhancer requires SMAD4. **a** Two sets of E10.5 *Smad4*^*+/+*;^*Ephb4-2:LacZ* and *Smad4*^*EC/EC*^;*Ephb4-2:LacZ* littermates demonstrates loss of Ephb4-2 enhancer activity after loss of SMAD4 regardless of extent of growth retardation (*n* = 4 from 4 litters). Although some embryos exhibited severe growth retardation, similar sized younger embryos had robust Ephb4-2:*LacZ* expression (see Supplementary Fig. 5). Grey scale bars are 1000 μm. **b**, **c** Mutation of two composite SMAD-binding elements (SBE2/3 and SBE6/7) within the Ephb4-2 enhancer resulted in near-total loss of enhancer activity compared to wild-type enhancer in F0 transgenic zebrafish (**b**, mosaic due to nature of Tol2-mediated F0 transgenesis) and mice (**c**). Transverse section taken through the strongest *lacZ*-expressing F0 Ephb4-2mutSBE(2/3,6/7) embryo confirmed lack of vascular expression. Grey numbers in bottom right corner indicate the number of F0 mouse with similar expression patterns to the image shown. White and black scale bars both represent 100 μm, grey scale bars are 1000 μm. da = dorsal aorta, cv = cardinal vein, nt = neural tube; red bracket = dorsal artery; white bracket = axial veins. See also Supplementary Figure 5

We directly tested whether the Ephb4-2 enhancer was active in the absence of endothelial SMAD4 by generating *Smad4*^*EC/EC*^*;Tg(Ephb4-2:LacZ)* mouse embryos. *Ephb4-2:LacZ* expression was largely absent in E10.5 *Smad4*^*EC/EC*^*;Tg(Ephb4-2:LacZ)* embryos, similar to that seen with *Ephb4*^*LacZ*^ (Fig. 5a and Supplementary Fig. 5a–b). These results demonstrate that endothelial SMAD4 is required for both endogenous *Ephb4* expression and activity of the Ephb4-2 enhancer.

The R-SMAD/SMAD4 complexes recognize a variety of DNA sequences. These include the SMAD-binding element (SBE; Supplementary Fig. 4b), and a variety of GC-rich sequences primarily associated with the BMP-driven R-SMADS 1, 5 and 8^32,33^. The SBE was initially identified as a motif bound by human SMAD3 and SMAD4. However, SBE motifs are highly enriched within ChIP-seq and ChIP-chip peaks after immunoprecipitation with SMAD4^34,35^, with TGF-β-associated R-SMAD SMAD3^36,37^, and with BMP-associated R-SMADs SMAD1/5^32^ emphasizing the commonality of this motif in DNA regions bound by different types of R-SMAD/SMAD4 complexes. We therefore investigated whether the Ephb4-2 enhancer sequence contained any consensus or near-consensus SBEs (as defined by JASPAR^38^). This analysis identified nine potential binding motifs conserved between human and mouse sequences (Supplementary Fig. 4c–d). Mutation of all nine SBEs within the Ephb4-2 enhancer (Ephb4-2mutSBEall) resulted in a massive reduction in vascular expression in transgenic fish (Supplementary Fig. 5c–d). Of these nine SBE, four occurred as part of palindromic repeat SBE sequences (SBE2/3 and 6/7) commonly associated with SMAD binding^33^. Mutations of these SBE2/3 and SBE6/7 sites separately also resulted in near-total loss of the Ephb4-2 activity in transient transgenic mice and zebrafish (Fig. 5c and Supplementary Fig. 5c–e). Similar mutations to other conserved regions away from the SBEs and EBEs did not result in any alteration of expression (Supplementary Fig. 4c and 5c–d). In conclusion, these analyses demonstrate that both endogenous *Ephb4* and Ephb4-2 enhancer activity requires SMAD4 and indicate an essential role for SMAD4/R-SMAD-binding motifs for venous expression of the Ephb4-2 enhancer.

### SMAD1/5 binds *Ephb4-2* to influence *Ephb4* expression

SMAD4 forms heteromeric transcriptional complexes with multiple different phosphorylated R-SMADS prior to nuclear translocation and direct DNA binding^33^. In mice, the combined endothelial-specific deletion of the R-SMADs *Smad1* and *Smad5*, which are activated by BMP ligands, closely phenocopies the vascular defects and early lethality seen in *Smad4*^*EC/EC*^ embryos, although this study ascribed the defective vasculature to an angiogenic defect, not an arteriovenous one^15^. In contrast, combined endothelial-specific deletion of *Smad2/Smad3*, activated by TGF-β ligands, has no vascular effects at E10.5 and does not affect survival until after E12.5^16^. This suggests that SMAD4-mediated venous identity involves the BMP-activated R-SMADs SMAD1 and SMAD5 (commonly written as SMAD1/5). Although SMAD1 and 5 have high affinity for SBE motifs, they prefer alternative GC-rich binding motifs^39^. This was confirmed by Morikawa et al.^32^, who performed ChIP-seq analysis on HUVECs stimulated with high levels of BMP9 to phosphorylate SMAD1/5. This identified four over-represented motifs within SMAD1/5-bound regions (reproduced in Supplementary Fig. 6a), only one of which was localized around peak summits and therefore assumed to directly bind SMAD1/5. This motif, summarized as GG^A^/_C_GCC, was similar to the GCCG and GGCGCC SMAD1/5-binding motifs described elsewhere^39^. In total, these GC-rich elements (referred to as GC-SBE) were found in nearly half of all SMAD1/5-bound motifs. This analysis also found significant enrichment of the SBE motif within SMAD1/5-bound regions, and demonstrated that, when present, both SBE and GC-SBE motifs were required for BMP responsiveness^32^. While the spacer length between SBE and GC-SBE motifs varies between different SMAD1/5-bound sequences, a 5 bp spacer between the two motifs was over-represented in SMAD1/5-bound regions^32^.

**Fig. 6:**
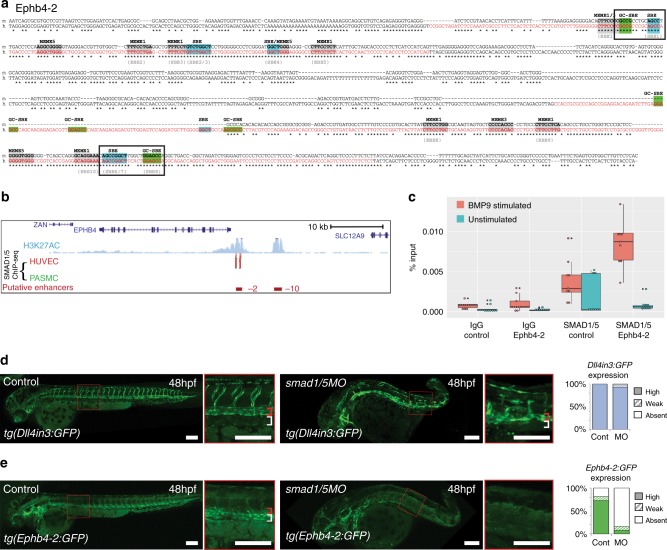
The Ephb4-2 enhancer is bound by SMAD1/5. **a** Mouse (m) and orthologous human (h) DNA sequence of the Ephb4-2 enhancer. Red nt on human sequence correspond to regions bound by SMAD1/5 as determined from SMAD1/5 human umbilical vein endothelial cell (HUVEC) chromatin immunoprecipitation-sequencing (ChIP-seq) data^32^ (see also Supplementary Fig. 6c). Green boxes denote GC-SBE motifs associated with SMAD1/5 binding, blue boxes denote SBE motifs associated with all SMAD binding and grey boxes denote other over-represented motifs (MEME1, 3 and 5^32^). Black outlined boxes denote composite SBE/GC-SBE elements, italic nt indicate linker sequences. Grey text under sequences indicates motifs previously identified as the EBEs and SBEs in earlier analysis (Supplementary Fig. 4). For sequence logos of motifs, see Supplementary Fig. 6b. **b** SMAD1/5 HUVEC and PASMC ChIP-seq data from Morikawa et al.^32^ (red peaks = statistically significant peaks after BMP9 stimulation in HUVEC). Significant binding peaks are not observed in PASMC after BMP4 stimulation (green). See also Supplementary Fig. 6c). **c** Box and whiskers plot of ChIP-qPCR data shows significant enrichment of SMAD1 binding at the Ephb4-2 enhancer in BMP9-stimulated HUVECS (red) compared to a control intergenic region on the same chromosome (*p* = 0.00015, paired two-tailed *t* test). SMAD1 binding at the Ephb4-2 enhancer region is not enriched over control in the absence of BMP stimulation (green) (*p* = 0.19212). No enrichment is observed between IgG control regions (*p* = 0.3154 and *p* = 0.19212, paired two-tailed *t* test). Horizontal lines = medians, boxes = interquartile range (IQR); vertical lines = minimal/maximal values (up to 1.5× IQR) and black dots = data points outside of 3× IQR. Data represents three biological replicates each with three technical replicates performed in triplicate. All data points were included in statistical analysis. **d**, **e** Morpholino (MO)-mediated partial knockdown of *smad1/5* on GFP expression in arterial *tg(Dll4in3:GFP)* (**d**) and venous *tg(Ephb4-2:GFP)* (**e**) transgenic zebrafish lines, using 0.5 ng *smad1* MO and 0.25 ng *smad5* MO. Graphs depict expression in all embryos, Dll4in3:GFP WT *n* = 58, MO *n* = 48; Ephb4-2:GFP WT *n* = 104, MO *n* = 84. High expression = solid colour, weak = pattern, absent = solid white. Zebrafish embryos shown are representative of the predominant phenotype, red bracket = dorsal aorta, white bracket = posterior cardinal and ventral vein(s). White scale bars represent 100 μm

To determine whether SMAD1/5 directly binds the Ephb4-2 enhancer, we examined the Ephb4-2 enhancer sequence for GC-SBE SMAD1/5-binding motifs adjacent to the previously identified SBE motifs. This analysis identified two conserved SBE/GC-SBE composite motif elements and one additional non-conserved SBE/GC-SBE motif element (Fig. 6a and Supplementary Fig. 6a). Strikingly, the 3’ conserved SBE/GC-SBE composite element contains the SBE6/7 motif that we previously demonstrated was required for Ephb4-2 enhancer activity (Fig. 5c). This SBE/GC-SBE composite element contains the optimal 5 bp spacer sequence separating the SBE and GC-SBE (Fig. 6a). The Ephb4-2 enhancer also contained numerous sequences correlating to the other three SMAD1/5-bound region-associated motifs (Fig. 6a and Supplementary Fig. 6a).

To further determine whether phosphorylated SMAD1/5 directly binds the Ephb4-2 sequence, we re-examined the HUVEC SMAD1/5-binding data generated by Morikawa et al.^32^. In agreement with our motif analysis, significant EC-specific binding peaks for SMAD1/5 were found directly over the two conserved regions of the orthologous human EPHB4-2 enhancer sequence (Fig. 6b and Supplementary 6b–c). The ability of SMAD1/5 to bind these enhancers was further confirmed by ChIP quantitative polymerase chain reaction (qPCR) using the same conditions as in Morikawa et al.^32^ (Fig. 6c). No other SMAD1/5-binding peaks were found elsewhere in the *Ephb4* gene locus (Supplementary Fig. 6b).

To determine whether reduction in SMAD1/5 levels affected Ephb4-2 activity, we examined the consequences of morpholino (MO)-mediated *smad1/5* knockdown on Ephb4-2:GFP in transgenic zebrafish. While the effects of complete deletion of *smad1/5* on Ephb4-2 could not be investigated owing to severe patterning defects^40^, we were able to knockdown *smad1/5* in *tg(Ephb4-2:GFP)* zebrafish using lower doses of *smad1* and *smad5* MO^40^. These MOs adhere to current guidelines^41^, in that they recapitulate the mutant phenotype, give similar results to other MOs targeting the same gene and can be rescued by RNA expression^40^. Knockdown of *smad1/5* resulted in near-complete ablation of Ephb4-2:GFP activity in the trunk and segmental vessels (Fig. 6d and Supplementary Fig. 7a). All embryos also had a dorsalization defect that significantly affected the tail region and grew more severe with high MO doses, invalidating any analysis of venous structures in these morphants. However, expression of the arterial-specific Dll4in3:GFP transgene was still robust in the tail region of MO-treated embryos, suggesting that a pan-vascular defect was unlikely to explain the loss of Ephb4-2:GFP expression. A dose–response curve (with total MO concentration ranging from 0.375 to 1.5 ng), demonstrated both increased dorsalization and decreased Ephb4-2:GFP activity as MO concentrations increased (Supplementary Fig. 7b–c). Loss of Ephb4-2:GFP after *smad1/5* knockdown was also unlikely to be related to reduction of blood flow, as Ephb4-2:GFP expression was not significantly affected in *tnnt2* morphants, which have no heartbeat (Supplementary Fig. 7d). Whole-mount in situ analysis in these morphants was used to also examine the expression patterns of endogenous *ephb4a*, *dll4* and the arterial marker *ephb2a*: arterial *dll4 and ephb2a* was detected in *smad1/5* morphants, while *ephb4a* expression was greatly reduced (Supplementary Fig. 8). Although it is unwise to make definitive conclusions based on MO-based knockdown analysis alone, these results were strikingly similar to those seen in *Smad4*^*EC/EC*^ mice. Consequently, when combined with enhancer sequence analysis, ChIP-seq and ChIP-qPCR results, our data strongly support a direct role for SMAD1/5–SMAD4 in the regulation of venous *Ephb4* expression.

### SMAD1/5 regulates transcription of venous-specific genes

Although phosphorylated SMAD1/5 (pSMAD1/5) can be detected in both venous and arterial ECs^42^ (Supplementary Fig. 9a), the Id1 promoter-based BMP response element (BRE, containing SBE and GC-SBE motifs)^43^ has been reported to be preferentially expressed in the zebrafish caudal vein comparative to the dorsal aorta^44,45^ and is more strongly and consistently expressed in mouse cardinal vein ECs (E11.5) and postnatal retinal veins (P4) comparative to arterial ECs^46^. Further, analysis of the HUVEC SMAD1/5 ChIP-seq data by Morikawa et al.^32^ found significant SMAD1/5 binding within only two of the 11 in vivo-characterized pan-endothelial enhancers (Supplementary Fig. 9b), and no SMAD1/5-binding peaks within any of the six known arterial gene enhancers (Supplementary Fig. 9c). This suggests that SMAD1/5 binding is not strongly associated with pan-EC or arterial gene expression, even though HUVECs express many arterial-associated genes^47^, including the BMP type I receptor ALK1 (ACVRL1) through which BMP9 can signal^32,48^. This result therefore indicates that, unlike ETS factors, SMAD1/5 binding is not an essential component of all endothelial enhancers.

**Fig. 7:**
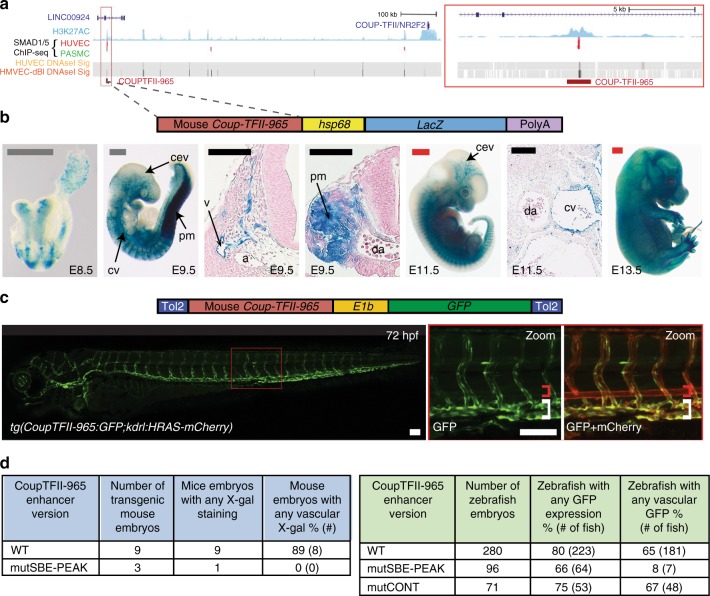
A SMAD1/5-binding peak identifies a vein endothelial enhancer for *Coup-TFII*. **a** UCSC browser view (http://genome.ucsc.edu) of *COUP-TFII (NR2F2)* incorporating SMAD1/5-binding data from Morikawa et al.^32^. A statistically significant SMAD1/5-binding peak in human umbilical vein endothelial cells (HUVECs) (red) −965 kb upstream of Coup-TFII correlated with the H3K27Ac enhancer histone mark in HUVECs (blue peaks) and endothelial HUVEC DNaseI hypersensitivity (black heat map). **b**, **c** Stable transgenic mouse (**b**) and zebrafish (**c**) embryos expressing the *lacZ* and GFP reporter genes, respectively, under the control of the murine CoupTFII-965 enhancer. In both animal models, enhancer activity was primarily seen in the venous endothelium. Black and white scale bars both represent 100 μm, grey scale bars are 500 μm, red scale bars are 1000 μm. a = artery, v = vein, cev = branches of cerebral venous plexus, cv = cardinal vein, pm = paraxial mesoderm, da = dorsal aorta, red bracket = dorsal aorta; white bracket = posterior cardinal and ventral vein(s). **d** Tables summarizing reporter gene expression in transient transgenic zebrafish and mouse embryos after mutation of the core SMAD-binding element (SBE-PEAK). See also Supplementary Figures 10–11

The loss of venous structures in *Smad4*^*EC/EC*^ embryos suggests the possibility that SMAD4–SMAD1/5 binding may be a shared feature of venous-expressed genes. Supporting this hypothesis, our re-examination of the HUVEC SMAD1/5 binding^31^ found SMAD1/5-bound peaks within the loci of the venous-associated *Coup-TFII (Nr2f2)*, *Nrp2* and *Emcn* genes (Fig. 7a and Supplementary Fig. 10a). In each case, SMAD1/5 binding coincided with enhancer-associated histone marks, DNaseI hypersensitivity and sequence motifs for both ETS (EBE) and SMAD (SBE and GC-SBE) (Fig. 7a and Supplementary Fig. 10a–b), suggesting that these sequences may represent endothelial enhancers. Supporting this hypothesis, the CoupTFII-965, Nrp2+26 and EMCN-22 enhancers were all able to direct endothelial-specific *LacZ* activity in transgenic mice (Fig. 7b and Supplementary Fig. 10c–d).

*Coup-TFII*, like *Ephb4*, is specifically expressed in venous but not arterial ECs, where it is essential for vein acquisition and identity in early embryonic development^7^. Similar to the Ephb4-2 enhancer, the CoupTFII-965 enhancer was able to drive venous endothelial-specific reporter gene expression in both transgenic mice and transgenic zebrafish lines (Fig. 7b, c and Supplementary Fig. 11a–c). Unlike Ephb4-2, CoupTFII-965 expression was also seen in the early dorsal aorta (E8.5 and 24 hpf) and paraxial mesoderm (E9.5 and 24 hpf) in both mouse and zebrafish transgenic models (Fig. 7b and Supplementary Fig. 11a), suggesting that CoupTFII-965 may bind additional factors not shared with Ephb4-2 that regulate non-venous endothelial activity. Although we could not independently verify SMAD1/5 binding to the CoupTFII-965 enhancer region, analysis of the CoupTFII-965 enhancer sequence identified three conserved consensus SBE-binding motifs, the second of which was located within a core SMAD1/5-binding peak (Supplementary Fig. 10b and 11d). Unlike Ephb4-2, no conserved GC-SBE motifs were found surrounding these SBE sites, although non-conserved GC-SBE were separately identified in both human and mouse sequences (Supplementary Fig. 11d). To determine whether the core SBE (SBE-peak) was required for CoupTFII-965 activity, we tested enhancers in which this motif was mutated. Expression of the CoupTFII-965 enhancer was entirely ablated after mutation of SBE-peak in both mouse and zebrafish transient transgenic models (Fig. 7d). As with Ephb4-2, alternative mutations within the CoupTFII-965 enhancer away from the EBEs and SBEs did not significantly influence activity (Fig. 7d). Therefore, although these results cannot prove SMAD1/5 binding to the COUPTFII enhancer, they suggest that SMAD-mediated activation may be a shared feature of both *Ephb4* and *Coup-TFII* gene expression in veins.

### The BMP type I receptor ALK3 is required for vein formation

**Fig. 8:**
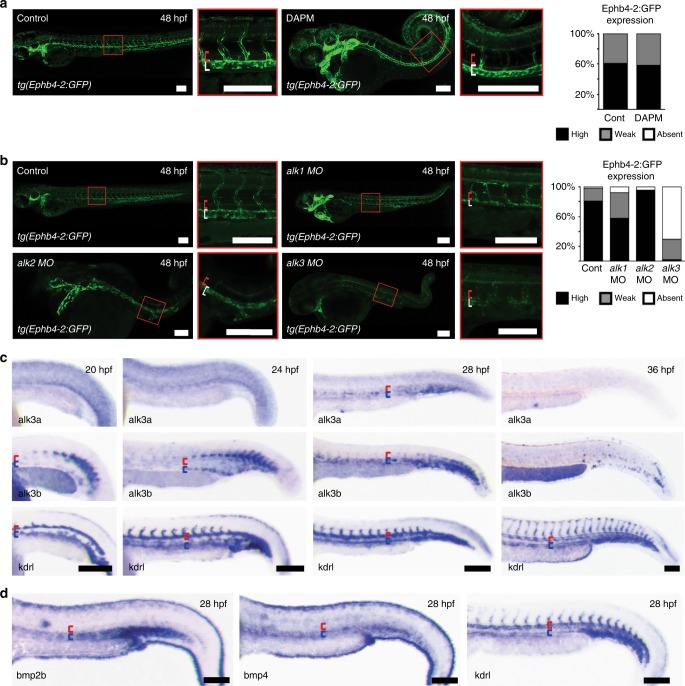
Notch-independent Alk3a/b signalling is involved in venous identity in zebrafish. **a** Loss of Notch signalling had no effect on the expression of the venous Ephb4-2:GFP transgene in *tg(Ephb4-2:GFP)* transgenic zebrafish. Representative 48 hpf embryos demonstrate similar intensities of vein-specific green fluorescent protein (GFP) expression in both control and DAPM-treated embryos. Red bracket = dorsal aorta, white bracket = posterior cardinal and ventral vein. Graph depicts observed expression pattern of GFP in *tg(Ephb4-2:GFP)* embryos for control (*n* = 51) and 100 µM DAPM-treated embryos (*n* = 57); black denotes high expression, grey denotes weak. See Supplementary Fig. 12 for Coup-TFII-965:GFP results and controls. **b** Morpholino (MO)-induced reduction of *alk1* and *alk2* had little effect on Ephb4-2:GFP expression in 48hpf *tg(Ephb4-2:GFP)* transgenic zebrafish, whereas reduction of alk3a/b resulted in significantly decreased transgene expression. Representative 48 hpf *tg(Ephb4-2:GFP)* embryos demonstrate reduced GFP expression after alk3a/b MO injection. Red bracket = dorsal aorta, white bracket = posterior cardinal and ventral vein. Graph depicts observed expression patterns of GFP for control (*n* = 51), *alk1 MO* (*n* = 45), *alk2 MO* (*n* = 45) and *alk3a/b MO* (*n* = 48); black denotes high expression, grey denotes weak expression and white denotes no detectable GFP expression. **c** Whole-mount in situ hybridization for bone morphogenetic protein (BMP) receptors *alk3a* and *alk3b* compared to pan-endothelial *kdrl* in wild-type zebrafish embryos at 20, 24, 28 and 36 hpf. Earlier time points can be seen in Supplementary Fig. 13. Both *alk3a* and *alk3b* were detected in the axial vein but not in the artery, with greater early expression seen for *alk3b*. Red bracket = axial artery, blue bracket = axial vein. **d** Whole-mount in situ hybridization for BMP ligands *bmp2b* and *bmp4* compared to pan-endothelial *kdrl* in wild-type zebrafish embryos at 28 hpf. Both bmp2b and bmp4 showed stronger expression around the axial veins comparative to the dorsal artery. Red bracket = axial artery, blue bracket = axial vein. All scale bars represent 100 μm. See also Supplementary Figures 12–13

We hypothesized that either repression of BMP-driven gene activation in early arterial ECs or spatially selective expression of BMP receptors may contribute to venous-specific gene expression. We found no evidence of arterial repression of BMP-driven venous gene activation via known pathways. Specifically, chemical inhibition of ERK/MAPK signalling, which is active in arteries but repressed in veins^6^ and had previously been associated with inhibition of nuclear SMAD1^49^, did not lead to arterial expansion of Ephb4-2:GFP expression (Supplementary Fig. 12a). Similarly, Notch signalling can repress BMP responsiveness through the activation of SMAD6 in the angiogenic sprout^50^, yet inhibition of Notch had no effect on the intensity and vein specificity of Ephb4-2:GFP and CoupTFII-965:GFP expression, although the expected hyper-sprouting phenotype was observed (Fig. 8a and Supplementary Fig. 12b)^51^. We also found little evidence that spatial restriction of BMP type II receptors played a crucial role in early venous identity. Wiley et al. demonstrated that zebrafish BMP type II receptors bmpr2a and bmpr2b (BMPR2 in mice) are important regulators of venous angiogenic sprouting^18^. However, when we repeated their MO-induced depletion of *bmpr2a* and *bmpr2b* in *tg(Ephb4-2:GFP)* zebrafish, we did not lose enhancer activity and could still see a developed axial vein although we recapitulated the venous sprouting defect (Supplementary Fig. 12c). Similarly, Kim et al.^44^ reported an essential role for *dab2* as a mediator of BMP2 signalling in zebrafish venous sprouting. However, again when we repeated their MO-induced depletion of *dab2* in *tg(Ephb4-2:GFP)* zebrafish we saw little change in enhancer activity or axial vein formation although we again recapitulated the venous sprouting defect (Supplementary Fig. 12c). While the initial design of these MOs may not meet the current standards in all cases, mice lacking endothelial BMPR2 also exhibit no reported embryonic vascular defects^39^, strongly suggesting that BMPR2 is unlikely to be absolutely required for early *Ephb4* expression or venous identity. Since the other BMP type II receptors, *Acvr2a* and *Acvr2b*, are ubiquitously expressed during arteriovenous differentiation^52^, it is therefore unlikely that spatial restriction of type II receptors alone is responsible for vein identity during embryonic development.

To investigate a potential role of BMP type I receptors in *Ephb4* expression, we first investigated Ephb4-2:GFP activity after addition of the chemical inhibitor DMH1. DMH1 specifically targets the BMP type I receptors ALK1 (ACVRL1), ALK2 (ACVR1), ALK3 (BMPR1A) and ALK6 (BMPR1B). Treatment of *tg(Ephb4-2:GFP)* embryos with DMH1 (at a dose that did not cause severe patterning defects) resulted in significantly reduced Ephb4-2:GFP activity (Supplementary Fig. 12d), suggesting a requirement for one or more of these receptors in venous identity. Unlike the early vascular defects and lethality seen in *Smad4*^*EC/EC*^ and *Smad1/5*^*EC/EC*^ mice, *Alk6* null mice are born healthy at Mendelian ratios with no reported vessel defects^53^. Consequently, we ruled out a potential essential role for ALK6 in embryonic vein development. However, individual loss of *Alk1*, *Alk2* and *Alk3* in mice each results in embryonic lethality with some degree of vascular defects^12,54,55^, warranting further investigation.

To determine which ALK receptor(s) are required for venous identity, we first conducted a zebrafish screen, looking at the effects of reducing levels of the zebrafish orthologues of ALK1, 2 and 3 individually in *tg(Ephb4-2:GFP)* fish using MOs known to recapitulate mutant phenotypes. These experiments used reduced, sub-lethal concentrations of MOs where necessary to avoid previously reported gastrulation defects^56,57^, effectively creating a knockdown but not full depletion. The *alk1* and *alk2* morphants maintained reporter gene activity in the posterior cardinal vein despite considerable morphological defects (Fig. 8b). In contrast, Ephb4-2:GFP expression was severely diminished in combined *alk3a/b* morphants (Fig. 8b and Supplementary Fig. 13a). The *alk3a* and *alk3b* MO used here recapitulate the zygotic mutant phenotype and can be rescued by RNA injection^56,58^. As with *smad1/5* MO, a dose–response curve was also included in the analysis (Supplementary Fig. 13a–b).

The loss of Ephb4-2:GFP expression in *alk3a/b* morphants suggested a potential role in venous gene expression. We therefore investigated the expression pattern of *alk3a/b* in early zebrafish embryos. Although not previously reported to be strongly expressed in the zebrafish vasculature^58^, we detected expression of *alk3b* in venous positioned angioblasts from 18 to 20 s (approximately 18 hpf), and both *alk3a* and *alk3b* were highly expressed in venous regions of the axial vasculature by 28 hpf (Fig. 8c and Supplementary Fig. 13c). In comparison, we detected little expression of endogenous *ephb4a* until 20 s (approximately 19–20 hpf; Supplementary Fig. 13d), suggesting *alk3a/b* expression begins concurrently or just before that of *ephb4a*. The expression of known ALK3 ligands *bmp2b* and *bmp4* were also highest around the developing trunk vein^18^ (Fig. 8d). Further, MO-induced depletion of *alk3a/b* resulted in significant reduction in endogenous *ephb4a* expression (Supplementary Fig. 14a–c), while morphological analysis of *alk3a/b* morphants demonstrated the presence of only one axial vessel and a lack of proper blood circulation (Supplementary Fig. 14d). These results therefore suggest that the spatially restricted ALK3 receptors may play a fundamental role in *ephb4* expression and vein morphogenesis in zebrafish.

**Fig. 9:**
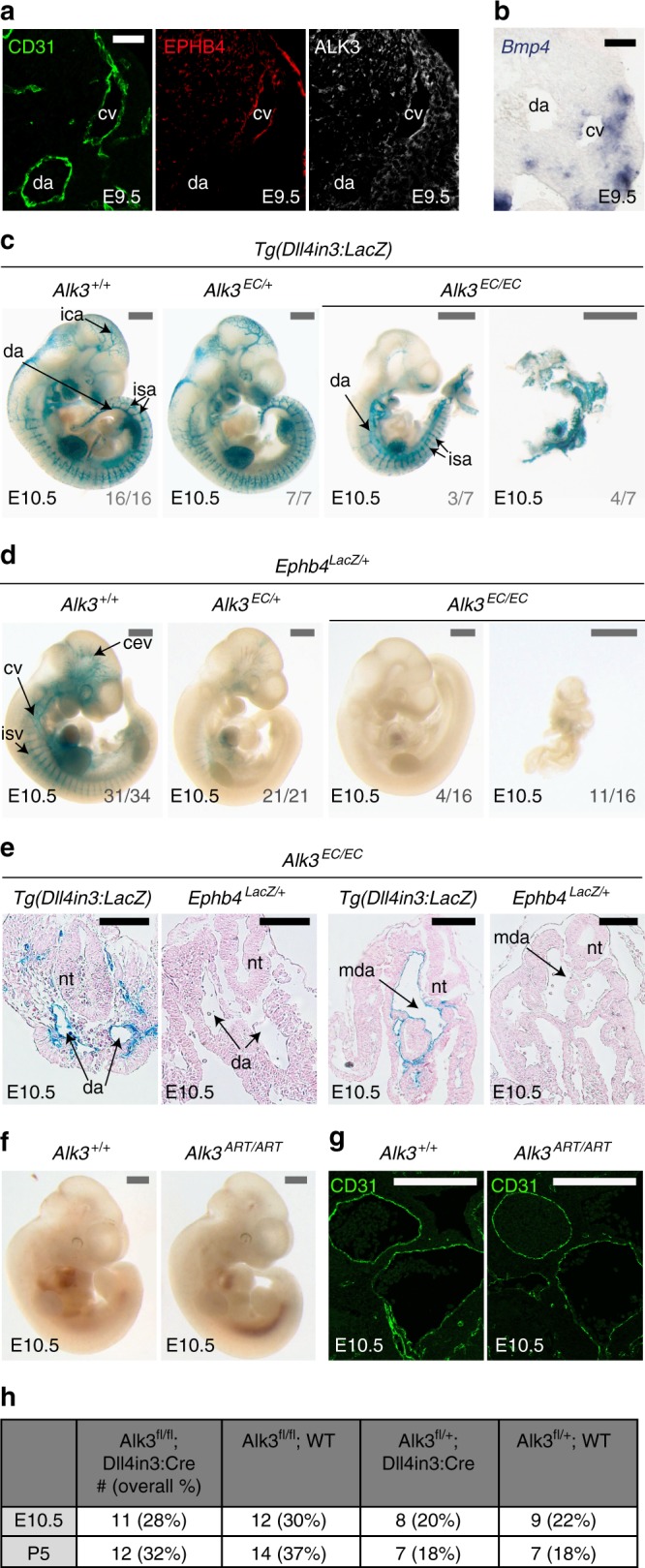
Endothelial-specific knockout of *Alk3* results in loss of venous identity in mice. **a**, **b**. Immunofluorescent analysis of CD31, EPHB4 and ALK3 (**a**) and in situ hybridization for *Bmp4* (**b**) in transverse sections from E9.5 mouse embryos. Scale bars are 50 μm. **c** Representative E10.5 whole-mount images from wild-type *Alk3*^*+/+*^ (*n* = 16), heterozygous *Alk3*^*EC/+;*^ (*n* = 7) and homozygous *Alk3*^*EC/EC*^ (*n* = 7) embryos expressing the arterial Dll4in3:LacZ transgene (Nine litters). Robust transgene expression, specific to arterial endothelial cells, was seen in all embryos although *Alk3*^*EC/EC*^ embryos were often significantly growth retarded. Grey numbers on bottom right denote the number of embryos similar to picture shown; for *Alk3*^*EC/EC*^, two different images show Dll4in3:LacZ expression in the range of morphological defects. Grey scale bars are 500 μm. **d** Representative E10.5 whole-mount images from wild-type *Alk3*^*+/+*^ (*n* = 34), heterozygous *Alk3*^*EC/+;*^ (*n* = 21) and homozygous *Alk3*^*EC/EC*^ (*n* = 16) embryos expressing venous *Ephb4*^*LacZ*^ (21 litters, for littermates, see Supplementary Fig. 16). Robust X-gal activity is detected in the veins of *Alk3*^*+/+*^ embryos but is reduced in *Alk3*^*EC/+*^ embryos and absent in *Alk3*^*EC/EC*^ regardless of extend of growth retardation and morphological defects. Grey numbers on bottom right denote the number of embryos similar to picture shown; for *Alk3*^*EC/EC*^, two different images are shown to indicate *Ephb4*^*LacZ*^ expression in the range of morphological defects. Grey scale bars are 500 μm. **e** Representative transverse sections from E10.5 *Alk3*^*EC/EC*^ embryos transgenic for either arterial Dll4in3:LacZ or venous *Ephb4*^*LacZ*^ at two different levels. Some vessels were clearly seen in both; these expressed Dll4in3:lacZ but not *Ephb4*^*LacZ*^ and were located in arterial positions, suggesting the presence of dorsal aorta but no cardinal vein. Black scale bars are 100 μm. **f**, **g** Representative arterial endothelial-specific *Alk3*^*+/+*^ and *Alk3*^*ART/ART*^ whole-mount E10.5 embryos (**f**) and transverse sections stained with CD31 (**g**). Loss of *Alk3* in arterial endothelial cells had no effect on vascular development at E10.5. White scale bars are 100 μm. **h** Observed frequency of *Alk3*^*fl/fl*^*;Dll4in3:Cre* embryos at E10.5 and P5. Mendelian ratios were present at both time points. For all panels, ^EC^ is Tie2:Cre-mediated deletion, ^ART^ is Dll4in3:Cre-mediated deletion. ^+/+^ indicates Cre−, ^EC/+^ indicates Cre+,Alk3^fl/+^ and ^EC/EC^ indicates Cre+;Alk3^fl/fl^. da = dorsal aorta, ica = internal carotid artery, isa = intersomitic arteries, isv = intersomitic vessels, baa = branchial arch arteries, nt = neural tube, cv = cardinal vein, cev = branches of cerebral venous plexus, mda = midline dorsal aorta

**Fig. 10:**
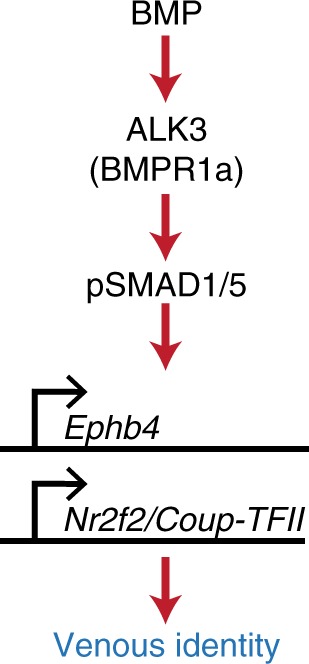
Proposed model. Our data support a model in which vein-enriched bone morphogenetic protein (BMP) ligands BMP2 and BMP4 signal through the vein-specific Alk3 type I receptor (in combination with multiple different BMP type II receptors) resulting in the phosphorylation of SMAD1/5, transcriptional activation of the Ephb4 and Coup-TFII genes and subsequent venous identity

Endothelial-specific loss of *Alk3 (Bmpr1a)* in mice results in early vascular defects similar to *Smad4*^*EC/EC*^ embryos^12^, supporting a specific role for ALK3 in venous identity in mammals as well as zebrafish. Comparatively, endothelial deletion of the vascular type I receptors *Alk1(Acvrl1)* and *Alk2 (Acvr1)* caused later lethality^55,59^. Analysis of murine ALK3 expression also found that it was preferentially found in venous ECs relative to arterial endothelium at E8.5 and E9.5 (Fig. 9a and Supplementary Fig. 15a). Further, expression of the ALK3 ligand *Bmp4* was also preferentially found around venous vessels (Fig. 9b and Supplementary Fig. 15b). Although the vascular defects seen after endothelial-specific *Alk3* deletion were previously attributed to defective vessel maturation and problems in atrioventricular endocardial cushion formation^12^, arteriovenous differentiation was not investigated in these embryos. Therefore, we re-investigated the consequences of endothelial-specific ablation of *Alk3* in mice. As with *Smad4*^*EC/EC*^ embryos, Tie2:Cre-mediated deletion of *Alk3* (*Alk3*^*EC/EC*^) resulted in severe defects by E10.5 but did not ablate expression of the arterial-associated Dll4in3:*LacZ* transgene (Fig. 9c, e). However, no expression of *Ephb4*^*LacZ*^ was detected in any *Alk3*^*EC/EC*^ embryos by E10.5 (Fig. 9d, e). Morphological analysis of *Alk3*^*EC/EC*^*;Dll4in3:LacZ* and *Alk3*^*EC/EC*^*;Ephb4*^*LacZ*^ embryos confirmed that these embryos contain Dll4in3:LacZ+ dorsal aortas but lack both *Ephb4*^*LacZ*^-positive vessels (Fig. 9e). Although many of these embryos were substantially defective by E10.5, all heterozygous *Alk3*^*EC/+*^ embryos also showed consistently reduced levels of *Ephb4*^*LacZ*^ expression, although they exhibited no clear morphological defects (Fig. 9d). Strikingly, deletion of *Alk3* using the arterial expressed Dll4in3:Cre (*Alk3*^*ART/ART*^) had little effect on embryos at E10.5 (Fig. 9f–h), demonstrating that, similar to *Smad4*^*EC/EC*^, the vascular defects seen in *Alk3*^*EC/EC*^ embryos are not caused by general vascular maturation or cardiac defects. Previous analysis also found no defects in haematopoiesis in *Alk3*^*EC/EC*^ embryos^12^. Because the ALK3 receptor is specific for the BMP pathway and signals only through SMAD1/5/8^60^, these mutant mouse experiments also provide direct in vivo murine evidence supporting our zebrafish *smad1/5* MO-mediated observations linking SMAD1/5 with the regulation of Ephb4 and venous identity. In conclusion, these results support a model of arteriovenous development in which the venous-restricted ALK3 receptor is indispensable for vein morphogenesis downstream of BMP ligands and upstream of SMAD4–SMAD1/5-dependent transcriptional activation of venous genes (Fig. 10).

## Discussion

The crucial roles for VEGF-A and NOTCH in activating arterial EC identity has often led to the supposition that venous specification is either default or actively repressed in arteries. However, our results clearly demonstrate that venous genes are directly transcriptionally activated via a BMP/ALK3/SMAD1/5 signalling cascade. This therefore supports a model for arteriovenous differentiation in which endothelial progenitors positively acquire either arterial or venous fate downstream of independent signalling pathways early in development.

The specific expression of the Alk3 type I receptor and Bmp2/4 ligands in venous ECs suggests that venous ECs may be innately sensitive to BMP signalling^45^. Supporting this, the type II BMP receptors Bmpr2a and Bmpr2b and cargo-specific adaptor Dab2 are also enriched in zebrafish venous ECs^18,44^. Although neither BMPR2a/b nor DAB2 appeared to be absolutely required for *Ephb4-2* activity or venous specification, the combined expression of these BMP signalling components in addition to ALK3 may well contribute to an increased venous endothelial response to BMP ligands. It is also possible that the type I receptor ALK2 (ACVRL1) contributes to venous differentiation at later stages of embryogenesis and after birth: while endothelial-specific loss of *Alk2* did not phenocopy the early vascular defects seen in *Smad4*^*EC/EC*^ or *Alk3*^*EC/EC*^ embryos, ALK2 is implicated in venous angiogenesis alongside ALK3 in zebrafish^18^ and is strongly expressed in venous ECs in the postnatal retina, where *Alk2* and *Alk3* are required for correct retinal vessel morphogenesis^61^.

The results in this paper strongly indicate that the establishment of venous identity is the principle function for BMP signalling in the early vasculature. In particular, the absence of detectable vascular defects in E10.5 *Smad4*^*ART/ART*^ embryos suggests that many of the previous roles attributed to BMP signalling via SMAD4 in ECs, including vascular integrity, remodelling and smooth muscle recruitment^12,14^, may be secondary to the loss of correct arteriovenous specification and resultant breakdown of vascular patency. While we did not directly investigate cardiac valve defects, both *Alk3*^*ART/ART*^ and *Smad4* ^*ART/ART*^ embryos survived until late gestation despite endocardial expression of Dll4in3:Cre, indicating that the requirement for BMP signalling in cardiac valve formation may manifest in death at later embryonic stages. This does not necessarily suggest that BMP signalling is unimportant for valve morphogenesis: endocardial nuclear factor of activated T cell (NFAT) signalling is absolutely required for heart valve morphogenesis, yet endothelial NFAT-deficient embryos display aberrant valves and gestational lethality only after E13.5^62^. Similarly, our results do not directly contradict a role for BMP-SMAD1/5 in angiogenic sprouting^15,22^. While the Dll4in3:Cre used to generate *Smad4*^*ART/ART*^ embryos is also active in ECs at the angiogenic front, Dll4in3 is preferentially active in tip cells^21^. Conversely, the angiogenic role of BMP signalling is thought to occur primarily in stalk cells, where it co-operates with the Notch pathway^15,22^. Agreeing with this role, endothelial SMAD1/SMAD5 binding was also enriched around Notch pathway genes, although not over the three characterized arterial Notch-pathway enhancers (this paper and ref. ^32^). However, recent reports in zebrafish demonstrate that venous ECs are the primary source of cells at the angiogenic front^63^, suggesting that it may be impossible to entirely separate the requirement of SMAD1/5 in venous identity with any potential role in embryonic angiogenic sprouting.

SMAD1/5 is involved in many other BMP-driven processes beyond the vasculature, suggesting that any recruitment of SMAD4–SMAD1/5 to venous enhancers would require additional cofactors^33,64^. The presence of ETS-binding motifs (EBEs) alongside SMAD motifs in each venous enhancer suggests a role for ETS transcription factors such as ERG, which are already established as central regulators of endothelial gene expression^25^, in conferring endothelial specificity to SMAD1/5-bound vein enhancers. These may potentially assist SMAD complexes in binding DNA, as alone they have only low affinity for DNA^33^. However, ETS factor binding is also a feature of pan-endothelial, arterial and angiogenic enhancers^19–21^, making it unlikely that ETS factors contribute directly to venous specificity.

Although ligand and receptor density or specificity may result in increased venous pSMAD1/5 during early arteriovenous development^18^, some BMP receptors are clearly expressed in both mouse and zebrafish arteries (e.g. ALK1)^65,66^, and HUVEC SMAD1/5 ChIP analyses demonstrate that BMP9, more commonly associated with ALK1-mediated angiogenesis, can stimulate SMAD1/5 to bind to venous enhancers in vitro. Nuclear pSMAD1/5 is also found in both arterial and venous ECs, yet both Ephb4-2 and CoupTFII-965 enhancers were predominantly specific to venous endothelium. It is therefore highly probable that additional co-factors cooperate with ETS and SMAD1/5 to activate specific genes in veins and/or to repress these genes elsewhere. There is already some evidence supporting a role for direct transcriptional activation and repression in SMAD1/5-dependent vein-specific gene expression: the Id1-based BRE, effectively a group of SBE and GC-SBE motifs, is virtually silent in the vasculature unless additional cytomegalovirus (CMV)-derived enhancer elements are added to the transgene^67^. Further, activity of the BRE-CMV is enriched but not specific to the venous endothelium. These observations therefore suggest that DNA motifs binding both activating and repressive transcription factors may be needed in addition to SBE and GC-SBE elements to achieve vein-specific gene activation.

Our results also present a compelling case for ALK3 to be considered as a target for antiangiogenic therapy. Current antiangiogenic drugs, which aim to prevent the rapid vessel growth seen during tumourigenesis, primarily target the VEGF signalling pathway. However, the response is often limited and additional therapeutic targets are needed^68^. Combined with the emerging understanding of the importance of the venous endothelium at the angiogenic front^63^, the essential and independent role for ALK3 in venous growth demonstrated here suggests that targeting of ALK3 may effectively inhibit tumour angiogenesis.

## Methods

### Cloning

For venous enhancers, all enhancer sequences were initially generated as custom-made, double-stranded linear DNA fragments (GeneArt® Strings™, Life Technologies) with the exception of Coup-TFII-965 and Nrp2+26, which were generated by PCR from genomic DNA. The sequences of all enhancers are provided in Supplementary Methods. DNA fragments/PCR products were cloned into the pCR8 vector using the pCR8/GW/TOPO TA Cloning Kit (Invitrogen, K2500-20) following the manufacturer’s instructions. Once cloning was confirmed, the enhancer sequence was transferred from the pCR8/GW/enhancer entry vector to a suitable destination vector using Gateway LR Clonase II Enzyme mix (Life Technologies, 11791-100) following the manufacturer’s instructions. For mouse transgenesis, the enhancer was cloned into the hsp68-LacZ-Gateway vector (provided by N. Ahituv). For zebrafish transgenesis, the enhancer was cloned into the E1b-GFP-Tol2 vector (provided by N. Ahituv).

To generate the Dll4in3Cre transgene, the Dll4in3 enhancer^19^ was amplified by PCR to include engineered 5’ SacII and 3’ Not1 restriction sites (sequences in Supplementary table 1) and cloned into the promoterless p-AUG-βGal construct^69^. The Dll4in3 promoter was also generated by PCR with engineered 5’ SpeI and 3’ BamHI sites (sequences in Supplementary Methods) and cloned into the Dll4in3 enhancer–p-AUG-βGal construct. Transgenic mice were generated to ensure activity of enhancer/promoter construct, after which the βGal sequence was replaced by Cre-ORT/polyA amplified from the pCAG-Cre vector. The full sequence of the Dll4in3 enhancer/promoter is provided in Supplementary Methods.

### Mice

All animal procedures comply with all relevant ethical regulations, were approved by the Clinical Medicine Local Ethical Review Committee, University of Oxford and licensed by the UK Home Office. *Smad4*^*fl/fl*^ mice^70–73^ were provided by Elizabeth Robertson, *Tg(Tie2:Cre)* mice, officially designated *Tg(Tek-cre)*^12Flv/J^^74,75^, were purchased from JAX (stock number 004128), *Ephb4*^*LacZ/+*^ mice^5,76^ were provided by David Anderson, Cdh5(PAC)-CreERT2 mice^23^ were provided by Ralf Adams and *Alk3*^*fl/fl*^ mice^12^ were provided by Yuji Mishina. *Tg(Dll4in3:LacZ)* were as described in refs. ^19,32^, while the *Tg(Ephb4-2:LacZ)*, *Tg(Coup-TFII-965)* and *Tg(Dll4in3:Cre)* lines were generated for this paper. Transgenic mice were generated by oocyte microinjection of linearized DNA^77,78^. Mouse embryos were collected along with yolk sac and fixed in 2% paraformaldehyde (PFA), 0.2% glutaraldehyde and 1× phosphate-buffered saline (PBS). E8.5 embryos were fixed at 4 °C for 10 min, E9.5 embryos fixed for 30 min, E11.5 embryos for 60 min and E13-E15 embryos for 120 min. After fixation, embryos were rinsed in 0.1% sodium deoxycholate 0.2% Nonidet P-40 2 mM MgCl_2_ 1× PBS and then stained for 2–24 h in 1 mg/ml 5-bromo-4-chloro-3-indolyo-β-D-galactoside solution (X-gal) containing 5 mM potassium ferrocyanide, 5 mM ferricyanide, 0.1% sodium deoxycholate, 0.2% Nonidet P-40, 2 mM MgCl_2_ and 1× PBS. After staining, embryos were rinsed through a series of 1× PBS washes, then fixed overnight in 4% PFA at 4 °C. Embryos were imaged using a Leica M165C stereo microscope equipped with a ProGres CF Scan camera and CapturePro software (Jenoptik). In instances that images have been altered to improve quality and colour balance, each image within a set (e.g. WT, het and null embryos) have been altered using the same parameters. This occasionally included to selective depletion of the yellow or red colour channel, in order to counteract issues from the X-gal stain solution (which is orange). All embryos are stored in 4% PFA indefinitely and slowly become less yellow. Consequently, embryos stained more recently have a greater yellow/orange hue. An example of this alteration can be seen in Supplementary Figure 16.

The yolksac was used for genotyping. Tissue samples were incubated overnight at 55 °C with 500 μl GNT buffer (50 mmol/l KCl, 1.5 mmol/l MgCl_2_, 10 mmol/l Tris-pH8, 0.01% gelatin, 0.45% nonidet P40, 0.45% Tween) and proteinase K (10 mg/ml); 0.5 μl of this supernatant was subsequently used in PCR reactions with the GoTag Green master mix (Promega, M7122) using relevant PCR primers. For *Smad4*^*EC/EC*^ crosses with *Ephb4*^*Lacz/+*^ and *Ephb4-2:LacZ*, in the rare cases where the LacZ genotyping results did not agree with the pattern of X-gal staining in WT embryos, error was presumed and the entire litter was excluded. For histological analysis of transgenic and mutant mouse sections, embryos were dehydrated through a series of ethanol washes, cleared by xylene and paraffin wax-embedded. Five or 6-μm sections were prepared and de-waxed. For imaging of X-gal staining, slides were counterstained with nuclear fast red (Electron Microscopy Sciences). Analysis was qualitative not quantitative, therefore no statistical analysis was applied to the observations of staining intensity and pattern. Numbers of transgenic mice used followed precedent set by similar published papers. Where significant variations in expression were detected within a single experimental set, this is represented in the relevant figure by representative pictures of each outcome in combination with the *n* number for each example. No experimental randomization or blinding was used as this was not considered necessary.

### Transgenic zebrafish

All animal procedures were approved by local ethical review and licensed by the UK Home Office. *tg(Dll4in3:GFP)* and *tg(kdrl:HRAS-mCherry*) fish lines were as previously described^19,32,79^. The stable lines *tg(Ephb4-2:GFP*) and *tg(Coup-TFII-965)* were generated from an initial outcross of adult F0 carriers and intercrossed with the *tg(kdrl:HRAS-mCherry)* to enable visualization of the entire vasculature. F0 transient mosaic transgenic zebrafish embryos were generated using Tol2-mediated integration^80^. Embryos were maintained in E3 medium (5 mM NaCl; 0.17 mM KCl; 0.33 mM CaCl_2_; 0.33 mM MgSO_4_) at 28.5 °C. To image, all embryos were dechorionated and anaesthetized with 0.1% tricaine mesylate. For analysis of transient transgenic zebrafish, single embryos were transferred into a flat bottom 96-well plate, and GFP reporter gene expression was screened with a Zeiss LSM 710 confocal microscope at 46–50 hpf. The total number of injected fish, fish with detectable GFP expression and fish with GFP expression in the vasculature were noted. Whole fish were imaged using the tile scan command and combined with *Z*-stack collection under a confocal microscope Zeiss LSM 710 MP (Carl Zeiss) at 488 nm excitation and 509 nm emission (EGFP) and 587 nm and 610 nm (mCherry), respectively. For imaging of stable zebrafish transgenic lines (in Figs. 4 and 8), zebrafish embryos were treated with 0.03 mg/ml PTU (*N*-phenylthiourea, P7629, Sigma) at 24 hpf to prevent melanogenesis. Embryos were embedded in 0.4% TopVision low melting point agarose (R0801, Thermo) in 0.14 mg/ml Tricaine (ethyl 3-aminobenzoate methanesulfonat, A5040, Sigma) in glass bottom multi-well culture plates (MatTek).

Analysis of transgene expression was qualitative not quantitative, therefore no statistical analysis was applied to the observations of reporter gene intensity and pattern. Numbers of transgenic zebrafish embryos used followed precedent set by similar published papers, and was no <40/transgene. Where variations in expression were detected within a single experimental set, this is represented in the relevant figure by a graph summarizing variance with *n* numbers provided in the legend. No experimental randomization or blinding was used as we did not consider this necessary.

### MO and chemical inhibition in zebrafish

MOs were dissolved in ultrapure water and injected into 1–2-cell stage zebrafish embryos as previously described^19^. Sequences are provided in Supplementary Table 1; concentrations used were: *smad1* MO 0.5 ng; *smad5* MO 0.25 ng; alk1 MO; alk2 MO 3 ng; alk3a MO 5–7 ng; alk3b MO 1.2–1.8 ng; bmpr2a MO 12 ng; bmpr2b MO 6 ng; dab2 MO 2.5 ng; tnnt2 MO 4 ng.

For pharmacological inhibition of pathways downstream of VEGF, Notch and BMP, embryos were manually dechorionated and incubated with 100 µM DAPM (Calbiochem) added at 10 hpf^19^, 40 µM LY294002 (MedChem Express) added at 10 hpf^81^, 15–40 µM SL327 (SellekChem) added at 10 hpf^6^ or 5 µM DMH1 (Calbiochem) added at 10 hpf^18^. Control embryos were treated with identical concentrations of dimethyl sulphoxide without inhibitor.

All MO injections and use of chemical inhibitors were conducted at least three separate times. Analysis was qualitative not quantitative, therefore no statistical analysis was applied to the observations of staining intensity and pattern. Numbers of zebrafish embryos was no <40/tg (transgene) line. Where variations in expression were detected within a single experimental set, this is represented in the relevant figure by a graph summarizing variance with *n* numbers provided in the legend. No experimental randomization was used as we did not consider this necessary. Experimental blinding was not used as phenotypes of control and treated embryos were easily detectable due to dorsalization and sprouting defects.

### In situ hybridization

For zebrafish whole-mount in situ hybridization, *ephb4*, *efnb2*, *alk2*, *alk3a* and *alk3b* probes were generated as custom-made, double-stranded linear DNA fragments (GeneArt® Strings™, Life Technologies), cloned into the pCR2 vector using the TOPO/TA Cloning Kit (Invitrogen 450641) and transcribed using SP6 and T7. The sequences are provided below. *dll4*, *kdrl*, *bmp2b* and *bmp4* probes were used as previously described^70,72,73^. Whole-mount in situ hybridization was conducted as previously described^74^. Briefly, embryos were collected at 28 and 36 hpf, fixed overnight at 4 °C in 4% PFA, dehydrated and stored at −20 °C in 100% ethanol. Before use, embryos were rehydrated in 1×PBS with 0.1% Tween-20 (PBST), bleached in 3% H_2_O_2_/0.5% KOH and then embryos were re-fixed in 4% PFA for 20 min. The embryos were made permeable by digestion with 15 μg/ml proteinase K (Sigma-Aldrich) for 10 min (28 hpf embryos) or 30 min (36 hpf embryos) followed by two PBST washes, fixed in 4% PFA for 20 min, washed five times with PBST, then transferred into hybridization solution (50% formamide, 5× Saline-Sodium Citrate (SSC), 0.1% Tween 20, 50 μg/ml heparin, 500 μg/ml yeast tRNA, 10 mM citric acid) for 2 h at 65 °C, transferred into diluted antisense riboprobe/hybridization solution and incubated overnight at 65 °C. Probes were removed and embryos relocated to a Biolane HT1 in situ machine (Intavis). Embryos were washed through a dilution series of 2× SSC followed by 0.2× SSC at 65 °C and thereafter taken through room temperature dilution washes of 100% MABT (0.1 M maleic acid, 0.15 M NaCl, pH 7.5). Nonspecific sites were blocked with MAB block (MABT with 2% Boehringer block reagent), and the embryos were incubated for 15 h with 1:2000 antiDIG antibody (Roche) at 4 °C, before washing in MABT. Prior to staining, embryos were washed in alkaline phosphatase (AP) buffer and the in situ signal was developed at room temperature with BM Purple (Sigma-Aldrich). Staining was stopped as appropriate by fixation in 4% PFA. Embryos were transferred to 80% glycerol for imaging and storage.

All in situ analyses were conducted at least two separate times. Analysis was qualitative not quantitative, therefore no statistical analysis was applied to the observations of staining intensity and pattern. Numbers of zebrafish embryos were <20/in situ condition. Where variations in expression were detected within a single experimental set, this is represented in the relevant figure by a graph summarizing variance with *n* numbers provided in the legend and by the numbers next to the representative pictures. No experimental randomization was used as we did not consider this necessary. Experimental blinding was not used as phenotypes of control and treated were easily detectable due to dorsalization and sprouting defects. Sequences of each probe are provided in the Supplementary Table 1.

For mouse in situ hybridization, *Bmp4* sense and antisense RNA probes (from Allen Brain Atlas Data Portal^72^) were prepared as for the zebrafish. Wild-type E9.5 and E10.5 embryos were harvested, fixed overnight in 4% PFA/PBS at 4 °C, and paraffin embedded as described above. Ten-µm sections were cut, de-waxed using Histoclear and rehydrated through an ethanol series. The sections were then digested in 20 µg/ml Proteinase K (Sigma-Aldrich) for 8 min, followed by washes in 2 mg/ml Glycine/PBS and then PBS. Sections were fixed for 20 min in 4% PFA/PBS, washed twice in PBS and then incubated in a humidified chamber for 1 h at 70 °C in Hybridization buffer (50% formamide, 5× SSC buffer pH 4.5, 50 µg/ml yeast RNA, 1% sodium dodecyl sulphate (SDS), 50 µg/ml heparin). This was followed by an overnight incubation in Hybridization buffer containing 1 µg/ml sense or antisense RNA probe at 70 °C. The following morning, slides were rinsed twice in 2× SSC buffer and then at 65 °C underwent three 15-min washes in Solution I (50% formamide, 5× SSC pH4.5, 1% SDS) and two 15-min washes in Solution II (50% formamide, 2× SSC pH4.5). They were then returned to room temperature for two washes in MABT buffer (0.1 M maleic acid, 0.15 M NaCl, 0.01% Tween-20, 2 mM Levamisole (Sigma-Aldrich), pH7.5) before blocking for 1 h in 2% Boehringer Blocking Reagent (Roche)/10% sheep serum/MABT. They were then incubated overnight at 4 °C in the blocking solution containing AP-conjugated anti-DIG antibody (Roche) diluted 1:2000. Finally slides were washed three times in MABT and then two times in AP buffer (100 mM Tris, pH 9.5, 50 mM MgCl_2_, 100 mM NaCl, 0.1% Tween-20, 2 mM Levamisol), before AP activity was detected using BM Purple (Roche).

### Immunostaining

For whole-mount DLL4 and CD31 staining, embryos were dissected and fixed in 4% PFA on ice for 1 h (DLL4) or overnight (CD31), rinsed in PBST (0.1% TritonX-100 in PBS), incubated for 1 h in blocking solution (10% Normal Donkey Serum in PBST), then overnight at 4 °C with goat polyclonal to DLL4 (R&D systems, AF1389,1 in 50 dilution) or rat monoclonal to CD31 (DIA-310, Dianova, 1 in 250 dilution). Samples were washed in PBST and subsequently incubated overnight with suitable species-specific Alexa Fluor® or horseradish peroxidase-conjugated secondary antibodies (1:300, Thermo Fisher Scientific) in 0.1% PBST at 4 °C.

For immunofluorescence staining on paraffin sections, E8.5–E10.5 mouse embryos were harvested and fixed overnight in 4% PFA/PBS at 4 °C, embedded and sectioned as described above. Sections were de-waxed by two washes in Histoclear and rehydrated through an ethanol series. Antigen retrieval was carried out by boiling slides in 10 mM sodium citrate buffer, pH 6.0, in a commercial pressure cooker for 3 min, followed by two washes in PBS. Sections were then incubated at room temperature in blocking solution (1% bovine serum albumin (BSA), 2% donkey serum in PBS or 10% donkey serum in PBS) for 1 h in a humidified chamber, followed by overnight incubation at 4 °C in primary antibodies diluted in blocking solution. Sections were rinsed in PBS and incubated in species-specific Alexa Fluor®-conjugated secondary antibodies diluted 1:1000 in blocking solution for 1 h at room temperature. Finally, sections were rinsed further in PBS, stained with 4,6-diamidino-2-phenylindole (DAPI) and mounted with glass coverslips using Fluoromount^TM^ Aqueous Mounting Medium (Sigma), before imaging on a Zeiss LSM 710 Confocal Laser Scanning Microscope. Primary antibodies used were rat monoclonal to CD31 (DIA-310, Dianova) diluted 1:300, rabbit monoclonal to Neuropilin 1 (ab81321, Abcam) diluted 1:100, rabbit polyclonal to ALK3 (ab38560, Abcam) diluted 1:100 and goat polyclonal to EPHB4 (AF446, R&D Systems) diluted 1:50.

For COUP-TFII immunohistochemical staining on paraffin sections, E10.5 mouse embryos were processed the same as for the immunofluorescence staining up to the antigen retrieval step. Following antigen retrieval, sections were washed in PBS and then incubated in 3% hydrogen peroxide (Sigma Aldrich) for 5 min and rinsed again in PBS. The Avidin/Biotin Blocking Kit (SP-2001, Vector Laboratories) and Mouse on Mouse (M.O.M.™) Basic Kit (BMK-2202, Vector Laboratories) were then used according to the manufacturer’s instructions, with a mouse monoclonal antibody to COUP-TFII (PP-H7147–00, Perseus Proteomics Inc) diluted 1:100 in the M.O.M.^TM^ diluent. Following incubation in the M.O.M.™ Biotinylated Anti-Mouse IgG reagent, sections were incubated for 10 min in VECTASTAIN® Elite® ABC Reagent (from PK-6102, Vector Laboratories), rinsed in PBS and then incubated in DAB substrate (SK-4100, Vector Laboratories), including Nickel solution, until colour developed. Slides were imaged using a NanoZoomer S210 slide scanner with the NDP.view2 viewing software (Hamamatsu).

For immunofluorescence staining on cryosections, E9.5 mouse embryos were harvested and fixed in 4% PFA/PBS at 4 °C for 1 h, before incubation in 30% sucrose/PBS overnight at 4 °C. They were then washed in a 50/50 mix of 30% sucrose/OCT Embedding Medium (Thermo Scientific), two washes in OCT and then mounted over dry ice and stored at −80 °C. Cryosections were cut at a thickness of 12 μm, thawed at room temperature and washed in PBS to remove the OCT embedding medium. They were fixed in 4% PFA/PBS for 10 min, washed three times in PBS and permeabilized in 0.5% Triton X-100/PBS (Merck) for 12 min. Following two further PBS washes, sections were blocked in 10% donkey serum/0.1% Triton X-100/PBS for 1 h in a humidified chamber at room temperature, before overnight incubation at 4 °C in primary antibodies diluted in the blocking solution. Following further PBS washes, sections were incubated at room temperature in Alexa Fluor®-conjugated secondary antibodies diluted 1:1000 in blocking solution for 1 h. Sections were then DAPI-stained, mounted and imaged as described above. Primary antibodies used were rabbit monoclonal to Phospho-SMAD1 (Ser463/465)/ SMAD5 (Ser463/465)/ SMAD9 (Ser465/467) (D5B10, Cell Signaling Technology), goat polyclonal to EPHB4 (AF446, R&D Systems) diluted 1:50 and rat monoclonal to CD31 (DIA-310, Dianova) diluted 1:300.

### ClustalW and sequence motif analysis

Mouse and human sequences of putative enhancers were aligned using ClustalW^76^. Binding motifs for the vascular ETS factors ERG and ETV2 and SMAD4 were obtained from JASPAR and annotated by hand.

### Bioinformatic analysis of SMAD1/5-enriched binding sites

SMAD1/5-binding information was obtained using publically available ChIP-seq data^32^ via the NCBI GEO database^77^, accession number GSE27661. Raw reads for HUVEC stimulated by BMP9 and PASMC stimulated by BMP4 sets were trimmed with Sickle. Reads were then aligned to human genome build hg19 using Bowtie2, duplicate PCR reads removed with rmdup and peaks were called with MACS2. A bedgraph was generated of the significant peaks for visualization and comparison to other genomic data sets.

ERG-binding information from HUVEC was obtained using publically available ChIP-seq data^29^ via ArrayExpress^73^, accession number E-MTAB-5148. Raw reads were trimmed with Sickle, aligned to human genome build hg19 using Bowtie2, duplicate PCR reads removed with rmdup and peaks were called with MACS2. A bedgraph was generated of the significant peaks for visualization and comparison to other genomic data sets.

### Chromatin immunoprecipitation

HUVECs (PromoCell, C-12203) were grown in Endothelial Cell Growth Medium 2 with the BulletKit (PromoCell). Media was changed every 48 h. Four 80% confluent 15-cm dishes per condition were serum starved in 0.5% Foetal Bovine Serum (Gibco) overnight before being stimulated with BMP9 1 ng/ml for 1.5 h. Cells were then trypsinized and the cell pellet collected. Crosslinking was performed in 0.6% methanol-free formaldehyde (Pierce) under rotation at room temperature for 12’ before being quenched with glycine to a concentration of 0.2 M. Lysis was achieved by passing cell suspension through a 25-g needle (Terumo #NN-2525R) in 1 ml of cell lysis buffer (50 mM Tris-HCL(pH8.0), 10 mM EDTA, 10 mM Sodium butyrate, 1% SDS, 0.5 mM phenylmethanesulfonylfluoride and cOmplete, EDTA-free protease inhibitor cocktail (Roche)).

To obtain a mean chromatin fragment size of 650–850 bp chromatin was sheared by sonication using a Covaris sonicator S220 in a Covaris AFA fiber tube at 160 W peak incidence power, 5% duty cycle, 200 cycles, for 8’. Fragment size was checked by agarose gel. Sonicated chromatin was centrifuged at 14,000 × *g*, 10’, and the supernatant was diluted in 8 ml ChIP dilution buffer (16.7 mM Tris (pH8.0), 167 mM NaCl, 1.2 mM EDTA, 1% Triton X-100, 0.01% SDS) and incubated overnight with 8 µg of antibody, Smad1 (Iwai North America BMR00479) or IgG control (12–371 merckmillipore) and a no antibody control. IP was performed with Dynabeads-protien G (ThermoFischer) and blocked overnight in 0.5 mg/ml BSA (Sigma-Aldrich) under rotation for 1 h. G-Dynabead immunocomplexes were washed with low-salt buffer (20 mM Tris-HCL (pH8.0) 150 mM NaCl, 2 mM EDTA, 1% Triton X-100, 0.1% SDS), high-salt buffer (20 mM Tris-HCL (pH8.0) 500 mM NaCl, 2 mM EDTA, 1% Triton X-100, 0.1% SDS) and LiCl buffer (250 mM LiCl, 0.5% NP-40, 0.5% sodium deoxycholate, 1 mM EDTA, Tris–HCL 10 mM, pH 8.0). Beads were eluted in 0.2 ml elution buffer and ChIPed-DNA was reverse crosslinked overnight at 55 °C in elution buffer plus 0.3 M NaCL, 20 µg RNase A (Invitrogen) and 20 µg Proteinase K (Fermentas). DNA was column purified with the QIAquick PCR Purification Kit (Qiagen).

Immunoprecipitated DNA was analysed by qPCR using TaqMan Custom Gene Expression Assay Probes (ThermoFischer) designed against 100 bp regions of the Ephb4-2 enhancer or a gene dessert region of Chromosome 7 as a negative control; sequences are detailed in Supplementary Methods.

Each ChIP was performed on at least three biological replicates, with three technical replicates for each. Statistical analysis was performed in the StepOne plus software, Microsoft Excel. Input was taken as the supernatant from the non-antibody control condition. qPCR analysis of 1% input was run alongside SMAD1 antibody analysis for each region on each qPCR. Results are expressed as the mean of the percentage of input defined as 100 × (2^(adjusted Input ct − ct IP)) across all replicates. Significance was calculated with a paired two-tailed *t* test comparing percentage of input of control region to percentage of input of SMAD1 antibody conditions. Graphs were produced using R statistical package.

### Reporting Summary

Further information on experimental design is available in the Nature Research Reporting Summary linked to this article.

## Supplementary information

Supplementary Information

Reporting Summary

## Data Availability

The authors declare that the main data supporting the findings of this study are available within the article, its Supplementary Figures and Methods. All ChIP-seq datasets used in this study were previously published and are publicly available; references and accession numbers are provided within the article. Extra data are available from the corresponding author upon request. A reporting summary of this article is available as a Supplementary Information file.
