# Supplementary material for: Venous identity requires BMP signalling through ALK3
Source: Nat Commun. 2019 Jan 28;10:453. doi: 10.1038/s41467-019-08315-w (PMC6349860; doi:10.1038/s41467-019-08315-w)
Supplement: Supplementary file 1 — Supplementary Information [file 41467_2019_8315_MOESM1_ESM.pdf]

Supplementary Information

Venous Identity Requires BMP Signalling Through ALK3

Neal et al.

Supplementary Methods

Enhancer sequences

For venous enhancers, all wild type and mutant enhancer sequences were initially generated as custom-made, double-stranded linear DNA fragments (GeneArt® Strings™, Life Technologies) with the exception of Coup-TFII-965 and Nrp2+26, which were generated by PCR from genomic DNA.

Ephb4-2 WT enhancer (mouse)

AATCAGTGCGTGCTCGTTAAGTCCTGGAGATCCACTGAGCGCGCAGCCTAACGCTGGAGAAA
GTGGTTTGAAACCCAAAGTATAGAAAATGTAAATAAAAGGCAGGCGTGTCAGAGAGGGTGAG
GGATCTCCGTAACACCTCATTTCATTTTTTTAAAGGAGGGGGACACTTCCCCGCCGCCTGCA
GCCTTGACCTCCAAGGCGGGGGTAGGGACCGTTGTGGCTCTTTCCTGAGGCTGTTTCCTGTC
TGGCTCCTGGGGGCCCTCGGGATGGCTGGGAGGGCCCTTCCTCTCATTTGCTAGCACCCCCT
CTCATCCATCAGTTTGAGGGGAGGGTCCAGGAAAGACGGCCTCCTATCTACATCAGGGCACT
GTGAGTGTGGGGCACGGGATGGTTGGATGAGAGAGGTGCTGTTCCCGAAGTCGGTCCTTTAA
GGGCTGCGGTAAGGAGACTTTAATTTAAGGTAATTAGTACAGGGTCTGGAAACTCTGAGGTA
GGAGTCTGGGGCACCTGGGAGTCTGCCAAATACCCTAAGGGCGCACACACACACCCCAGCGG
GCGACCGGTGATGACCTCTTGTCCGCCTGCGCGCACACACACACCAGCGGGCGCGGGAGACC
CGTGATGGCCTTTTGTCCCCGTGCACTTATCTTCCTGGCGCAAGTAGTGCTCCCCACCCCCT
GCCCTTCCTCACAGCCCTGCCTGGGTCCCGCTCCGGGGTGGGTCAGCCAGGGCAGGAAACAG
CCGGCTTGGCTGGAGCCAGGCTGACCGGCTAGATCTGGGAGTCCCCTCCTCCTTCCCCACGC
AGACTCAGGCTCCCCTTCTCTTATCCACAGACACCCCCTTTTTTGCAGCTATCATTCTGCAT
CCGGGTCCCCCTGAATTTCTGAGTCGTGGCTTGTTCTCAC

Ephb4-2mutSBE(all)

AATCAGTGCGTGCTCGTTAAGTCCTGGAGATCCACTGAGCGCGCAGCCTAACGCTGGAGAAA
GTGGTTTGAAACCCAAAGTATAGAAAATGTAAATAAAAGGCAGGCGTGTCAGAGAGGGTGAG
GGATCTCCGTAACACCTCATTTCATTTTTTTAAAGGAGGGGGACACTTCCCCGCCGCCTGCA
agCTTGACCTCCAAGGCGGGGGTAGGGACCGTTGTGGCTCTTTCCTGAGGCTGTTTCCTGTC
caGCTCCTGGGGGCCCTCGGGATGctTGGGAGGGCCCTTCCTCTCATTTGCTAGCACCCCCT
CTCATCCATCAGTTTGAGGGGAGGGTCCAGGAAAGACGGCCTCCTATCTACATCAGGGCACT
GTGAGTGTGGGGCACGGGATGGTTGGATGAGAGAGGTGCTGTTCCCGAAGTCGGTCCTTTAA
GGGCTGCGGTAAGGAGACTTTAATTTAAGGTAATTAGTACAGGGctTGGAAACTCTGAGGTA
GGAGTCTGGGGCACCTGGGAGTCTGCCAAATACCCTAAGGGCGCACACACACACCCCAGCGG
GCGACCGGTGATGACCTCTTGTCCGCCTGCGCGCACACACACACCAGCGGGCGCGGGAGACC
CGTGATGGCCTTTTGTCCCCGTGCACTTATCTTCCTGGCGCAAGTAGTGCTCCCCACCCCCT
GCCCTTCCTCACAGCCCTGCCTGGGTCCCGCTCCGGGGTGGGTCAGCCAGGGCAGGAAACAa
gCGGCTTGGCTGGAagCAGGCTGACCGctTAGATCTGGGAGTCCCCTCCTCCTTCCCCACGC
AGACTCAGGCTCCCCTTCTCTTATCCACAGACACCCCCTTTTTTGCAGCTATCATTCTGCAT
CCGGGTCCCCCTGAATTTCTGAGTCGTGGCTTGTTCTCAC

Ephb4-2mutSBE(2/3,6/7)

AATCAGTGCGTGCTCGTTAAGTCCTGGAGATCCACTGAGCGCGCAGCCTAACGCTGGAGAAA
GTGGTTTGAAACCCAAAGTATAGAAAATGTAAATAAAAGGCAGGCGTGTCAGAGAGGGTGAG
GGATCTCCGTAACACCTCATTTCATTTTTTTAAAGGAGGGGGACACTTCCCCGCCGCCTGCA
GCCTTGACCTCCAAGGCGGGGGTAGGGACCGTTGTGGCTCTTTCCTGAGGCTGTTTCCTGTC
caGCTCCTGGGGGCCCTCGGGATGGCTGGGAGGGCCCTTCCTCTCATTTGCTAGCACCCCCT
CTCATCCATCAGTTTGAGGGGAGGGTCCAGGAAAGACGGCCTCCTATCTACATCAGGGCACT
GTGAGTGTGGGGCACGGGATGGTTGGATGAGAGAGGTGCTGTTCCCGAAGTCGGTCCTTTAA
GGGCTGCGGTAAGGAGACTTTAATTTAAGGTAATTAGTACAGGGTCTGGAAACTCTGAGGTA
GGAGTCTGGGGCACCTGGGAGTCTGCCAAATACCCTAAGGGCGCACACACACACCCCAGCGG

GCGACCGGTGATGACCTCTTGTCCGCCTGCGCGCACACACACACCAGCGGGCGCGGGAGACC
CGTGATGGCCTTTTGTCCCCGTGCACTTATCTTCCTGGCGCAAGTAGTGCTCCCCACCCCCT
GCCCTTCCTCACAGCCCTGCCTGGGTCCCGCTCCGGGGTGGGTCAGCCAGGGCAGGAAACAa
gCGGCTTGGCTGGAGCCAGGCTGACCGGCTAGATCTGGGAGTCCCCTCCTCCTTCCCCACGC
AGACTCAGGCTCCCCTTCTCTTATCCACAGACACCCCCTTTTTTGCAGCTATCATTCTGCAT
CCGGGTCCCCCTGAATTTCTGAGTCGTGGCTTGTTCTCAC

Ephb4-2mutSBE(2/3)

AATCAGTGCGTGCTCGTTAAGTCCTGGAGATCCACTGAGCGCGCAGCCTAACGCTGGAGAAA
GTGGTTTGAAACCCAAAGTATAGAAAATGTAAATAAAAGGCAGGCGTGTCAGAGAGGGTGAG
GGATCTCCGTAACACCTCATTTCATTTTTTTAAAGGAGGGGGACACTTCCCCGCCGCCTGCA
GCCTTGACCTCCAAGGCGGGGGTAGGGACCGTTGTGGCTCTTTCCTGAGGCTGTTTCCTGTC
caGCTCCTGGGGGCCCTCGGGATGGCTGGGAGGGCCCTTCCTCTCATTTGCTAGCACCCCCT
CTCATCCATCAGTTTGAGGGGAGGGTCCAGGAAAGACGGCCTCCTATCTACATCAGGGCACT
GTGAGTGTGGGGCACGGGATGGTTGGATGAGAGAGGTGCTGTTCCCGAAGTCGGTCCTTTAA
GGGCTGCGGTAAGGAGACTTTAATTTAAGGTAATTAGTACAGGGTCTGGAAACTCTGAGGTA
GGAGTCTGGGGCACCTGGGAGTCTGCCAAATACCCTAAGGGCGCACACACACACCCCAGCGG
GCGACCGGTGATGACCTCTTGTCCGCCTGCGCGCACACACACACCAGCGGGCGCGGGAGACC
CGTGATGGCCTTTTGTCCCCGTGCACTTATCTTCCTGGCGCAAGTAGTGCTCCCCACCCCCT
GCCCTTCCTCACAGCCCTGCCTGGGTCCCGCTCCGGGGTGGGTCAGCCAGGGCAGGAAACAG
CCGGCTTGGCTGGAGCCAGGCTGACCGGCTAGATCTGGGAGTCCCCTCCTCCTTCCCCACGC
AGACTCAGGCTCCCCTTCTCTTATCCACAGACACCCCCTTTTTTGCAGCTATCATTCTGCAT
CCGGGTCCCCCTGAATTTCTGAGTCGTGGCTTGTTCTCAC

Ephb4-2mutSBE(6/7)

AATCAGTGCGTGCTCGTTAAGTCCTGGAGATCCACTGAGCGCGCAGCCTAACGCTGGAGAAA
GTGGTTTGAAACCCAAAGTATAGAAAATGTAAATAAAAGGCAGGCGTGTCAGAGAGGGTGAG
GGATCTCCGTAACACCTCATTTCATTTTTTTAAAGGAGGGGGACACTTCCCCGCCGCCTGCA
GCCTTGACCTCCAAGGCGGGGGTAGGGACCGTTGTGGCTCTTTCCTGAGGCTGTTTCCTGTC
TGGCTCCTGGGGGCCCTCGGGATGGCTGGGAGGGCCCTTCCTCTCATTTGCTAGCACCCCCT
CTCATCCATCAGTTTGAGGGGAGGGTCCAGGAAAGACGGCCTCCTATCTACATCAGGGCACT
GTGAGTGTGGGGCACGGGATGGTTGGATGAGAGAGGTGCTGTTCCCGAAGTCGGTCCTTTAA
GGGCTGCGGTAAGGAGACTTTAATTTAAGGTAATTAGTACAGGGTCTGGAAACTCTGAGGTA
GGAGTCTGGGGCACCTGGGAGTCTGCCAAATACCCTAAGGGCGCACACACACACCCCAGCGG
GCGACCGGTGATGACCTCTTGTCCGCCTGCGCGCACACACACACCAGCGGGCGCGGGAGACC
CGTGATGGCCTTTTGTCCCCGTGCACTTATCTTCCTGGCGCAAGTAGTGCTCCCCACCCCCT
GCCCTTCCTCACAGCCCTGCCTGGGTCCCGCTCCGGGGTGGGTCAGCCAGGGCAGGAAACAa
gCGGCTTGGCTGGAGCCAGGCTGACCGGCTAGATCTGGGAGTCCCCTCCTCCTTCCCCACGC
AGACTCAGGCTCCCCTTCTCTTATCCACAGACACCCCCTTTTTTGCAGCTATCATTCTGCAT
CCGGGTCCCCCTGAATTTCTGAGTCGTGGCTTGTTCTCAC

Ephb4-2mutEBE(all)

AATCAGTGCGTGCTCGTTAAGTCCTGGAGATCCACTGAGCGCGCAGCCTAACGCTGGAGAAA
GTGGTTTGAAACCCAAAGTATAGAAAATGTAAATAAAAGGCAGGCGTGTCAGAGAGGGTGAG
GGATCTCCGTAACACCTCATTTCATTTTTTTAAAGGAGGGGGACACtAGcCCGCCGCCTGCA
GCCTTGACCTCCAAGGCGGGGGTAGGGACCGTTGTGGCTCttAGctGAGGCTGttAGctGTC
TGGCTCCTGGGGGCCCTCGgAGtGGCTGGGAGGGCCCTAGCTCTCATTTGCTAGCACCCCCT
CTCATCCATCAGTTTGAGGGGAGGGTCCAGGAAAGACGGCCTCCTATCTACATCAGGGCACT
GTGAGTGTGGGGCACGgTCtGGTTgTCtGAGAGAGGTGCTGTTCCCGAAGTCGGTCCTTTAA
GGGCTGCGGTAAGGAGACTTTAATTTAAGGTAATTAGTACAGGGTCTGGAAACTCTGAGGTA
GGAGTCTGGGGCACCTGGGAGTCTGCCAAATACCCTAAGGGCGCACACACACACCCCAGCGG
GCGACCGGTGATGACCTCTTGTCCGCCTGCGCGCACACACACACCAGCGGGCGCGGGAGACC
CGTGATGGCCTTTTGTCCCCGTGCACTTATctTCctGGCGCAAGTAGTGCTCCCCACCCCCT
GCCctTCctCACAGCCCTGCCTGGGTCCCGCTCCGGGGTGGGTCAGCCAGGGCagTCaaCAG
CCGGCTTGGCTGGAGCCAGGCTGACCGGCTAGATCTGGGAGTCCCCTCCTCCTTCCCCACGC

AGACTCAGGCTCCCCTTCTCTTATCCACAGACACCCCCTTTTTTGCAGCTATCATTCTGCAT
CCGGGTCCCCCTGAATTTCTGAGTCGTGGCTTGTTCTCAC

Ephb4-4mutEBE(2,3,5,8,9,10)

AATCAGTGCGTGCTCGTTAAGTCCTGGAGATCCACTGAGCGCGCAGCCTAACGCTGGAGAAA
GTGGTTTGAAACCCAAAGTATAGAAAATGTAAATAAAAGGCAGGCGTGTCAGAGAGGGTGAG
GGATCTCCGTAACACCTCATTTCATTTTTTTAAAGGAGGGGGACACTTCCCCGCCGCCTGCA
GCCTTGACCTCCAAGGCGGGGGTAGGGACCGTTGTGGCTCttAGctGAGGCTGttAGctGTC
TGGCTCCTGGGGGCCCTCGGGATGGCTGGGAGGGCCctAGctCTCATTTGCTAGCACCCCCT
CTCATCCATCAGTTTGAGGGGAGGGTCCAGGAAAGACGGCCTCCTATCTACATCAGGGCACT
GTGAGTGTGGGGCACGGGATGGTTGGATGAGAGAGGTGCTGTTCCCGAAGTCGGTCCTTTAA
GGGCTGCGGTAAGGAGACTTTAATTTAAGGTAATTAGTACAGGGTCTGGAAACTCTGAGGTA
GGAGTCTGGGGCACCTGGGAGTCTGCCAAATACCCTAAGGGCGCACACACACACCCCAGCGG
GCGACCGGTGATGACCTCTTGTCCGCCTGCGCGCACACACACACCAGCGGGCGCGGGAGACC
CGTGATGGCCTTTTGTCCCCGTGCACTTATctAGctGGCGCAAGTAGTGCTCCCCACCCCCT
GCCctAGctCACAGCCCTGCCTGGGTCCCGCTCCGGGGTGGGTCAGCCAGGGCagTCaaCAG
CCGGCTTGGCTGGAGCCAGGCTGACCGGCTAGATCTGGGAGTCCCCTCCTCCTTCCCCACGC
AGACTCAGGCTCCCCTTCTCTTATCCACAGACACCCCCTTTTTTGCAGCTATCATTCTGCAT
CCGGGTCCCCCTGAATTTCTGAGTCGTGGCTTGTTCTCAC

Ephb4-4mutCONT

AATCAGTGCGTGCTCGTTAAGTCCTGGAGATCCACTGAGCGCGCAGCCTAACGCTGGAGAAA
GTGGTTTGAAACCCAAAGTATAGAAAATGTAAATAAAAGGCAtaCGTGTCAGAGAGGGTGAG
GGATCTCCGTAACACCTCATTTCATTTTTTTAAAGGAGGGGGACACTTCCCCGCCGCCTGCA
GCCTTGACCTCCAAGGCGGGGGTAGGGACCGTTGTGGCTCTTTCCTGAGGCTGTTTCCTGTC
TGGCTCCTGGGGGCCgaCGGGATGGCTGGGAGGGCCCTTCCTCTCATTTGCTAGCACCCCCT
CTCATCCAgaAGTTTGAGGGGAGGGTCCAGGAAAGACGGCCTCCTATCTACATCAGGGCACT
GTGAGTGTGGGGCACGGGATGGTTGGATGAGAGAGGTGCTGTTCCCGAAGTCGGTCCTTTAA
GGGCTGCGGTAAGGAGACTTTAATTTAAGGTAATTAGTACAGGGTCTGGAAACTCTGAGGTA
GGAGTCTGGGGCACCTGGGAGTCTGCCAAATACCCTAAGGGCGCACACACACACCCCAGCGG
GcgACCGGTGATGACCTCTTGTCCGCCTGCGCGCACACACACACCAGCGGGCGCGGGAGACC
CGTGATGGCCTTTTGTCCCCGTGCACTTATCTTCCTGGCGCAAGTAGTGCTcgCCACCCCCT
GCCCTTCCTCACAGCCCTtaCTGGGTCCCGCTCCGGGGTGGgtCAGCCAGGGCAGGAAACAG
CCGGCTTGGCTGGAGCCAGGCTGACCGGCTAGATCTGGGAGTCCCCTCCTCCTTCCCCACGC
AGACTCAGGCTCCCCTTCTCTTATCCACAGACACCCCCTTTTTTGCAGCTATCAggCTGCAT
CCGGGTCCCCCTGAATTTCTGAGTCGTGGCTTGTTCTCAC

Ephb4-10 enhancer (mouse)

TATGCAGCCTACGCTAATACTTGCCTGTCACTGTTGAAAGGGGCTGGTTCACTGCCTACCAC
TGCAGAAAAGCCTATCCACCTTGCCTTCAACCCTGTCAGGGAGAAGAAGGACCGAGGTTGCT
GTTGGGATATCAGAGAGTAGAGAGGACCTATTGGAACCTGATGGAGAACCGGATAAGGCAGA
GCTAGCTGGTATGTGCGTCTGTGTGTGTGCGTGCGTGCGTGCGTGTGTGCGCCGCGTGTGCA
TGCATGTGCGTGAGTGTGTGGCGGGGCACAGTGTAGAAGGGAGGCTGAGAGACTGGGGCCAG
AGTTTAAGGTGGCAGGGGATTGGAAAGGTGGAGCAAAGGGAGGGTGGAGGAGAGTTAGTGGG
AGGCTCATTGTCCTGGTGGGGGGAAGTCTTTTGTTTCCTCTCTTCCCTTGGCAGCCTGTCAG
GATGTGAGGCCAAGCCCCCCTCCCATGCCAATATCTCCGGCCCCAACCATCAGACTGCTCTT
TCCTTTACTATTGTGTTCCTGATGGGAAAGGGGGCGGGAGCAGGGGCCGGCTGAAGACTAAC
TCTGGTTGGGACTCTGAGAACTATTAGTGGTTAGGGGCTAGGTTACCGGGGAGTTCTAAAAG
GACCTAAAGAATCAGAGTCGGCTAGGTCAGCTTGGCCGGGTAAGATCTGTGTTGGGGAGGAG
CAGGAAGCCAGGCCCCGGGTGGGGCCACCACAGAGGGTAGCAGGTCGGGCCAGGGGGGAGGT
AGGCTGGGTCCCAGAGGAGATGACGCAGGGCTTCAGGCGTCTCCCAGCCCCCACTATTCTCG
CTTGGGAGCCAAACAGCACACCTGAACCCCAAGATTGCCCCCACCCCCTCCATTCCCTGAGG
TTGACATCACCCCACCAAGCTTGCAAATTCAGGTGTCCCCGGGCCATCTAAGGTCCAGGTCC
CTTGATGCTCTCACTCCTCTCCAGGATCTAGTAGGATCCATGGAGGAACCCTGGAATGCCAT
TCCTCCCCTCTGCTCCCTCCTGGATCTTTTTTTTTTTTTTTTTTTTTTTCTCCATCAGCT
GTCCTGCAGGAAGGGCGCCTTGCTCAGCTTTGCCTCCCTTCCCGAGCCTTTTGTTTGCTGGA
TTTTCAGGAAATTGGAGGGGTGGTGGCCAAGGGCTCATCTGCCCTAGGGACCTGGACATGGT

TGTGGTGGAGGGGGTAGGGACAGAATCACCAGACTCTGGGGAACTCACGGTGGGAGGGAGGG
AGGATTGGGGGCATCGCCAGGAAATTATTATATTATCATATGAGGTCTCGTTCGGAGTTCCC
GGGTTGATTGGACTTCCTGATCGGACAAGGAAAGGACAGATTGTGTGTGTGTGTGTGTGTAT
GAGAGGCTGCGCTGACATGAGGTCACTTTAATAAGCTGGCAAAGAGTTGACACAAAGGGGGA
AGAACAGTTACTAAACGCCACAAGTTCATCCTCCTGCCTTGCATCCTGGACCCAAGAAGTG
CGGAGGTAGCGGGTAGTCAGATCATGCCACCACTGAAGTTGGGTGGTTGGGCTTTTTGTTTG
TTTGCTTGCTTGTTTCCCGTCTTTTAAAAAAAAAAAAAAAAAAAACTAGAGAGGAGAGGGCCG
TCCAGTGCGTAGAGCCAGAATTCCCGTCTTCCCAGCATACAACCGCAGAAACCACGCCGGTA
TGGA

Coup-TFII-965 enhancer long (mouse)

Primer F CATCCCACATCAGAACCTTGC
Primer R GCAAGCTGAATGGAAGGCTC
CATCCCACATCAGAACCTTGCAGAGCCCAAGCTCAGGCCTATCGGATCTAATTAGATAAAGA
CCCTTCATTGATGAGACTACTTTAAAATGAACAGTGATTTCAGCTTCAAAAGTGCATGCCTT
TCTTTTCTATATCTCAGACATAGAGCAAAGTCAATAGACCGACCCGCCCACAATGTGTTCTA
AAATAACCCTGAATTCAGATGCACTGAAAAATGGTTAAATGAACTGATAAAGCATAGGGAAT
AACATATAATATCCTGTAGAGATGTACGCCTCCTACATAGATGGATACTGAATATACCTCTA
AACATGTGTTATTTTAACAATCACATGCTGTAGCTCCCAATTTATTCTCATGAGGAGGGAAA
TAAACACAGATTTTCTTGACCGTAATTAATGTGTTTAGGGACCACACTCCACGTTTTACTGA
GTCCCTGTGCACTGGGCTCCTTCGGTCATCTCTCTTGAAGACAAAATGCACCTTTTTCTTAA
GTAGTCCCGAGGTTTGGTTTGTTTGTTTATTCAGGGGATGGTGGAAGGACATGGTTTCTCTG
GAAAGCTGGCTCCTGTGTTCTCAGGGGTTATAAAAATGGGAATCATTTTTATTGGCAATCCC
CGGGAATAGTGCTGAGACAAATGGAAAGCTGAAGATAAGGATCCTCTGAGGTGCGAACATAC
AGCTGTTGGGAATTGCCAGAGAATCGGACCAATAAAGGAAGTCACTATTTTTCCAGGCCTGA
AGTGAGTTATAGGGCGAGACGGGTGTTGTATATTTATGTAAGGCAACAGCAGGGAGTTTAAG
CGGCTGGATATTGCTGAAAGAGCATCATTCACATTCAGGCGGAGACAAAAGGTGGAAATGAA
GCAACATCCTGGCCAAAGAAGGCCTCAAGACAGAATAATAACAGTTCAGAGAGGGGGGCTGT
GTGCACGGCCGAGGGTCGGCCTCAAAACCAGGAAATGATCGAGATGCCTTGTCAGATCTTCA
GAAAACAGTCACACACACGCACACGTGCAGGGATACACATACACACTCAAACTCAGGCCTGG
CCAAGTGTCCTTGGGAAGGAGGTCATTTATCTTTCATAGAACTGTGGACAGAATTACCTGCA
ACCTTCATCAGTGTAGCCAACAAGATTGGCTTACCAAAAGTGCTGGAAAAAAATTGTATTTT
GTACTGTTGTATGTGTACACACATACATACAAAAAATTTTTATACACACATATGTATAAAAA
TATGACATCCACATGTGTCAGCAGGCCTCTTGTTTGCTATCTGTTTTACACAGACTTCAAAC
TCACTGAGTTAGAGAAAATTCTAATTCAGGAAAATATTCTAAGAACATAGATCTGGTTGTCC
TAGAGTTCCAAGGAAGGGGATGGGCAGGCAAAGAGACTGTCTTATCTTCTTGTTTATTTATT
GTCCTTTTGCTTAGAGGAAGTTGCAAGCTGAATGGAAGGCTC

CoupTFII-965 enhancer short

Primer F GCTGAGACAAATGGAAAGCTG
Primer R GAGATGCCTTGTCAGATCTTC
GCTGAGACAAATGGAAAGCTGAAGATAAGGATCCTCTGAGGTGCGAACATACAGCTGTTGGG
AATTGCCAGAGAATCGGACCAATAAAGGAAGTCACTATTTTTCCAGGCCTGAAGTGAGTTAT
AGGGCGAGACGGGTGTTGTATATTTATGTAAGGCAACAGCAGGGAGTTTAAGCGGCTGGATA
TTGCTGAAAGAGCATCATTCACATTCAGGCGGAGACAAAAGGTGGAAATGAAGCAACATCCT
GGCCAAAGAAGGCCTCAAGACAGAATAATAACAGTTCAGAGAGGGGGGCTGTGTGCACGGCC
GAGGGTCGGCCTCAAAACCAGGAAATGATCGAGATGCCTTGTCAGATCTTC

CoupTFII-965mutSBE-PEAK

GCTGAGACAAATGGAAAGCTGAAGATAAGGATCCTCTGAGGTGCGAACATACAGCTGTTGGG
AATTGCCAGAGAATCGGACCAATAAAGGAAGTCACTATTTTTCCAGGCCTGAAGTGAGTTAT
AGGGCGAGACGGGTGTTGTATATTTATGTAAGGCAACAGCAGGGAGTTTAAGCGGCTGGATA
TTGCTGAAAGAGCATCATTCACATTCAGGCGGAtcCAAAAGGTGGAAATGAAGCAACATCCT

GGCCAAAGAAGGCCTCAAGACAGAATAATAACAGTTCAGAGAGGGGGGCTGTGTGCACGGCC
GAGGGTCGGCCTCAAAACCAGGAAATGATCGAGATGCCTTGTCAGATCTTC

CoupTFII-965mutCONT1 (for zebrafish)
GCTGAGACAAATGGAAAGCTGAAGATAAGGATCCTCTGAGGTGCGAACATACAGCTGTTGGG
AATTGCCAGAGAATCGGACCAATAAAGGAAGTCACTATTTTTCCAGGCCTGAAGTGAGTTAT
AGGGCGAGACGGGTGTTGTATATTTATGTAAGGCAACgggtaacggtaTAAGCGGCTGGATA
TTGCTGAAAGAGCATCATTCACATTCAGGCGGAGACAAAAGGTGGAAATGAAGCAACATCCT
GGCCAAAGAAGGCCTCAAGACAGAATAATAACAGTTCAGAGAGGGGGGCTGTGTGCACGGCC
GAGGGTCGGCCTCAAAACCAGGAAATGATCGAGATGCCTTGTCAGATCTTC

Nrp2+26 enhancer (mouse)
Primer F ccagcatgagacaacttagca
Primer R gagttgtccccacagctatta
CCAGCATGAGACAACTTAGCAAGTCTTAGCTCTATCAGGTTTCACTTTCACCATCAG
AGTTTGAGTTGCTGTTACTGCTGCCAGTGAGGAGAGAGTGGTCTATGGGCCCTTGTT
ACCGACTTAGTACTCCGGCGTCATTACGGAACGCTTGCAAGGGGCTCTTGAAGTTGA
AGCTAAAGAATGAGGATTCTATAGTCATAAAAAGAAGCTGACGGTCATGCTGAAAAG
AGCCAGTCCCAAGCCTTCAACTTCACCAGTGGCAAAAAAAAAAAAAAAAAAAAAAAA
AAAAAAAACACCCCTTACATAAGCAAGTGTTTTCTATCCAGATAGCCTCCGGAGTAC
CCGCTGGTACAGATCTGAGGCTCTGCGGCTTCAGGAATGTACCTTGCTGGGAAGCCA
GGGCAGTGTGGGGAGTAGTGTGCAACTTGAGGGCAGGGCAGGGCAGGGGCACCAGTG
GCTTTGTTGTGTGCCCTTCATTTTCTGAAAGTGACCCACAGAAGACTTATCAGACAC
TGTGTCAGGAGCAGCCTCCTTCCTCACGTAGAGAGGGTTCTTGGCAGCCTGCGACCC
AGGGAACAATGAGGTTCTGAGGCCCCTACATCATCCCGGGGCATCTGTGGAAAAACA
AGCAGCGATTATAAATAGATGGTATTTGCAGTTTCCACTTTCTTGCCTTCAAGGTTT
TGTAGAATAACTCCTCCAGTTTGTTTTGGGTTATTCTCTGTGCATACTTCCTTTGCT
GGGTGGTCAGATTTCCTCCCTCCTAACGAGCGTGGTGACCCTGGCTGGGAGGGGACT
GCAGACGAAAAGCTGCAAACATCAGAGATTTGTGGCTTGAAGAAATGAAGGAAGAAA
GCGCAACTGCACACAGCTGCTGTGGAAAAGGCACTGGGCCCTAGTGCAGACACACAA
ACAACCCTTGAGTGGTAGACCCGCTGAGGCTTTAGAGATAGATGAATAATAATGACA
A

EMCN-22 enhancer (human)
ACTTTCATTCATTTGTTTATCCAGCATATCTTAACAGTCTATTATATGTTCATTATTGTTCT
AGGCACTAGGGACAAAATGGTGAGCACCAGCAAAGTTCTTCTCATGAGGCTGGCAATATTCA
TGAGAAGTTTGGTTGGATTGGGGCTGGAAAAAGATATTAATCAAGTATGCACAGTAATTGTC
CATTTTAAACTCTATTTGATATGCTATGACAAAATATTATTTGAAAATTTGATTTAGTAGAA
TTTGTATAAGTGAAAGATTTTATCCAGTTAGGAAGATATGTCAGGGCTTTCCTGGAAAAGTG
ACAATTACATTTCAATTTGAAGGATATTCAAGAGTTAACAAGTCAAAGTAACTGGGGCTGAA
AGGAAACGGGGATGACCACTCCAGACAGGAAGAAAACCCACAAAGGTCTTGTGGCAAGAAGT
AACACGGAAGGGACAAGGTACAGAAGGGAGGCCAATGTGTCTGGAAAGTTAGTGCAATGACA
GACACACAGGGTGAGATAAGGCTGCAAAGGAAGTTGAGGAGGATGAGGTAAGGGTTTGTGGG
CTATGTTAAGAATATAAGCCTCTATGATAAGAGCATAGGAACAGATTTGCGTTTTGACAAGA
TCACCCAGTCTCCTGTATTGAGAATGGATTTGACCGAGGTAAGAGCAAATGTGGGAGTCTAG
TAAAAAAGTTACTGAAAAATTCCAGGCACAAAATGATAGAAAACTTGAACTAGAGTGAAGGT
AGTACATATGAAGAGAAATGAATAAATTAGGGGGAAACTTGGAAGGTAATATTGAGAGCACT
TGGTGAAGGATTCAATTAAGGATTGAGGAAGAGTGAAATGTTGCTAAGTTTCTGCCTTACAA
AATTCAGAGAATAGCAGTGTCATTTACAGTGGTAAAGAACAAAAAAACTGACAAGGTTTCAG
GAGGAGCTCATAAGTTTGGCTTTGCAGATGTTGAGGTTTAGGCTCTCTTGAGCTATTCAAGA
AGAGATGCAAATAGGCACCTGCATTCTAT

Dll4in3 enhancer/promoter sequence

CTGCTGATATCGCTATCTCTAATGTCCCCCACCCCCCCTTTTGCTTCCCAGGGAACCTTCTC
ACTCAACATCCAAGCTTGGCACACACCGGGAGACGACCTGCGGCCAGGTGAGTATCTAACTT
CTCGGCCACAGGGGGGCGACATCACACAGCGCCGAAAGAGTTAACCAGTTATAGGCGGGGGT
GGGGGTTGGGGACGCAGGCTTGGGGGGTGGGGGCCAGGACGCTTAGCTTGGCCGGAGCTGCG
CCCCGCGCTGGACGCTCGGATTCCGCTCGCTGCCTGGACTCAGAGCACAATTGCGTTTCCTG
CGGGTTATTTTTGGCGTGGGAACGCGGGGAGCACGGCGGTGAGAAAGGCCGAGGCTGCCAGC
GCCGCTGACGGGCCTCTTCCTGTATTTTACACCTTTTGCGAATTCCGCTCCTTTGGAAAGGG
AATAATGGCTTTGGGATGTTGTTCTGACACAGAGGAAAAGGATATTTCACCAGCACAACAAT
TCTCACTTTGAAAAGGAAAAAGAAAAACCATTACCTACGTCTAGAACAGAACCCCTTGCTCC
CAGTTCTCGAACCAGAAAACTTCCCCCTTTAAATTTTTTCTTTTTTTCCATTTTGACCTCTT
TTCCTCTTTCCCCTCCGTATCTGCCTCCACAACCCTAGGATATCTTAACATCCGTCCATTGT
ACCCTTTTTTGAATGCTATCAAGCCCCCTGCACATGCACACACCCAGGGAGACTAAGTAGCA
AGATTCTGGGACCCTCTGGCCTGTGCTTACTTGCAGGTAGAGTTAATCTAGATAATTAGAGT
GTGAACTGACCACCATAGTCACAACTAAAGAGAGAGTTGGCAGCAGTCAACTCTCTCTGAAT
CAGGTTGGCTTTCTGAATCAGGTTCTCTGACCAAAGCCTCTTTCTGCAGAGACTTCGCCAGG
AAACTCTGCGGCCGCTCTAGAACTAGTCTGGAAAGGAAAGGGAGATCCAAATCCCCTGGTCC
TGCTTTTTGCTTTCCTAGTTTAAGCTTTCCCCACCTGCTAGAGGACTGTAGGTATCTAATGC
CTGGATCAGGTGCACCGCCTACGGGGACCCCTTAGAGTTTCCACCCCCTGGACCATTCGGGA
ACCACCTCACCTCCCGCCGCATCACTGGGCTACCCTCCTATCCTCTGGTGGCGAGGGTCTCA
GCCTTTAAGCAGACGATCTCTAAGGACTGCTCGCCGGGCACGCGCAGAGCTGGAAGCCCAGA
AGTTGGAAGAGGGGCGGGGACCTGCGCCCTACTGGCTGGCTGACAGGGGGAGCGGCGGGGGC
GGAGGCCCCCTCCGGTGGGTGCTGGGACTGTAGCCACTAGAGGCCTGGAGGGGAGGGGAGAG
TGACCGTGAGTCTGTCTGACTGACAGGCTGCGAAGAGCAGCCAATATATATAAGAAAGGCTC
TGGAGCAAGCAGGTTTCAGTAGCGGCGCTGCTCGCAGGCTAGGAACCCGAGGCCAAGAGCTG
CAGCCAAAGTCACTTGGGTGCAGTGTACTCCCTCACTAGCCCGCTCGAGACCCTAGGATTTG
CTCCAGGACACGTACTTAGAGCAGCCACCGCCCAGTCGCCCTCACCTGGATTACCTACCGAG
GCATCGAGCAGCGGAGTTTTTGAGAAGGCGACAAGGGAGCAGCGTCCCGAGGGGAATCAGCT
TTTCAGGAACTCGGCTGGCAGACGGGACTTGCGGGAGAGCGACATCCCTAACAAGCAGATTC
GGAGTCCCGGAGTGGAGAGGACACCCCAAGGG

Probes used for in situ hybridization

<u>Zebrafish *ephb4a in situ* probe</u>
TCTCAGCTCTGGACAAGCTGATCCGCAACCCGGCCTCACTCAAAATCACAGCGCAGGAGGGG
GCGGGCCCCTCTCACCCTCTGCTGGACCAGCGGTCTCCACTCACGCCCTCATCCTGCGGGAC
AGTGGGTGACTGGCTGCGGGCCATCAAGATGGAGCGCTACGAGGAGACATTTCTGCAGGCGG
GATACACGTCCATGCAGCTCGTCACCCACATCAACACGGAGGATCTGCTGCGTTTGGGAATA
ACTTTAGCAGGTCACCAGAAGAAGATTCTCTCCAGCATTGAGGCTCTCGGGATTCAAAACAA
AGCACCAGGGAATGTGCTGTACTGA

<u>Zebrafish *efnb2a in situ* probe</u>
AAAACCAAGTCGATGAAAATCATCATGAAGGTTGGACAAAACCCCTCTGATCCCATTTCCCC
CAAAGACTACCCTACCAGTTACCCTCCCAAACACCCTGACTTAGGGGGCAAGGACAGCAAAT
CGAATGAAGTACTTAAGCCAGATGCATCTCCTCATGGGGAAGATAAGGGAGATGGAAATAAA
TCCTCATCAGTCATTGGCTCAGAGGTGGCCCTGTTTGCCTGCATCGCCTCAGCAAGCGTCAT
CGTCATCATCATAATCATCATGCTAGTTTTCCTTCTCCTGAAGTATCGACGA

<u>Zebrafish *alk2 in situ* probe</u>
GGACGCACACTGACAGGCCTTGAAGAAACCCTCTGTCATGGATTTTTTTAACAGCATCATGT
TGTCTATTTAACCATTGGCAGACTTTAAAGATTTGTACTTTTCAGAAAAATGAAAAGGACGG
ATACACACATGCTGAAATGCAATCAGTCTCATAATGTGTGTGTGTGCACATGATTCTTGTTA
CAACCAGAAAAACTTCATAGCTGTAACATCACCTGCTTTTAGACATCTGGTGGGTTTTGAGC
TTTTTAAAGGTCCTGATAGTTCTTGTAGATAGGTTAGGCAAAGTTAGTCACAGTTTTGCTAT
ATGGACTTATCATAAAACCGGTTGGTTGCTTAATGCCACTATGTACAGTAGGAATCAACAGG
CTTCTTTCTTTAATTAGGCCTGTTTAGTGACCAGACCTGGGTTTTGAACTAGTGGTAA

Zebrafish *alk3a in situ* probe

CACAGAGGAGGAGACTCAATCTGACACTCAAGTAAATCAAGCTCTCCTCCTCTGTTGGATCA
GACCTGGTGCCTTTGCTGCTTTCTGTGAAAGCTGTATGGTTGTGGACTTGGATCAGTGGATT
ACTGCGCCAGAGTGGGATGTTCTGGTGGATTTGGGAAAGACTGATATAAACCCACCTGCCTT
AAAATACACTCAGCTGTTTCTGTCGATTCTGGGATAAATCTCCTGCCATATGGATGAATTGC
TGTTGACTTCTCATGGAATCTATGTAAAGCAAACTGTGATTTAATGTATGATGTAAATATAG
TAATAGGTGAACTGAAAACAGAGCAAAACAATATTCTATCAATATAATCTCGTCCAGTAACA
GCCGTTGTATAATATCTAGAGGAAGATTATTTGTGCCTCTAGTTGTCTGATGTGTGGCTT

Zebrafish *alk3b in situ* probe

TGTCATGTAGCACTCTCTGCTGGACAGTATGTGCCATAACGCATCTATGGGGAACCAAGGAC
GAAAAGGACAACTTATCTACAGGCATCGGTACTAACATCAGCCCCATCGCCACTATGTTTTC
TATTTTCAGGAACTCTTATGTTTTAGGAGAGAACTGAAAAGCATATGGTGCTTTCTGTGAAA
GCCATGAAATACGATACTTTGGTATGGAGGACTAGCATGATTTGTCATGGGAGCGCAGTAAT
GGAGCCATTCGGGAGCCAAGGTTTAAGCAAATGACTTGAACAGTGGACATATTTGGGGTGAT
TTTTGGACTGGACCCCATCCATATTCTTGGGATGTGCTGTGTTGTGGTTAACAACACAAAAT
GGACACTGCATTTGAGCGATGTGTTGTAGTGGCCACCGAATTGCAGCTCA

Mouse *Bmp4 in situ* probe

CCTGCAGCGATCCAGTCTCTGGCCCTCGACCAGGTTCATTGCAGCTGTCTAGAGGTCCCCAG
AAGCAGCTGCTGGCGAGCCCGCTTCTGCAGGAACCAATGAGACACCATGATTCCTGGTAACC
GAATGCTGATGGTCGTTTTATTATGCCAAGTCCTGCTAGGAGGCGCGAGCCATGCTAGTTTG
ATACCTGAGACCGGGAAGAAAAAAGTCGCCGAGATTCAGGGCCACGCGGGAGGACGCCGCTC
AGGGCAGAGCCATGAGCTCCTGCGGGACTTCGAGGCGACACTTCTACAGATGTTTGGGCTGC
GCCGCCGTCCGCAGCCTAGCAAGAGCGCCGTCATTCCGGATTACATGAGGGATCTTTACCGG
CTCCAGTCTGGGGAGGAGGAGGAGGAAGAGCAGAGCCAGGGAACCGGGCTTGAGTACCCGGA
GCGTCCCGCCAGCCGAGCCAACACTGTGAGGAGTTTCCATCACGAAGAACATCTGGAGAACA
TCCCAGGGACCAGTGAGAGCTCTGCTTTTCGTTTCCTCTTCAACCTCAGCAGCATCCCAGAA
AATGAGGTGATCTCCTCGGCAGAGCTCCGGCTCTTTCGGGAGCAGGTGGACCAGGGCCCTGA
CTGGGAACAGGGCTTCCACCGTATAAACATTTATGAGGTTATGAAGCCCCCAGCAGAAATGG
TTCCTGGACACCTCATCACACGACTACTGGACACCAGACTAGTCCATCACAATGTGACACGG
TGGGAAACTTTCGATGTGAGCCCTGCAGTCCTTCGCTGGACCCGGGAAAAGCAACCCAATTA
TGGGCTGGCCATTGAGGTGACTCACCTCCACCAGACACGGACCCACCAGGGCCAGCATGTCA
GAATCAGCCGATCGTTACCTCAAGGGAGTGGAGATTGGG

Mouse *Bmp2 in situ* probe

GATCTTCCGGGAACAGATACAGGAAGCTTTGGGAAACAGTAGTTTCCAGCACCGAATTAATA
TTTATGAAATTATAAAGCCTGCAGCAGCCAACTTGAAATTTCCTGTGACCAGACTATTGGAC
ACCAGGTTAGTGAATCAGAACACAAGTCAGTGGGAGAGCTTCGACGTCACCCCAGCTGTGAT
GCGGTGGACCACACAGGGACACACCAACCATGGGTTTGTGGTGGAAGTGGCCCATTTAGAGG
AGAACCCAGGT

Chromatin immunoprecipitation (ChIP)

Ephb4-2 Enhancer TaqMan probe

CCCTTCCTTGCTGTTCTGCCTGGGTCCTGCGCCCGGGTTGGGGGGGGTGGGCCGGTCACCGA
GGGCAGGAAACAGCCGGCTTCACTGGAGCCAGGCAGAC

Negative Control Probe

CCTCAGCCTCCCAAGTAGCTGGGATTACAGGTGTGTGCTACCATGCCTGGCTAATTTTTGTA
TTTTTAGTAGAGACAGGGTTTCACCATGTTGGCCAGGCTGGTCTCGAACTCCTGAACTCAGG
TGATCTA

Supplementary Figures

Supplementary Table 1

Primer Name	Sequence (5'-3')
Dll4in3 Forward	ATATCCGCGGCTGCTGATATCGCTATCTC[1]
Dll4in3 Reverse	ATATGCGGCCGCAGAGTTTCCTGGCGAAGT[1]
DLL4 promoter Forward	AGTAGACTAGTCTGGAAAGGAAAGGGAGATC
DLL4 promoter Reverse	ATAATGGATCCCCCTTGGGGTGTCCTCTC
smad1 MO	AGGAAAAGAGTGAGGTGACATTCAT [2]
smad5 MO	ACATGGAGGTCATAGTGCTGGGCTG [2]
alk1 MO	ATCGGTTTCACTCACCAACACACTC [3]
alk2 MO	GATTCATGTTTGTGTTCAATTTCCG [3]
alk3a MO	GACGCATTGTCAAATTGTCTTGTCG [4]
alk3b MO	GTCGAGTTGTTGAACTGTATGGCTG [4]
bmpr2a MO	AGAGAAACGTATTTGCATACCTTGC [5]
bmpr2b MO	AGTTGATTCTGACCTTGTTTGACCA [5]
dab2 MO	TTCTGCTTCAGGTGACTGTGACATG [6]
tnnt2 MO	CATGTTTGCTCTGATCTGACACGCA[7]
LacZ genotyping Forward	GTCGTTTTACAACGTCGTGAC
LacZ genotyping Reverse	GATGGGCGCATCGTAACCGTG
Smad4 genotyping Forward1	CTTTTATTTTCAGATTCAGGGGTTC[8]
Smad4 genotyping Forward2	AAAATGGGAAAACCAACGAG[8]
Smad4 genotyping Reverse1	TACAAGTGCTATGTCTTCAGCG[8]
Alk3 genotyping Forward1	GCAGCTGCTGCTGCAGCCTCC[9]
Alk3 genotyping reverse1	TGGCTACAATTTGTCTCATGC[9]
Alk3 genotyping Forward2	GGTTTGGATCTTAACCTTAGG[9]
Alk3 genotyping Reverse2	TGGCTACAATTTGTCTCATGC[9]

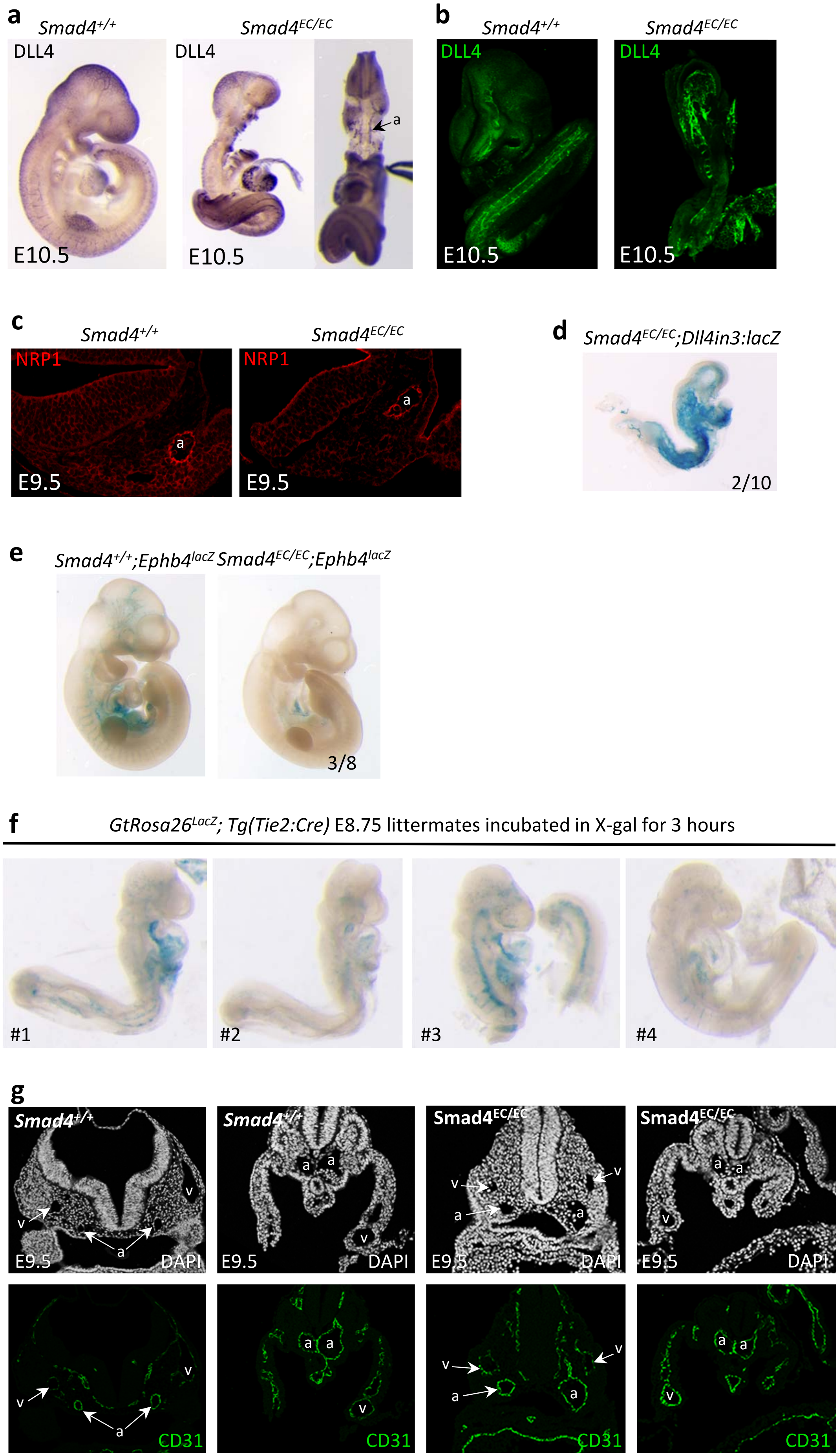
a
Smad4+/+
Smad4EC/EC
DLL4
DLL4
a
E10.5
E10.5
b
Smad4+/+
Smad4EC/EC
DLL4
DLL4
E10.5
E10.5
c
Smad4+/+
Smad4EC/EC
NRP1
NRP1
a
a
E9.5
E9.5
d
Smad4EC/EC;Dll4in3:lacZ
2/10
e
Smad4+/+;Ephb4lacZ
Smad4EC/EC;Ephb4lacZ
3/8
f
GtRosa26LacZ; Tg(Tie2:Cre) E8.75 littermates incubated in X-gal for 3 hours
#1
#2
#3
#4
g
Smad4+/+
Smad4+/+
Smad4EC/EC
Smad4EC/EC
a
v
E9.5
DAPI
CD31

Supplementary Figure 1, relating to Figure 1 in main text**.**

Endothelial-specific loss of *Smad4* does not affect early arterial identity and differentiation.

a-b. Whole-mount DLL4 immunostaining in the arteries (**a**) of both E10.5 *Smad4*$^{+/+}$ and *Smad4*$^{EC/EC}$ embryos (**a**, detected by immunohistochemistry; **b**, detected by immunofluorescence).

c. Expression of the arterial endothelial cell marker NRP1 in transverse sections from E9.5 *Smad4*$^{+/+}$ and *Smad4*$^{EC/EC}$ embryos.

d. *Smad4*$^{EC/EC}$*;Dll4in3:lacZ* E10.5 embryo, representative of 2/10 embryos examined. Image shown in Fig. 1 is representative of the other 8 embryos.

e. *Smad4*$^{EC/EC}$*;Ephb4*^{lacZ} E10.5 embryo, representative of 3/8 embryos examined, comparative to *Smad4*$^{+/+}$*;Ephb4*^{lacZ} littermates. A small amount of LacZ staining can be seen around the heart region.

f. Expression of the *R26R:LacZ* reporter gene in Tie2:Cre+ E8.75 embryos demonstrates that Cre activity can vary between embryos within a single litter.

g. DAPI (white) and CD31 (green) immunostaining on representative transverse sections from E9.5 *Smad4*$^{+/+}$ and *Smad4*$^{EC/EC}$ embryos. Some venous structures can be seen in both WT and KO embryos.

Supplementary Figure 2

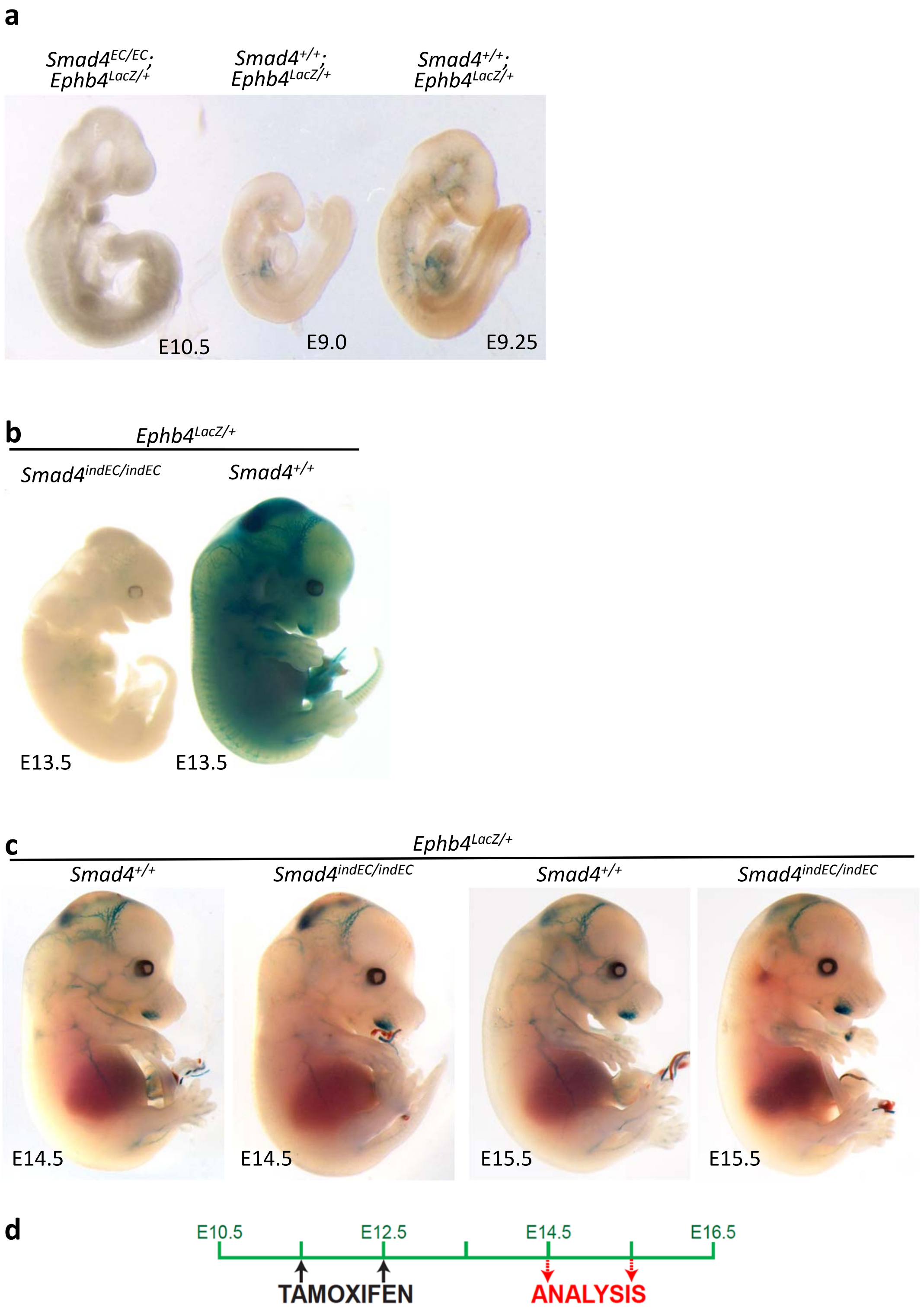

Supplementary Figure 2, relating to Figure 2 in the main text.

Induced endothelial-specific deletion of *Smad4* after the vasculature has formed also leads to reduction of *Ephb4*^{LacZ} expression.

a. X-gal staining in a representative E10.5 *Smad4*$^{EC/EC}$;*Ephb4*$^{LacZ/+}$ embryo compared to that seen in similarly sized but younger *Smad4*$^{+/+}$;*Ephb4*$^{LacZ/+}$ embryos.
b. E13.5 *Smad4*$^{indEC/indEC}$; *Ephb4*$^{LacZ/+}$ embryo shown in Figure 2 with littermate wild-type control.
c-d. Representative wild-type and induced endothelial-specific *Smad4*$^{fl/fl}$ whole-mount *Ephb4*$^{LacZ/+}$ embryos. Tamoxifen was administered at E11.5 and E12.5 as denoted by the schematic (**d**).

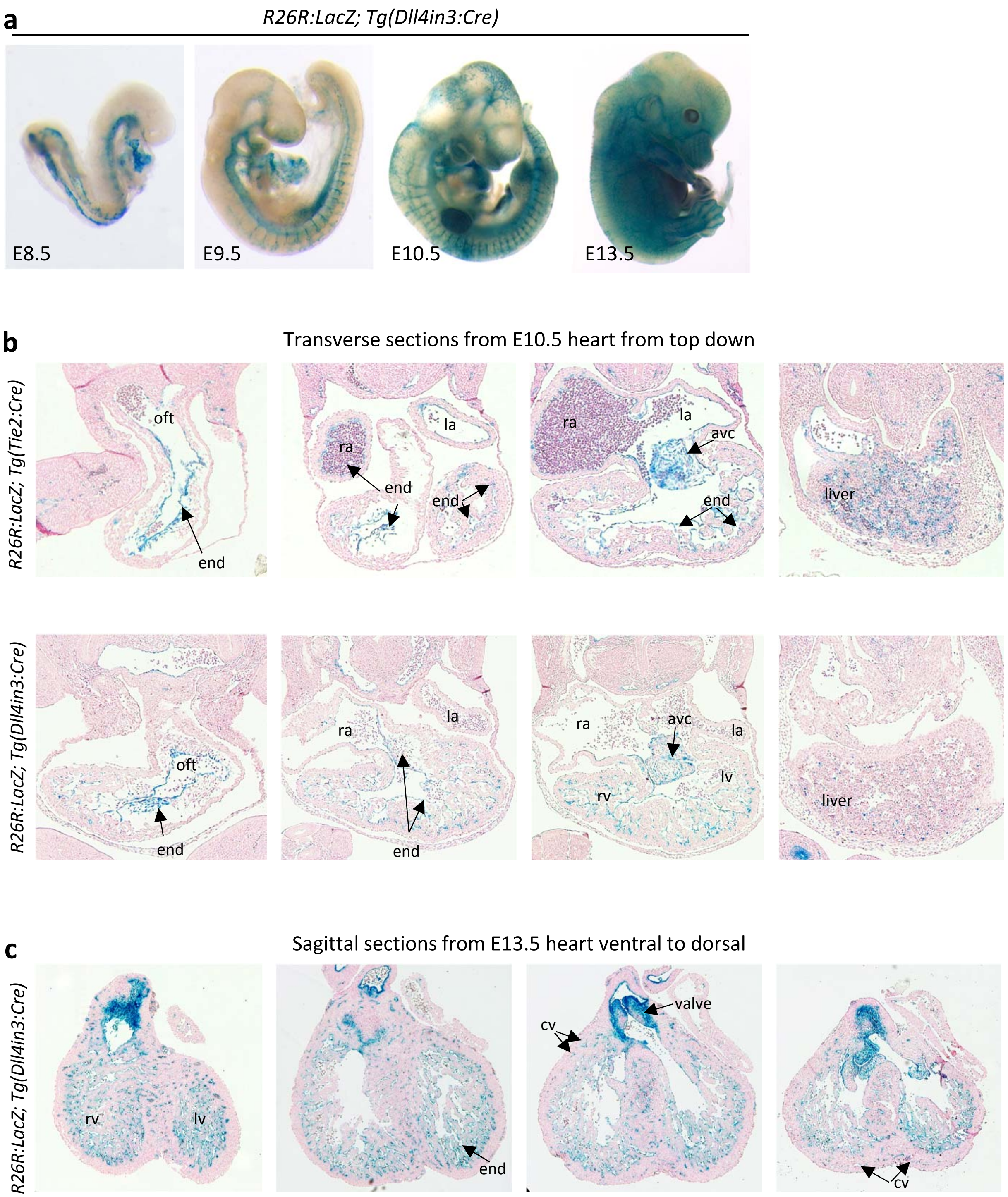
a
R26R:LacZ; Tg(Dll4in3:Cre)
E8.5
E9.5
E10.5
E13.5
b
Transverse sections from E10.5 heart from top down
R26R:LacZ; Tg(Tie2:Cre)
oft
end
ra
la
end
end
ra
la
avc
end
liver
R26R:LacZ; Tg(Dll4in3:Cre)
oft
end
ra
la
end
ra
avc
la
lv
rv
liver
c
Sagittal sections from E13.5 heart ventral to dorsal
R26R:LacZ; Tg(Dll4in3:Cre)
rv
lv
end
cv
valve
cv

Supplementary Figure 3, relating to Figure 3 in main text.

Dll4in3:Cre expression pattern in the developing embryo.
a. Representative *R26R:LacZ; Tg(Dll4in3:Cre)* embryos from E8.5-E13.5 demonstrating Cre activity driven by the arterial Dll4in3 enhancer.
b. Transverse sections through the heart (base to apex) of *R26R:LacZ; Tg(Tie2:Cre)* and *R26R:LacZ; Tg(Dll4in3:Cre)* E10.5 embryos. Outflow tract (oft), right and left atria (ra and la), right and left ventricles (rv and lv), atrioventricular cushion (avc).
c. Sagittal sections through the heart of a representative E13.5 *R26R:LacZ; Tg(Dll4in3:Cre)* embryo. Robust expression was detected in the endocardium (end), valves and coronary vessels (cv).

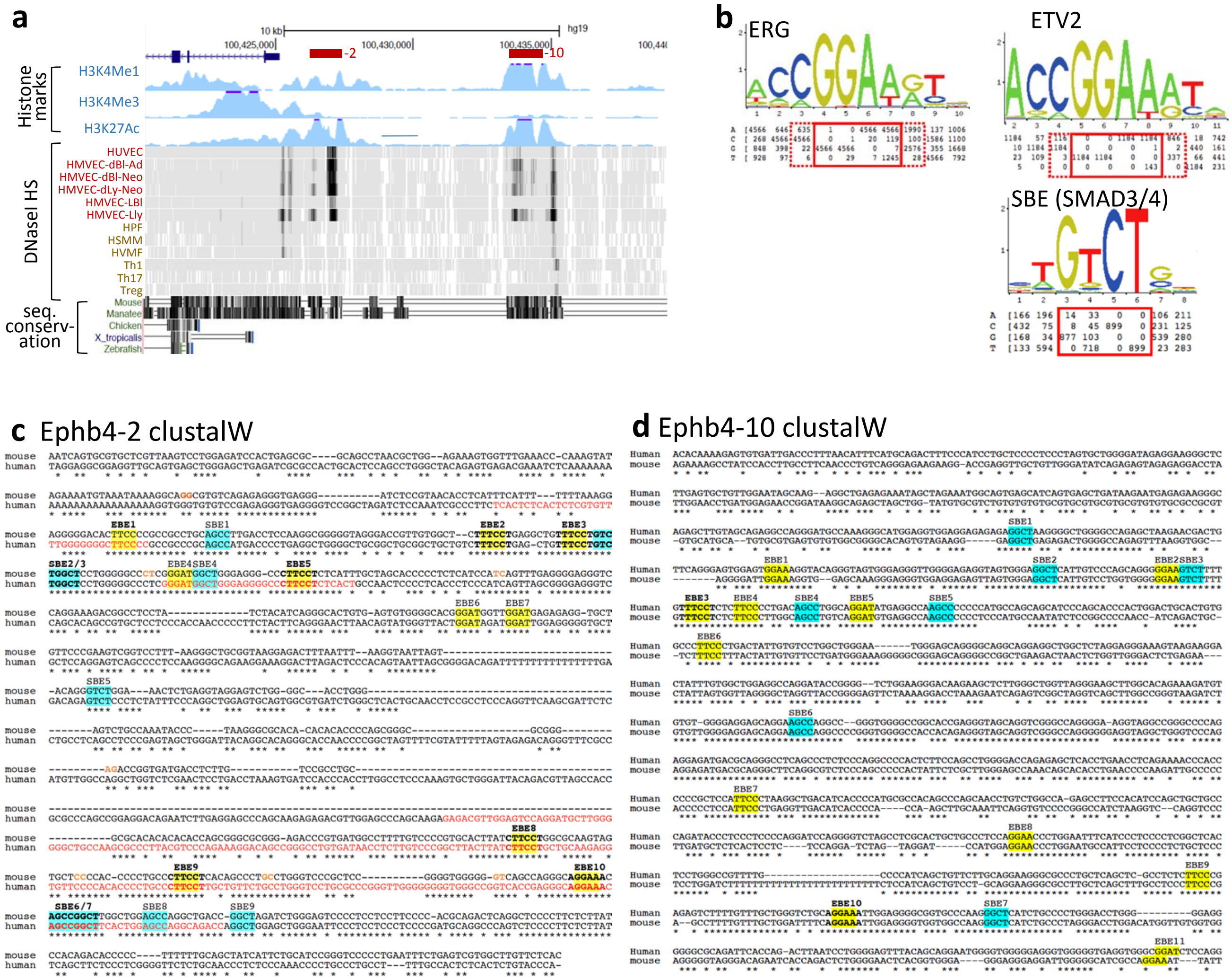

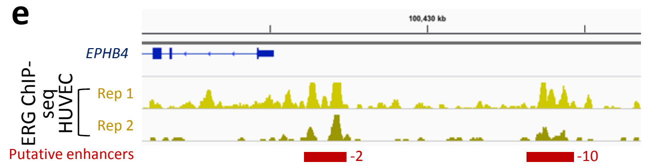

f

Enhancer construct	Number of fish screened	Fish with any detectable GFP % of total (# of fish)	**Fish with detectable vascular GFP % of total (# of fish)**
Ephb4-2:GFP	259	78 (202)	**59 (152)**
Ephb4-10:GFP	90	44 (40)	**0 (0)**

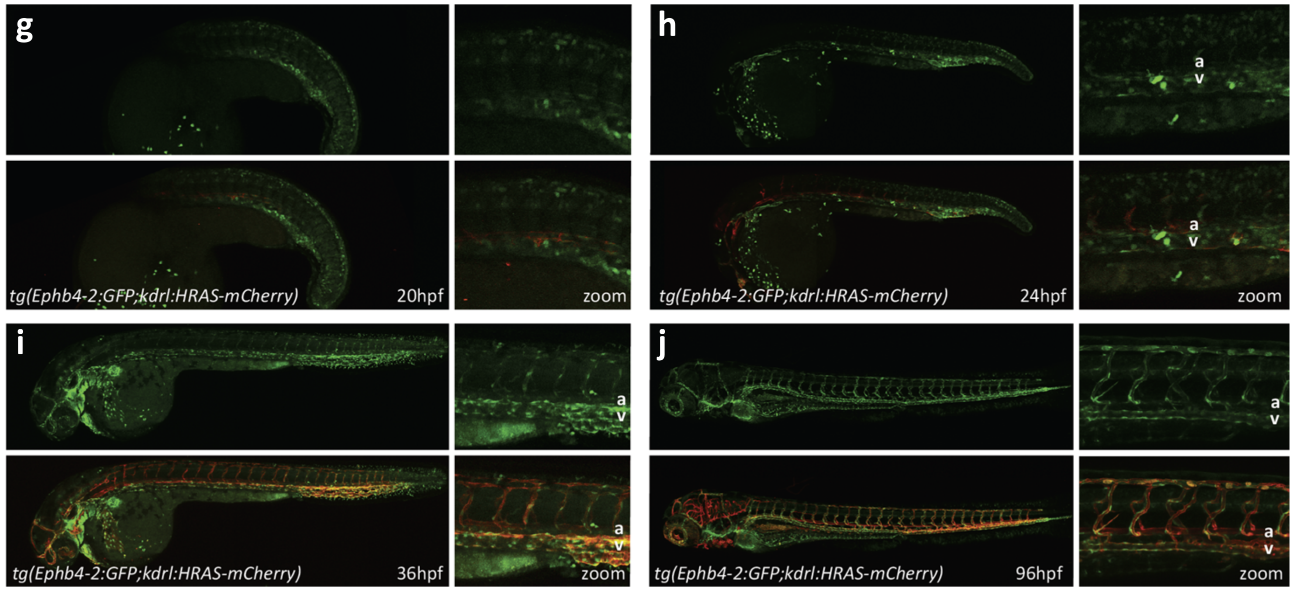

Supplementary Figure 4, relating to Figure 4 in main text**.**

Identification and analysis of the Ephb4-2 and Ephb4-10 enhancers.
a. The 5' upstream region of the human *EPHB4* gene as seen on UCSC Browser (http://genome.ucsc.edu). Histone marks (light blue) and DNase I hypersensitivity (HS, peaks shown as a heat map, different cell type names written on left with endothelial lines denoted in red) are concentrated around the two putative enhancers, marked as -2 and -10 and by red horizontal lines. Conservation between the human sequence and mouse, manatee, chicken, xenopus and zebrafish is indicated by black vertical lines, demonstrating that neither enhancer is conserved beyond manatee.
b. Probability weight matrices from JASPAR showing the consensus binding motifs for ERG, ETV2 and SMAD (referred to in text as the SBE). Red boxes denote the core base pairs used for analysis of Ephb4-2 and Ephb4-10 sequences. Dotted line denotes additional base pairs considered to decide which EBE most adhered to consensus.
c-d. ClustalW alignment of the Ephb4-2 (**c**) and Ephb4-10 (**d**) mouse and human enhancer sequence annotated with conserved ETS binding elements (EBE, yellow) and SMAD binding elements (SBE, blue). Numbers refer to mutations analysed in Figure 5 and Supplementary Fig. 5. Bold EBE refer to those that adhere to a more stringent consensus binding motif denoted by red dashed lines in b. Base-pairs mutated in mutCONT transgene are denoted in orange text.
e. IGV browser view of 5' region of *EPHB4* gene and upstream sequence incorporating ERG ChIP-seq data from Fish *et al*[10]. Both Ephb4 putative enhancers (red horizontal bars) have significant ERG binding peaks in HUVECs (yellow peaks).
f. Table summarizing Ephb4-2:GFP and Ephb4-10:GFP transgene activity as detected by GFP expression in 48hpf transient transgenic zebrafish.
g-j. Relating to Fig. **4c**, expression in the transgenic zebrafish line *tg(Ephb4-2:GFP; kdrl:HRAS-mCherry)* as shown by representative embryos at 20hpf (**g**), 24hpf (**h**), 36hpf (**i**) and 96hpf (**j**). a=dorsal aorta, v=dorsal/ventral veins.

Supplementary Figure 5

a

Tg(Ephb4-2:LacZ)

Smad4$^{EC/EC}$ *Smad4$^{+/+}$*

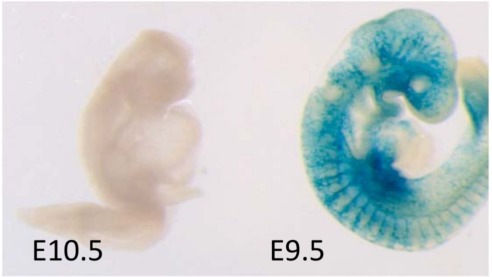

b

Tg(Ephb4-2:LacZ)

Smad4$^{+/+}$ *Smad4$^{EC/EC}$*

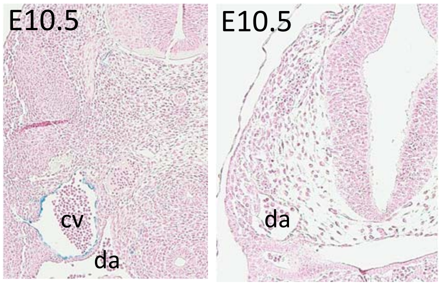

c

Ephb4-2 enhancer version	Number of zebrafish embryos screened	Zebrafish embryos with any detectable GFP expression % of total (# of fish)	Zebrafish embryos with detectable vascular GFP % (# of injected fish)
WT	259	78 (202)	59 (152)
mutSBE(all)	62	65 (40)	8 (5)
mutSBE(2/3,6/7)	70	74 (52)	7 (5)
mutSBE(2/3) done	60	77 (46)	15 (9)
mutSBE(6/7) done	70	74 (52)	3 (2)
mutCONT	51	80 (41)	70 (36)

d

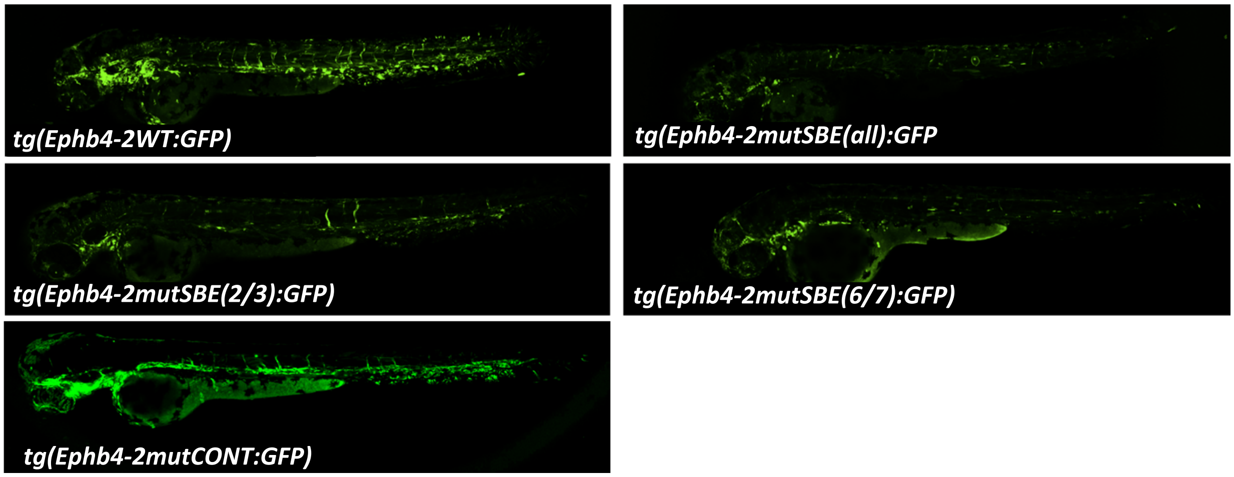

e

Ephb4-2 enhancer version	Number of transgenic mouse embryos screened	Mice embryos with any detectable *LacZ* expression	Embryos with *LacZ* in vessels % (#)
WT	**5**	**5**	100 (5)
mutSBE (2/3,6/7)	**7**	**5**	0 (0)

Supplementary Figure 5, relating to Figure 5 in main text**.**

Ephb4-2 enhancer activity requires SMAD4.
a. X-gal expression in comparably sized *Smad4*$^{EC/EC}$*;Ephb4-2:LacZ* and *Smad4*$^{+/+}$*;Ephb4-2:LacZ* transgenic embryos.
b. Representative transverse sections taken through the torso at the base of the heart from E10.5 *Smad4*$^{+/+}$ and *Smad4*$^{EC/EC}$ embryos also transgenic for Ephb4-2:LacZ. Cv indicates cardinal vein, da indicates dorsal aorta (section shown for *Smad4*$^{EC/EC}$ embryo can be directly compared to Fig. 1b to confirm da identification).
c. Table summarizing the reduction of GFP expression pattern in transient transgenic zebrafish after mutations of SMAD binding elements (SBE), and other conserved regions (CONT), comparative to Ephb4-2:GFP wild-type. The numbers after SBE and EBE correlate to the motifs highlighted in Supplementary Fig. 4c, mutated nt in CONT are marked in orange text in Supplementary Fig. 4c.
d. Representative 48hpf transient transgenic zebrafish expressing wild type and mutated Ephb4-2 sequences detailed in **c**.
e. Table summarizing the reduction of X-gal expression pattern in transient transgenic mice expressing Ephb4-2mutSBE(2,3/6/7):*LacZ*, in which two composite SMAD binding motifs have been mutated, compared to Ephb4-2:*LacZ* wild-type.

Supplementary Figure 6

a Over-represented motifs found in SMAD1/5-bound regions by Morikawa et al., 2011

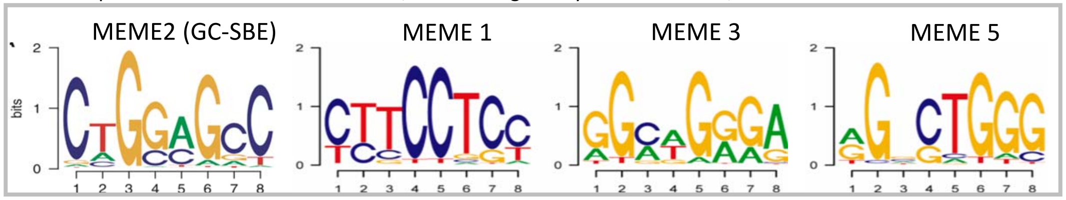

b

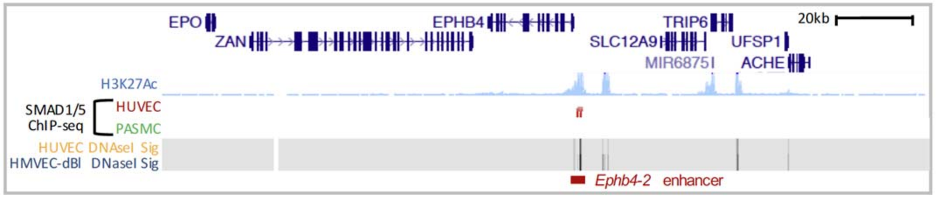

c

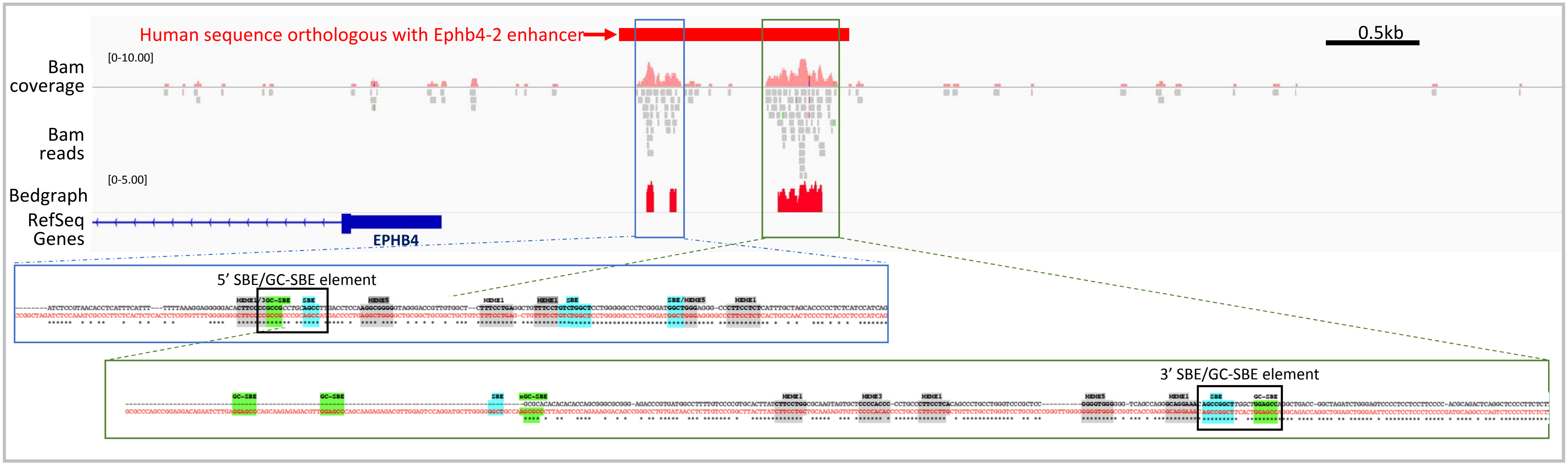

Supplementary Figure 6, relating to Figure 6.

SMAD1/5 binding is detected over the Ephb4-2 enhancer sequence.
a. Reproduction of sequence logos for over-represented motifs associated with endothelial SMAD1/5-binding taken from Morikawa et al. [11].
b. UCSC genome browser view of the *EPHB4* gene locus and surrounding 200kb region. HUVEC specific H3K27Ac peaks are marked in blue, statistically significant SMAD1/5 binding peaks from ChIP-seq data in HUVECs and PASMCs [30] marked in red and green, and DNase hypersensitivity sites in HUVEC and HMVEC cell lines are shown as black heatmap.
c. IGV view of the human sequence orthologous to the Ephb4-2 enhancer and surrounding region. Morikawa et al. SMAD1/5 ChIP-seq data in HUVEC (BMP9 stimulated) is displayed as Bam coverage (pink peaks), Bam reads (grey boxes) and Bedgraph (red peaks). The exact sequence corresponding to Bam coverage peaks is shown in full, mouse sequence on top, human sequence on bottom, * denoting conserved bp. Motifs are marked: SBE (blue), GC-SBE (green) and other SMAD1/5-bound MEMEs (grey). The two black boxes indicate the SBE/GC-SBE composite elements.

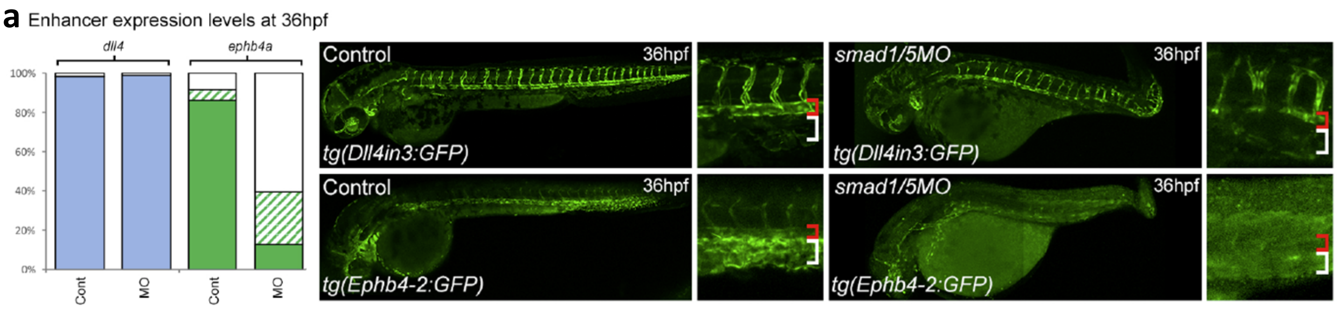

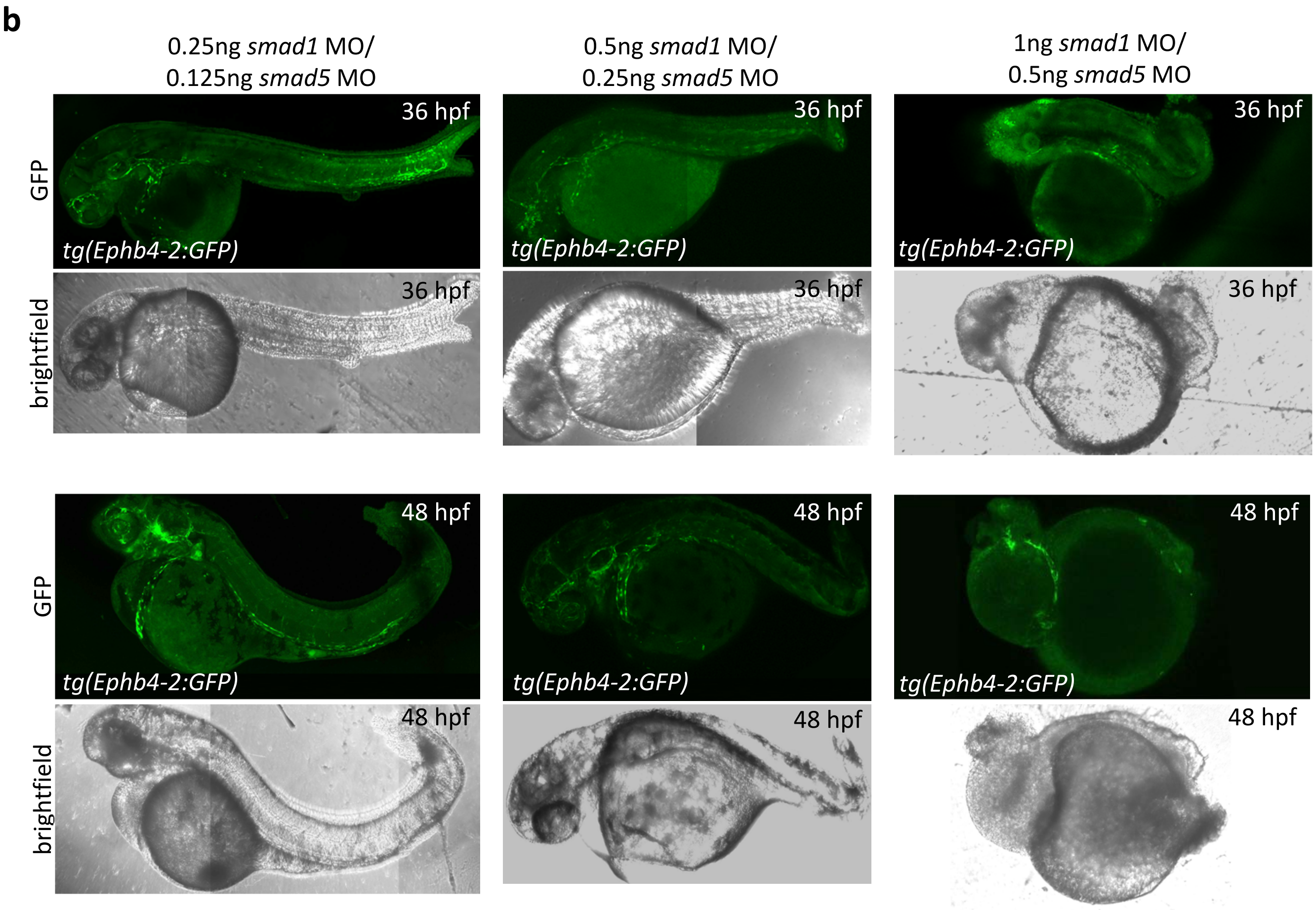

c

Ephb4-2:GFP expression at 36hpf

	% strong GFP	% low GFP	% no GFP
Low dose *smad1/5* MO	63	36	0
Med dose *smad1/5* MO	15	25	60
High dose *smad1/5* MO	0	0	100

Ephb4-2:GFP expression at 48hpf

	% strong GFP	% low GFP	% no GFP
Low dose *smad1/5* MO	19	62	19
Med dose *smad1/5* MO	8	8	84
High dose *smad1/5* MO	0	0	100

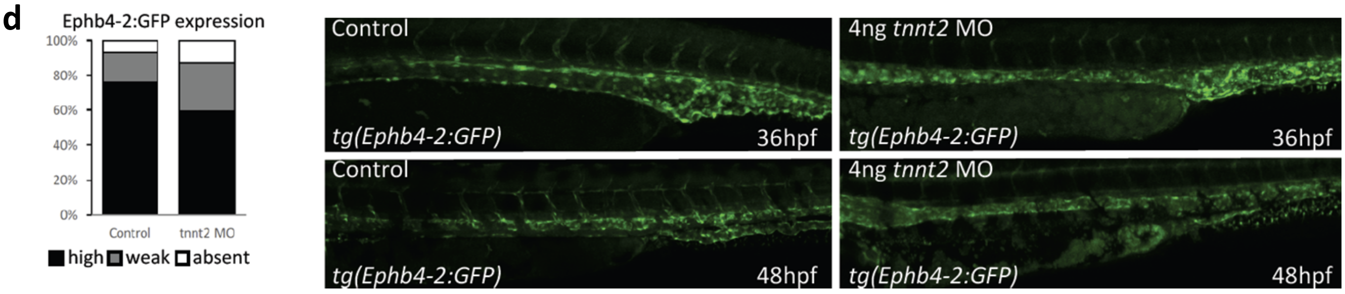

Supplementary Figure 7, relating to Figure 6 in main text.

MO-mediated SMAD1/5 knock-down in *tg(Ephb4-2:GFP)* and *tg(Dll4in3:GFP)* transgenic zebrafish

a. Morpholino (MO)-mediated depletion of *smad1/5* in zebrafish lines transgenic for the Dll4in3:GFP arterial and Ephb4-2:GFP venous enhancers at 36hpf. The graphs depict observed expression in all embryos (Dll4in3:GFP WT n=60 MO n=64; Ephb4-2:GFP WT n=67, MO n=86). High expression levels are represented by solid colour, weak levels by pattern and absent expression by solid white. Zebrafish embryos shown are representative of the predominant phenotype, red bracket=dorsal aorta, white bracket=posterior cardinal and ventral vein(s)**.**

b-c. Dose-response analysis of Ephb4-2:GFP expression and morphology using different concentrations of smad1 and smad5 morpholinos. Representative embryos are shown in **b,** and expression patterns summarized in **c**. 0.25ng smad1MO/0.125ng smad5MO embryos n=52 at 36hpf, 52 at 48hpf; 0.5ng smad1MO/0.25ng smad5MO embryos n=48 at 36hpf, n=86 at 48hpf; 0.1ng smad1MO/0.5ng smad5MO embryos n=32 at 36hpf, n=49 at 48hpf.

d. Expression of Ephb4-2:GFP in zebrafish after loss of blood flow downstream of morpholino-mediated depletion of *tnnt2*. Graph depicts observed expression pattern of GFP in *tg(Ephb4-2:GFP)* embryos for control (n=46) and *tnnt2*-MO injected embryos (n=47), black represents high expression, grey represents weak, white represents absent expression.

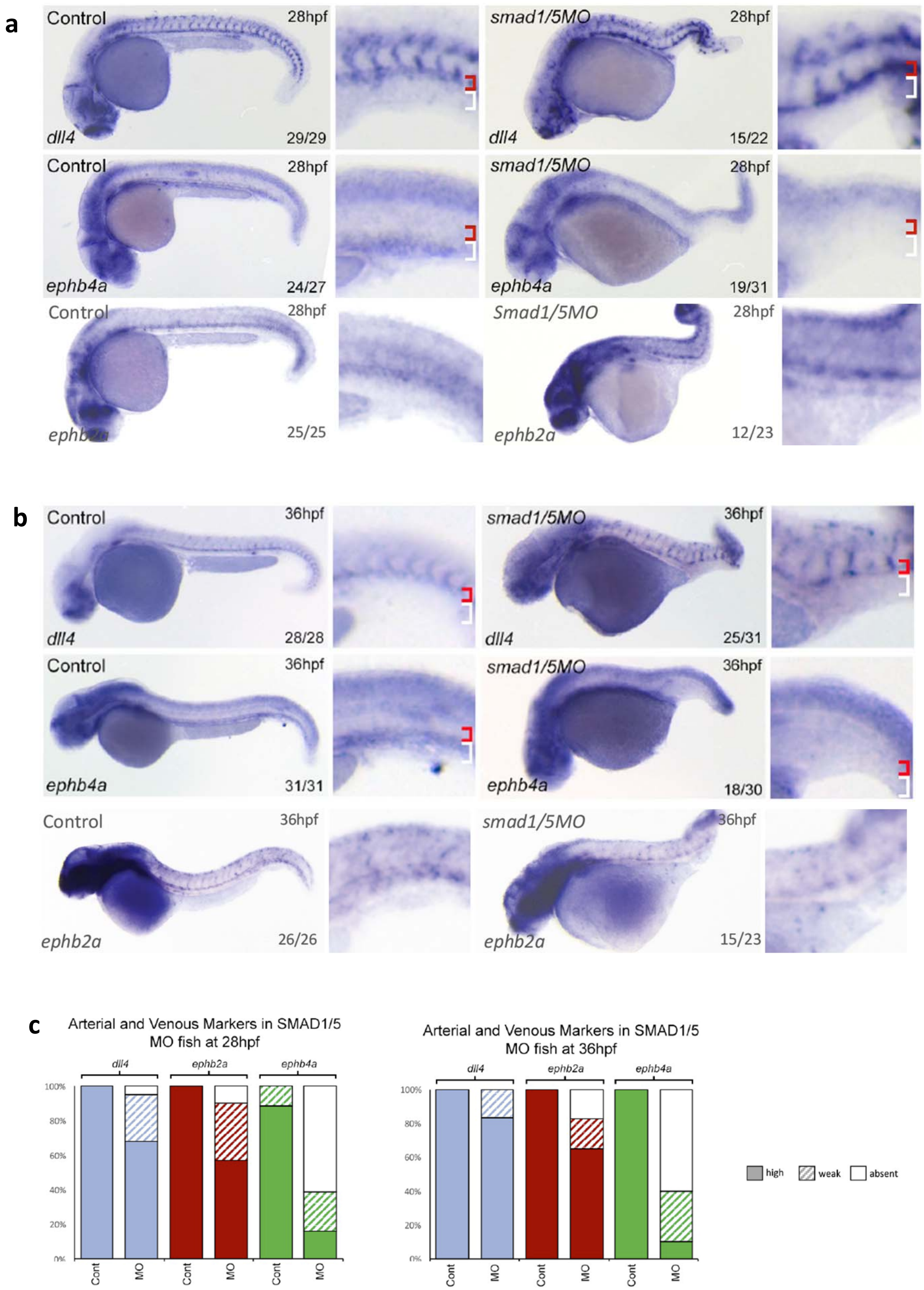
a
Control
28hpf
dll4
29/29
smad1/5MO
28hpf
dll4
15/22
Control
28hpf
ephb4a
24/27
smad1/5MO
28hpf
ephb4a
19/31
Control
28hpf
25/25
Smad1/5MO
28hpf
ephb2a
12/23
b
Control
36hpf
dll4
28/28
smad1/5MO
36hpf
dll4
25/31
Control
36hpf
ephb4a
31/31
smad1/5MO
36hpf
ephb4a
18/30
Control
36hpf
ephb2a
26/26
smad1/5MO
36hpf
ephb2a
15/23
c
Arterial and Venous Markers in SMAD1/5 MO fish at 28hpf
dll4
ephb2a
ephb4a
100%
80%
60%
40%
20%
Cont
MO
Arterial and Venous Markers in SMAD1/5 MO fish at 36hpf
high
weak
absent

Supplementary Figure 8, relating to Figure 6 in main text.

Expression of endogenous ephb4a, dll4 and ephb2a in zebrafish after MO-mediated smad1/5 knockdown.

a-c. Expression of *dll4*, *ephb2a and ephb4* as detected by whole-mount *in situ* hybridization after morpholino (MO)-mediated depletion of Smad1/5 at both 28hpf (**a**) and 36hpf (**b**). Graph (**c**) depicts observed expression of *dll4* (blue), *ephb2a* (red) and *ephb4a* (green) in all embryos. At 28hpf, *dll4* WT n=29 MO n=22, *ephb2* WT n=25 MO n=23, *ephb4a* WT n=27 MO=31; at 36hpf *dll4* WT n=31 MO *ephb2a* WT n=26 MO=23, *ephb4a* WT n=31 MO n=30). High expression levels are represented by solid colour, weak levels by pattern and absent expression by solid white. Zebrafish embryos shown are representative, values on the bottom right indicate number of embryos with the displayed phenotype per total number of embryos analysed, red bracket=dorsal aorta, white bracket=posterior cardinal and ventral vein(s).

Supplementary Figure 9

a Phosphorylated SMAD1/5/9 expression pattern in ECs of E9.5 embryos

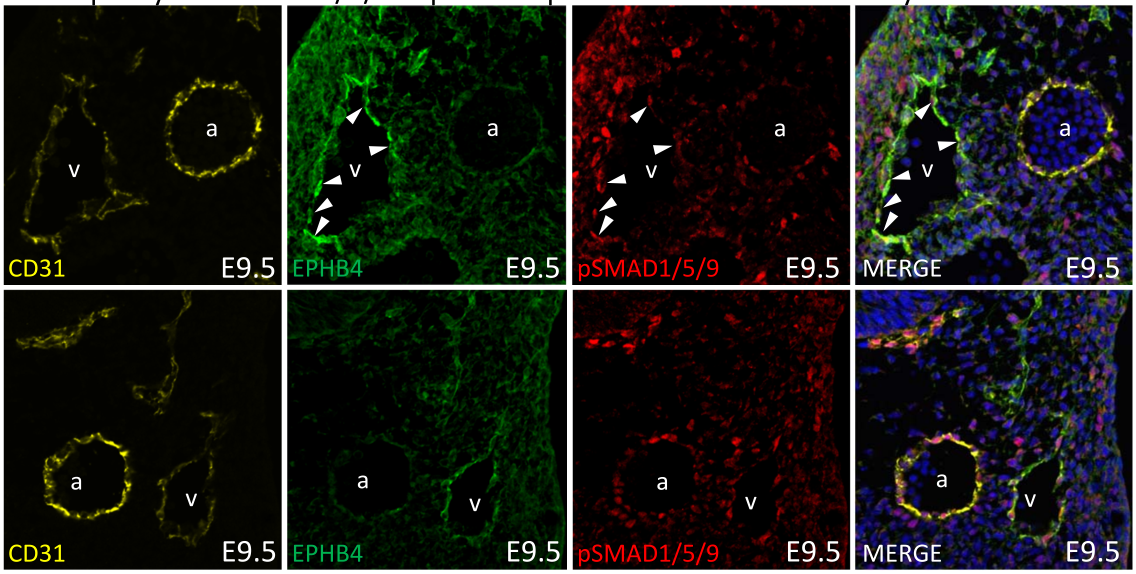

b SMAD1/5 binding peaks around known pan-endothelial

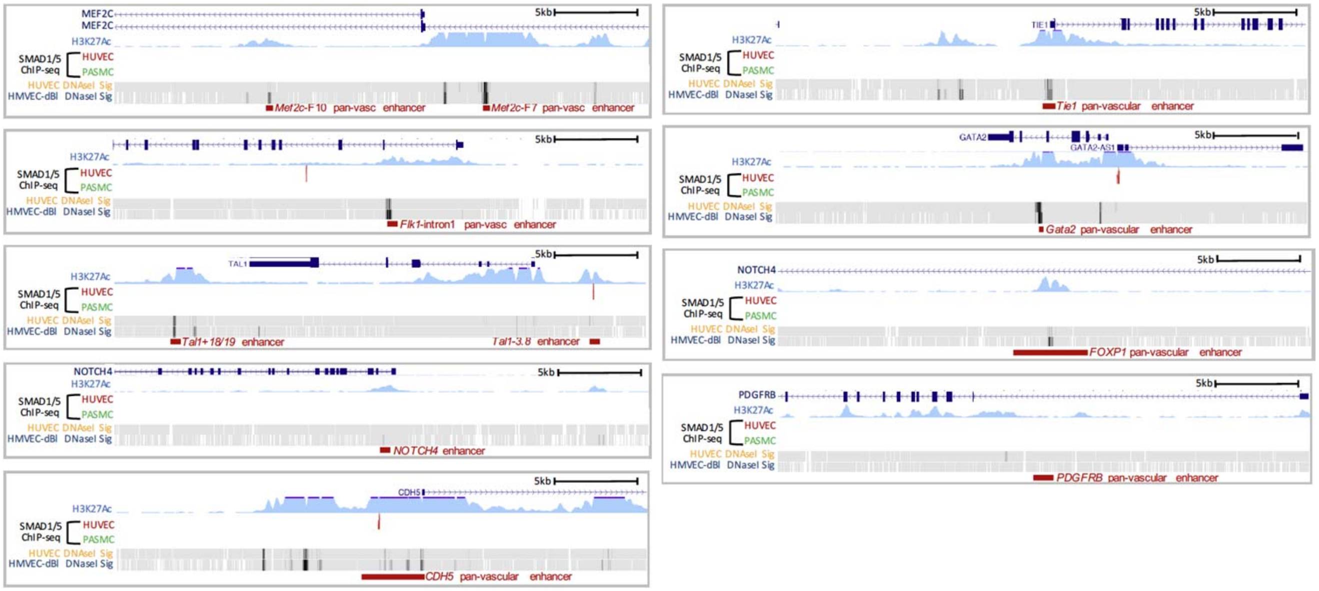

c SMAD1/5 binding peaks around known arterial enhancers

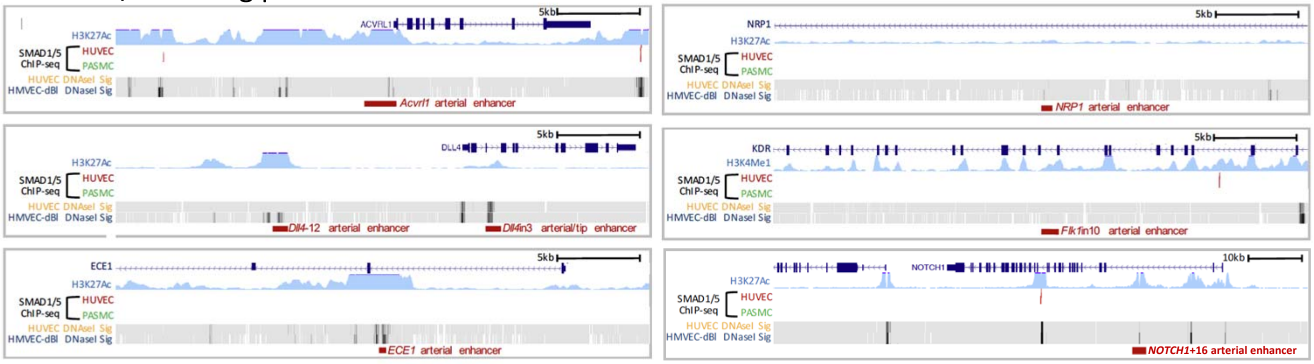

Supplementary Figure 9, relating to Figure 7 in main text.

pSMAD1/5 is found in both venous and arterial endothelium, but SMAD1/5 binding peaks are not associated with known pan-endothelial or arterial enhancers.
a. Representative transverse sections demonstrating expression of phosphorylated SMAD1/SMAD5/SMAD9 (pSMAD1/5/9) in E9.5 arterial (a) and venous (v) endothelial cells.
b-c. UCSC genome browser view of enhancer histone marks (blue, in HUVECs), SMAD1/5 binding (red and green in HUVEC and PASMC respectively) and DNase I HS (black heat map, HUVECs and HMVECs). SMAD1/5 binding was only detected over 2 of 11 known pan-vascular enhancers [12-18] (**b**). No SMAD1/5 binding was detected over known arterial specific enhancers[1,19-23](**c**).

Supplementary Figure 10

a

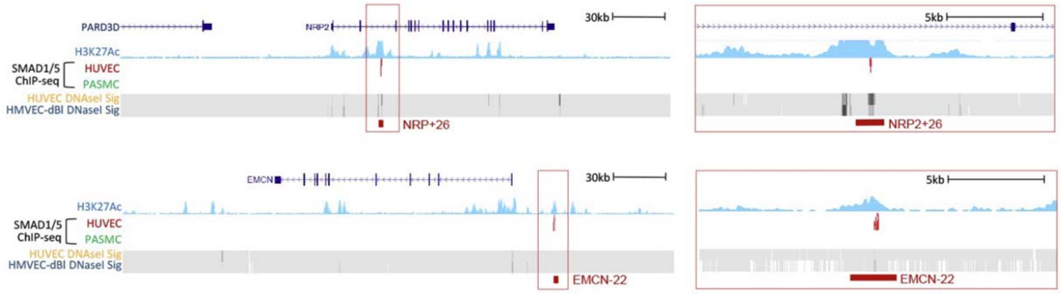

b

```
CoupTFII-965
          SBE      ETS               ETS                           ETS                     MEME1/ETS         ETS
human   GAGACAAATGGAAACTTGAAGATAAGGATCCTCTGAGGTGAGAACATACAGCTGTTGGGAATTGCTAGAGAATCTGACCAATAAAGGAAGTCACTATTTTTCCAGGCCTG
mouse   GAGACAAATGGAAAGCTGAAGATAAGGATCCTCTGAGGTGCGAACATACAGCTGTTGGGAATTGCCAGAGAATCGGACCAATAAAGGAAGTCACTATTTTTCCAGGCCTG
        ************** ************************ ************************ ******** *********************************

                          SBE                                                     MEME3                     SBE(PEAK)
human   AAGTGAGTTATAGGGCGAGACGGGTGTTGTATATTTATGTAAGGCAGCAGCAGGGAGTTTAAGCGGCTGTATATTGCTGAAAGAGCATCATTCACATTCAGGCAGAGACA
mouse   AAGTGAGTTATAGGGCGAGACGGGTGTTGTATATTTATGTAAGGCAACAGCAGGGAGTTTAAGCGGCTGGATATTGCTGAAAGAGCATCATTCACATTCAGGCGGAGACA
        ********************************************* ********************** ********************************* ******

         MEME1/ETS          ETS   MEME3                                        GC-SBE     GC-SBE    GC-SBE    MEME1/ETS
human   AAAGGTGGAAATGAAGTAACATCCTGGCTGAAGAAGGCCTCACGACAGAATAATAACATTTCAGAGAGGGGGCCGTGTGCGAGGCTGTGGGTAGGCCTCAAAATGAGGA
mouse   AAAGGTGGAAATGAAGCAACATCCTGGCCAAAGAAGGCCTCAAGACAGAATAATAACAGTTCAGAGAGGGGGGCTGTGTGCACGGCCGAGGTCGGCCTCAAAACCAGGA
        **************** ***********  **************************** *************** ******  *** * **** **********  ****

        ETS
human   AATGATCAAGATGCCTTGTCAGGCCTTCAGAAAACAGTTACACACACACACACACACACGCTCACACACACACACACACACACAGGCA
mouse   AATGATCGAGATGCCTTGTCAGATCTTCAGAAAACAGTCACACACGCACACGTGCAGGGATACACATACACACTCAAACTCA------
        ******* ************** ************** ******** *****    ** *    ***** ****** ** ** **

Nrp2+26
              SBE        SBE         SBE           MEME3     SBE          SBE
human   TTTATAGACACAGAGCAGACCAACGAAAGACCCAACACTCATGCTGAAAGCAGCCTGCCCCCAAAGCCTTAAACTTCAC-ATTGGCACCGGTTGCCTAGAAAAC------
mouse   TCTATAGTCATAAA-----------AAGAAGCTGACGGTCATGCTGAAAAGAGCCAGTCCC--AAGCCTTCAACTTCACCAGTGGCAAAAAAAAAAAAAAAAAAAAAAA
        * ***** ** * *           ** * *  **  ***********  **** * ***  ******* ******** * *****          * ****

                  ETS                    MEME3/ETS                                             SBE           GC-SBE  ETS GC-SBE
human   --------CATTCCTTACATAAGCAAACATGTTTCATCCAAGTGGCCTCCAGTGTGTGTGCTGGTCCAGAAGCAGAGCAGACCCGTGACCACACGGCCTCAGGAATGCGCC
mouse   AAAAAAAACACCCCTTACATAAGCAAGTGTTTTCTATCCAGATAGCCTCCGGAGTACCCGCTGGT-----------ACAGATCTGAGGCTCTGCGGCTTCAGGAATGTACC
                 **  **************  * **  *****  * ****** * **    ******          **** * * * *    **** *********  **

                 SBE                                                           GC-SBE
human   TTGCTGGCCAGCCAGGTCAGCGAGGGAAATGGTATTCACCCAAAGGAC----CAGTGCAGAAGTTGGGGAGGCTTTGTTGCGTGCCCTTAATTCTCTGAAAGTTCACCT
mouse   TTGCTGGGAAGCCAGGGCAGTGTGGGGAGTAGTGTGCAACTTGAGGGCAGGGCAGGGCAGGGGCACCAGTGGCTTTGTTGTGTGCCCTTCATTTTCTGAAAGTG-ACCC
        *******  ******* *** * *** * * ** * ** *    *** *    *** ****  *        ********** ******** *** *********  ***

              SBE                            MEME1/ETS                    SBE           ETS
human   TCAGAGAGACTTATCAGATGCGTTGTCTGGAGTGGCCTCCTTCCTCACGTCAGGAGGCATTCTTGGCAGCCTGCTCCATGAGGAACAATG
mouse   ACAGA-AGACTTATCAGACACTGTGTCAGGAGCAGCCTCCTTCCTCACGTAGAGAGG-GTTCTTGGCAGCCTGCGACCCAGGGAACAATG
         **** ************  *  **** ****   ****************   ****  ***************  *    *********

EMCN-22
          SBE ETS  ETS                              ETS                                  SBE     ETS                 SBE
human   AGGGCTTTCCTGGAAAAGTGACAATTACATTTCAATTT---GAAGGATA-TTCAAGAGTTAACAAGTCAAAGTAACTGGGGCTGAAAGGAAACGGGGATGACCACTCCAG
mouse   ATAAATATGCAGAACATGTCCTCTTCCCAAATCCATTTTACGAGGTGTGCTTTGAGAATGTACTTGTAAATTCAGTTTAG--TGAAAT---CTGTGTACAGACAGGGCTT
        *    * * * * * * **     *  **  ** ****  ** *  *  **  *** *  **  ** **   *  *  * *****   * * *    **  *

       SBE ETS/MEME3         SBE                 ETS/MEME3       MEME3/nGC-SBE SBE ETS                  SBE
human   ACAGGAAGAAAACCCACAAAGGTCTTGTGGCAAGAAGTAACACGGAAGGGACAAGGTACAGAAGGGAGGCCAATGTGTCTGGAAA-----GTTAGTGCAATGACAGACA-
mouse   CCTGGAAGAAA--TTACAAGCCTGTTGGAAGAAGAGTCAAATAGTCAAAAGAAGGGACCATCACTTTAGGCA----GTCTCTTAAAGGGCGTTTGTGCAGAAGGAGACAG
          * ********     ****  * ***   **** **   *  *   * **  **  * **  * ***  ****   **     *** *****      *****

                     MEME3         MEME1/ETS                      SBE                                         ETS
human   --------CACAGGGTGAGATAAGGCTGCAAAGGAAGTTGAGGAGGATGAGGTAAGGGTTT-GTGGGCTATGTTAAGAATATAAGCCTCT----ATGATAAGAGCATAGG
mouse   AGAGAGGTCACTGTGTCTGACTGTGATCCAAAGGACAGAGACGTGGGTCTTACACAGGTCCAGTGGGTGATGTGAGGGTTAACGGCTCTTTTACCTCCAGGGTGTTCA
                *** * **  **    * * *******    ** * ** *     *  ***   *****  **** * *  **   * ****    *    ** *  *

        ETS                         SBE/GC-SBE           ETS                                      SBE
human   AACAGATTTGCGTTTTGACAAGATCACCCAGTCTCCTGTATTGAGAATGGATTTGACCGAGGTAAGAGCAAATGTGG---------GAGTCTAGTAAAA----------
mouse   AATAGATGTGTGTTTTGAAAAAGATTGCCTATCACTT-TATT-ACAATGGAGATGGCAGATGCCAGAGCAAGTGTGGTCTTGTCAGAGGTCTTTCAAAGCCAGTGGTGA
        ** **** ** ******* **      **   ** * * **** * ******  ** * ** *  ******* *****          **** * ***

human   -------AAGTTACTGAAAA--------ATTCCAGGCACAAAATGATAGAAAACTTGAACTAGAGTGAAGGTAGTACATATGAAGAGAAATGAATAAATTAGGGGG-AAA
mouse   CATGCATATGCTGATGGCAAGGTTATTCATCGCAGGCACAAAACAATAGGGAACATAAACTAGAGTAGAGATAGGAAATACAAAGAGAAAGAAAGAAGGGAGTTGACAGG
               * * *  **  **       **  ***********  ****  *** * *********  ** *** * ***  ********  ** ****  *  *

           ETS                          ETS        ETS
human   CTTGGAAGGTAATATTGAGAGCACTTGGTGAAGGATTCAATTAAGGATTGAGGA
mouse   TCTAAGAGCTGACTTTGGGAGCACTTG---AAGGACTGAATTAAGAGTACGAGG
        *   ** * *  *** *********   ***** * *******    *    *
```

c

Tested enhancer	Transgenic mouse embryos screened	Any detectable *LacZ* expression	*LacZ* in vessels % *(#)*
Ephb4-2	5	5	80%(4)
Coup-TFII-965	9	9	89%(8)
EMCN-22	7	6	57%(4)
Nrp2+26	7	6	57%(4)

d

Tg(Nrp+26:LacZ) *Tg(EMCN-22:LacZ)*

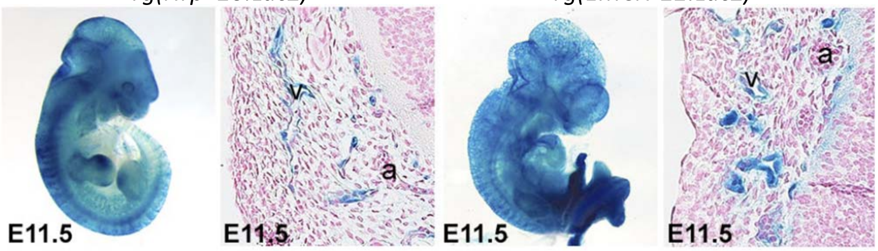

Supplementary Figure 10, relating to Figure 7 in main text.

SMAD1/5 binding identifies novel vascular enhancers for *Nrp2* and *Emcn*.
a. UCSC browser view (http://genome.ucsc.edu) of NRP2 and EMCN loci incorporating SMAD1/5 binding data from Morikawa et al[11]. Statistically significant SMAD1/5 binding peaks in HUVECs (red), HUVEC histone marks (blue) and HMVEC and HUVEC DNaseI hypersensitivity were used to identify putative enhancers.
b. ClustalW alignment of the human and mouse sequences correlating to approximately 200bp either side of significant SMAD1/5 binding peaks (red text) within the loci of *COUP-TFII* (*Nr2f2), NRP2* and *EMCN*. * denotes conserved nt between mouse and human, red text corresponds to SMAD1/5 ChIP-seq peaks from Morikawa et al. [11]. Yellow boxes denote EBEs, green boxes denote GC-SBE motifs associated with SMAD1/5 binding, blue boxes denote SBE motifs associated with all SMAD binding and grey boxes denote other over-represented motifs reported in SMAD1/5-bound endothelial cells. Sequence logos for all motifs can be seen in Supplementary Figure 4b and 6b).
c. Table summarizing the X-gal expression pattern in transient transgenic mice expressing the venous enhancers Ephb4-2:*LacZ*, CoupTFII-965:*LacZ*, EMCN-22:*LacZ* and Nrp2+26:*LacZ*. The CoupTFII-965 and Nrp2+26 enhancers were the orthologous mouse sequences to SMAD1/5 bound regions, but lack of conservation meant that the human sequence was used in the EMCN-22 enhancer.
d. X-gal expression patterns in E11.5 whole-mount and transverse sections from representative transient transgenic mice expressing the mouse Nrp2+26:*LacZ* and human EMCN-22:*LacZ* transgenes, a=artery, v=vein.

Supplementary Figure 11

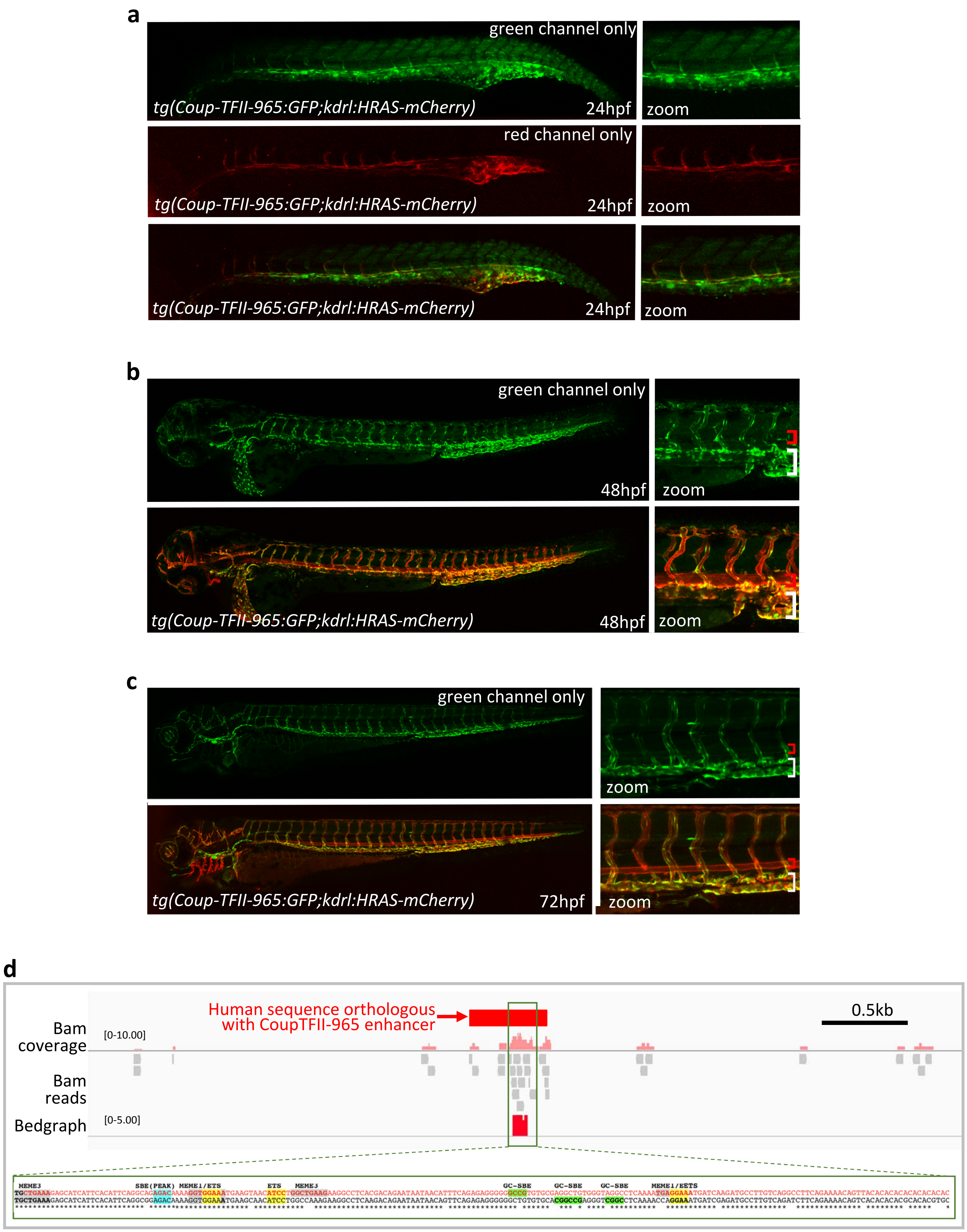

Supplementary Figure 11, relating to Figure 7 in main text.

The CoupTFII-965 enhancer is active in zebrafish
a-c Relating directly to Figure 7c, further representative fish from the stable transgenic line *tg(CoupTFII-965:GFP;kdrl:HRAS-mCherry)* at 24hpf (**a**), 48hpf (**b**) and 72 hpf (**c**, partially also shown in Fig. 7c).
d. IGV view of the human sequence orthologous to the Coup-TFII enhancer and surrounding region. Morikawa et al. [11] SMAD1/5 ChIP-seq data in HUVEC (BMP9 stimulated) is displayed as Bam coverage (pink peaks), Bam reads (grey boxes) and Bedgraph (red peaks). The exact sequence corresponding to Bam coverage peaks is shown in full, mouse sequence on top, human sequence on bottom, * denoting conserved bp . Motifs are marked: SBE (blue), GC-SBE (green) and other SMAD1/5-bound MEMEs (grey).

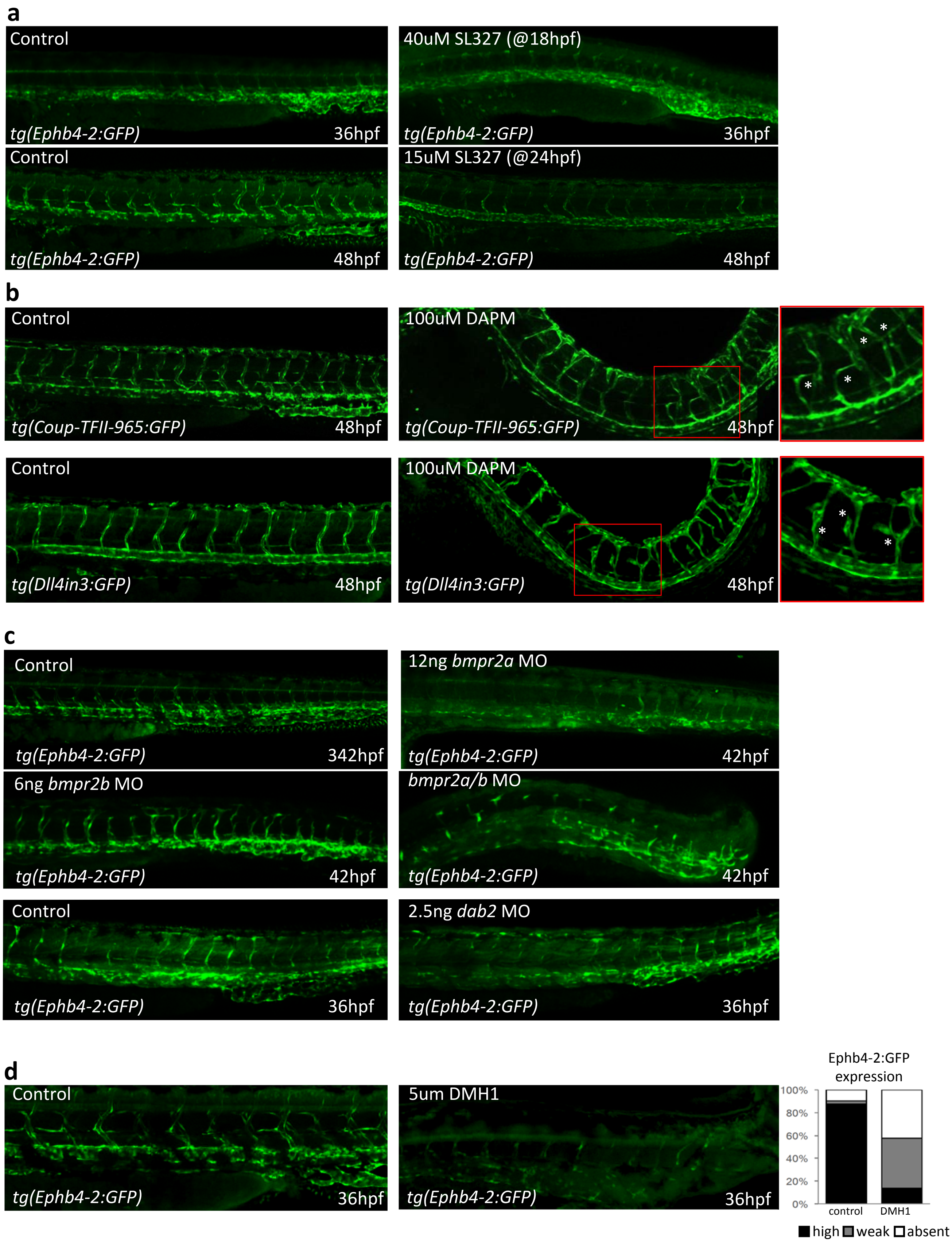
a
Control
tg(Ephb4-2:GFP)
36hpf
40uM SL327 (@18hpf)
tg(Ephb4-2:GFP)
36hpf
Control
tg(Ephb4-2:GFP)
48hpf
15uM SL327 (@24hpf)
tg(Ephb4-2:GFP)
48hpf
b
Control
tg(Coup-TFII-965:GFP)
48hpf
100uM DAPM
tg(Coup-TFII-965:GFP)
48hpf
Control
tg(Dll4in3:GFP)
48hpf
100uM DAPM
tg(Dll4in3:GFP)
48hpf
c
Control
tg(Ephb4-2:GFP)
342hpf
12ng bmpr2a MO
tg(Ephb4-2:GFP)
42hpf
6ng bmpr2b MO
tg(Ephb4-2:GFP)
42hpf
bmpr2a/b MO
tg(Ephb4-2:GFP)
42hpf
Control
tg(Ephb4-2:GFP)
36hpf
2.5ng dab2 MO
tg(Ephb4-2:GFP)
36hpf
d
Control
tg(Ephb4-2:GFP)
36hpf
5um DMH1
tg(Ephb4-2:GFP)
36hpf
Ephb4-2:GFP expression
100%
80%
60%
40%
20%
0%
control
DMH1
high
weak
absent

Supplementary Figure 12, relating to Figure 8 in main text.

Perturbation of signalling through Notch, ERK/MAPK and BMPR2 has little effect on the activity of *Ephb4-2*, while inhibition of ALK1/2/3/6 reduces Ephb4-2 activity.

a. Representative 36 and 48hpf *tg(Ephb-2:GFP)* zebrafish after DMSO alone (control) or addition of the ERK inhibitor SL327 demonstrate that Ephb4-2:GFP expression does not expand into the artery after ERK inhibition. Although high levels of SL327 inhibit arterial formation, both dorsal aorta and dorsal/ventral veins were clearly visible (using circulation) in fish after 15uM SL327 added at 24hpf. (n=40 for all groups).

b. Representative 48hpf *tg(Coup-TFII-965)* and *tg(Dll4in3:GFP)* zebrafish after DMSO alone (Coup-TFII n=51, Dll4 n=42) or 100uM DAPM treatment (Coup-TFII n=57, Dll4 n=54) demonstrate that Notch inhibition does not change Coup-TFII-965:GFP expression intensity or venous localization. As previously reported after Notch pathway inhibition, hypersprouting was clearly visible (red box denotes zoomed region), particularly in the *tg(Dll4in3:GFP)* zebrafish.

c. Representative 42hpf *tg(Ephb-2:GFP)* zebrafish without treatment (control, picture shown is from *bmpr2a* injection set) or after injection of *bmpr2a* MO (control n =30, MO n =32), *bmpr2b MO* (control =35, MO n= 25), combined *bmpr2a/b* MO (control n=40, MO n=40) and dab2 MO (control n=51, MO n=45) Ephb4-2:GFP expression was detected in all, although defects in the sprouting from the caudal vein were detected as previously described by [5,6].

d. Inhibition of BMP signalling by DMH1 severely reduces the expression of the venous Ephb4-2:GFP transgene in zebrafish. Graph depicts observed expression pattern of GFP in *tg(Ephb4-2:GFP)* embryos for DMSO-treated control (n=42) and 5µM DMH1-treated embryos (n=68), black represents high expression, grey represents weak, white represents no detected expression. Embryos shown are representative 36hpf *tg(Ephb4-2:GFP)* embryos after control and DMH1-treatment embryos.

Supplementary Figure 13

a

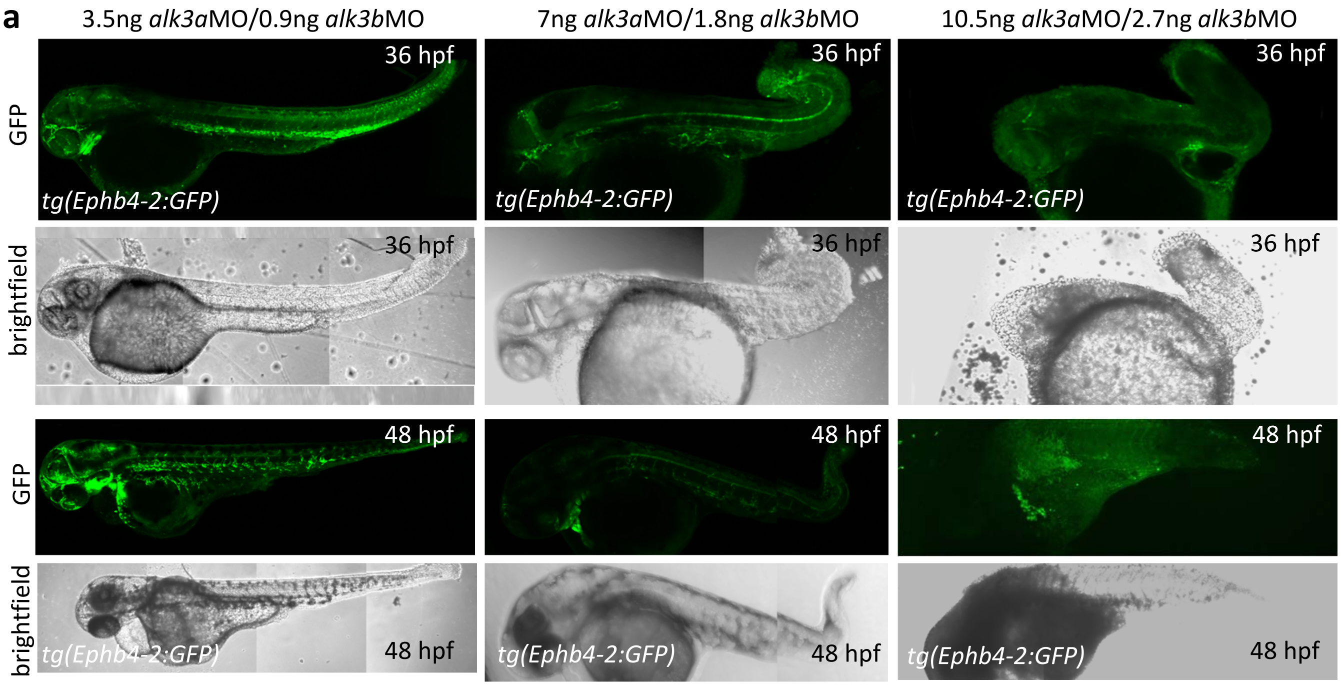

b

Ephb4-2:GFP expression at 36hpf

	% strong GFP	% low GFP	% no GFP
Low dose *alk3a/b* MO	55	41	4
Med dose *alk3a/b* MO			
High dose *alk3a/b* MO	0	22	78

Ephb4-2:GFP expression at 48hpf

	% strong GFP	% low GFP	% no GFP
Low dose *alk3a/b* MO	73	20	7
Med dose *alk3a/b* MO	2	27	71
High dose *alk3a/b* MO	0	0	100

c

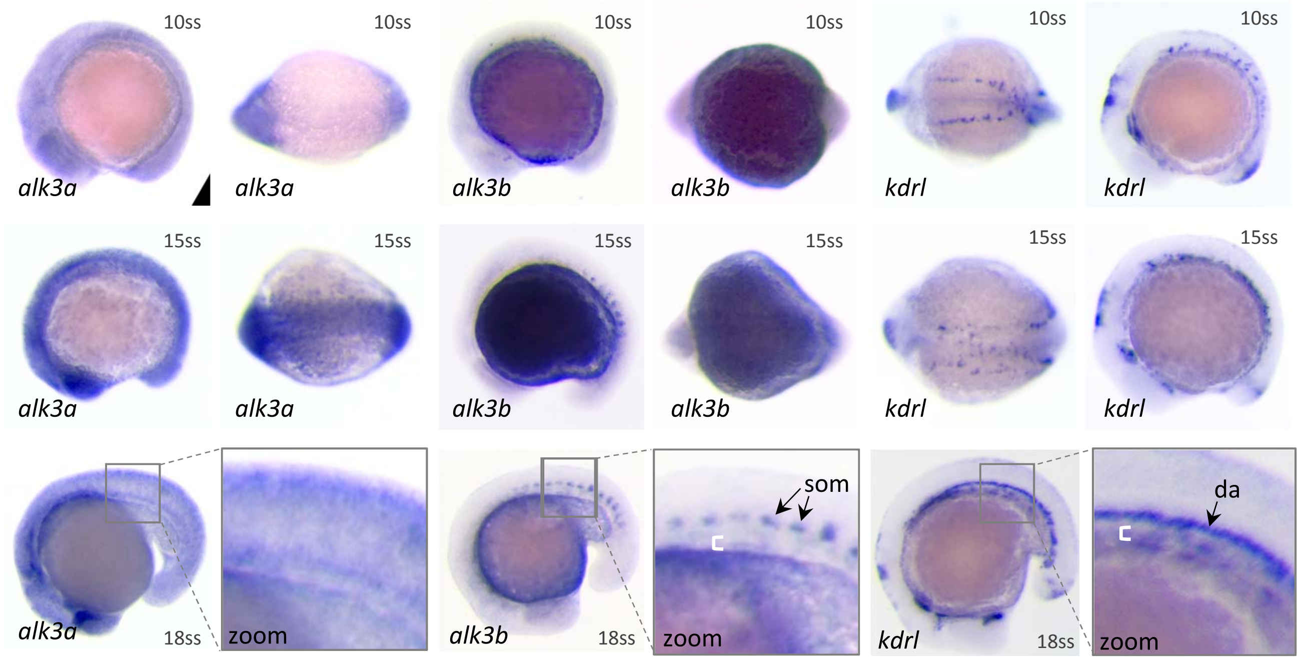

d

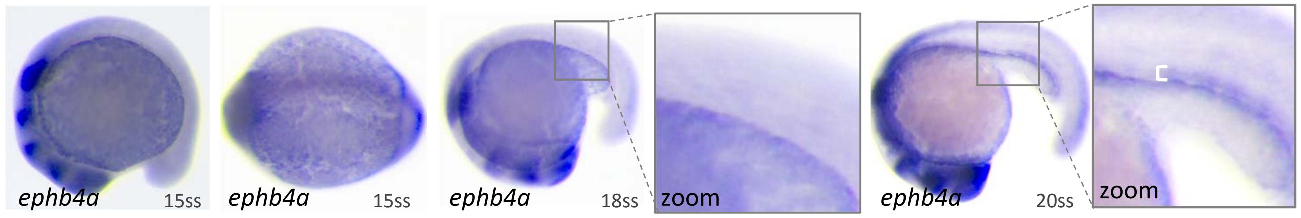

Supplementary Figure 13, relating to Figure 8in main text.

alk3a/b is required for Ephb4-2 expression and is expressed in the early zebrafish axial vein

a-b. Directly relating to Fig. 8b. Dose-response analysis of Ephb4-2:GFP expression and morphology using different concentrations of alk3a and alk3b morpholinos. Representative embryos are shown in **a** and expression patterns summarized in **b**. 3.5ng alk3aMO/0.9ng alk3bMO embryos n=54 at 36hpf, 51 at 48hpf; 14ng alk3aMO/2.7ng alk3bMO embryos n=32 at 36hpf, n=55 at 48hpf.

c. Whole-mount *in situ* hybridization for *alk3a* and *alk3b* expression compared to pan-endothelial *kdrl* in wild type zebrafish embryos at 10, 15 and 18 somite stage (ss).

d. Whole-mount *in situ* hybridization for *ephb4a* expression in wild type zebrafish embryos at 15 and 18 and 20 somite stage (ss).

Supplementary Figure 14

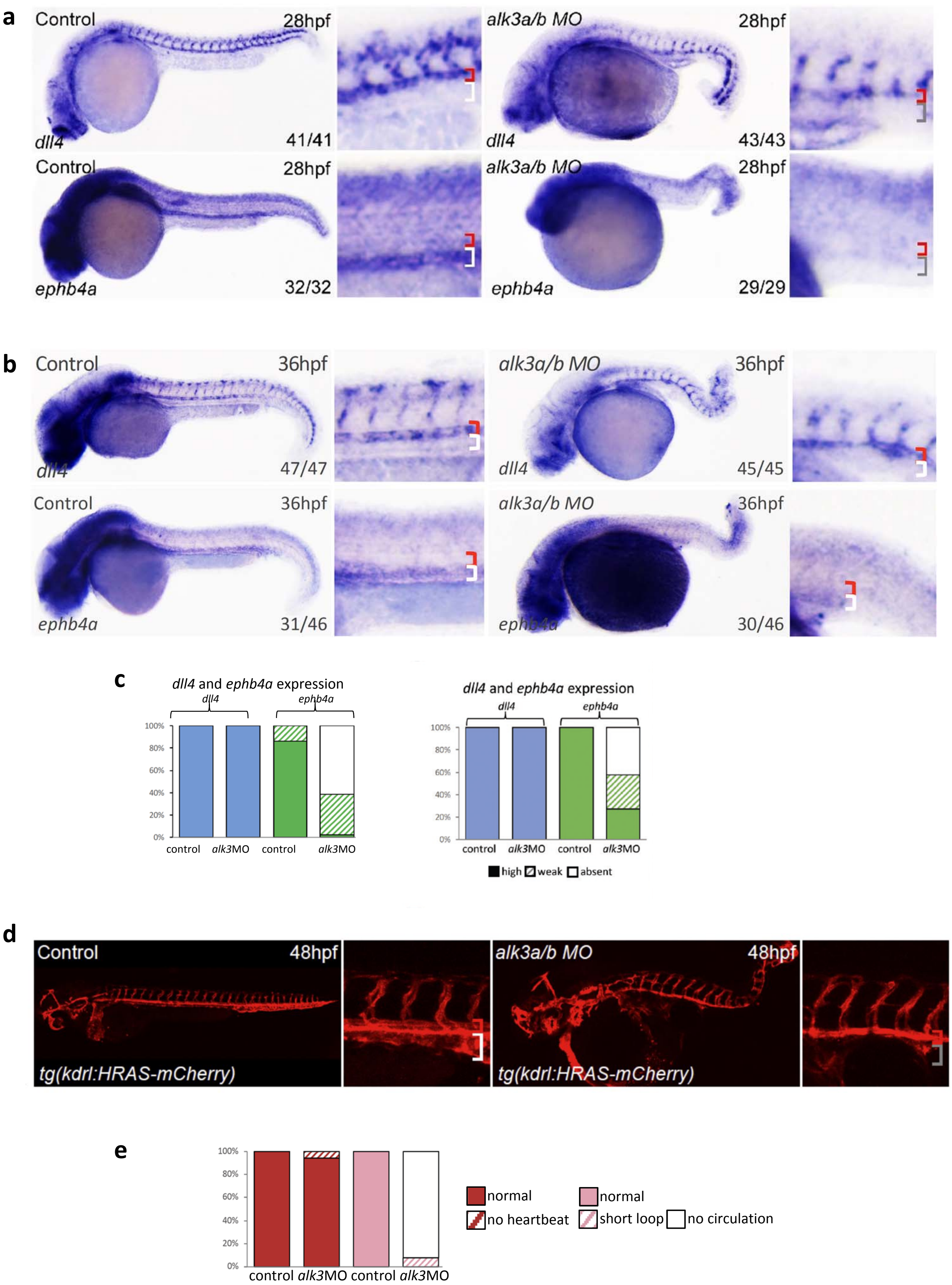

Supplementary Figure 14, relating to Figure 8 in main text.

MO-induced depletion of alk3a/b results in loss of endogenous *ephb4a* and disrupted circulation with no clear axial vessels.
a-c. Whole mount *in situ* hybridization for *dll4* and *ephb4a* in zebrafish after morpholino (MO)-mediated depletion of *alk3a/b* at 28hpf (**a**) and 36hpf (**b**). Graph (**c**) depicts observed expression of *dll4* (blue) and *ephb4a* (green) in all embryos (at 28hpf, *dll4* WT n=41 MO n=43, *ephb4a* WT n=32 MO=29; at 36 hpf, *dll4* WT n=47 MO n=45, *ephb4a* WT n=46 MO=46), high levels of expression are represented by solid colour, weak levels by pattern and absent expression by solid white. Zebrafish embryos shown are representative, values on the bottom right indicate number of embryos with the displayed phenotype per total number of embryos analysed.
d. Combined *alk3a/alk3b* knockdown resulted in the loss of a detectable axial vein at 48hpf as seen using the pan-vascular *tg(kdlr:HRAS-mCherry)* transgenic fish line.
e. Graph depicts observed status of heartbeat and circulation loop in control (n=90) and alk3a/b MO injected (n=106) fish. Normal heartbeat is represented by solid red, no heartbeat by pattern red, normal circulation is represented by solid pink, the presence of a short loop shown by pattern and no circulation by solid white. Zebrafish embryos shown are representative, red bracket=dorsal aorta, white bracket=posterior cardinal and ventral vein, grey bracket=absent vein.

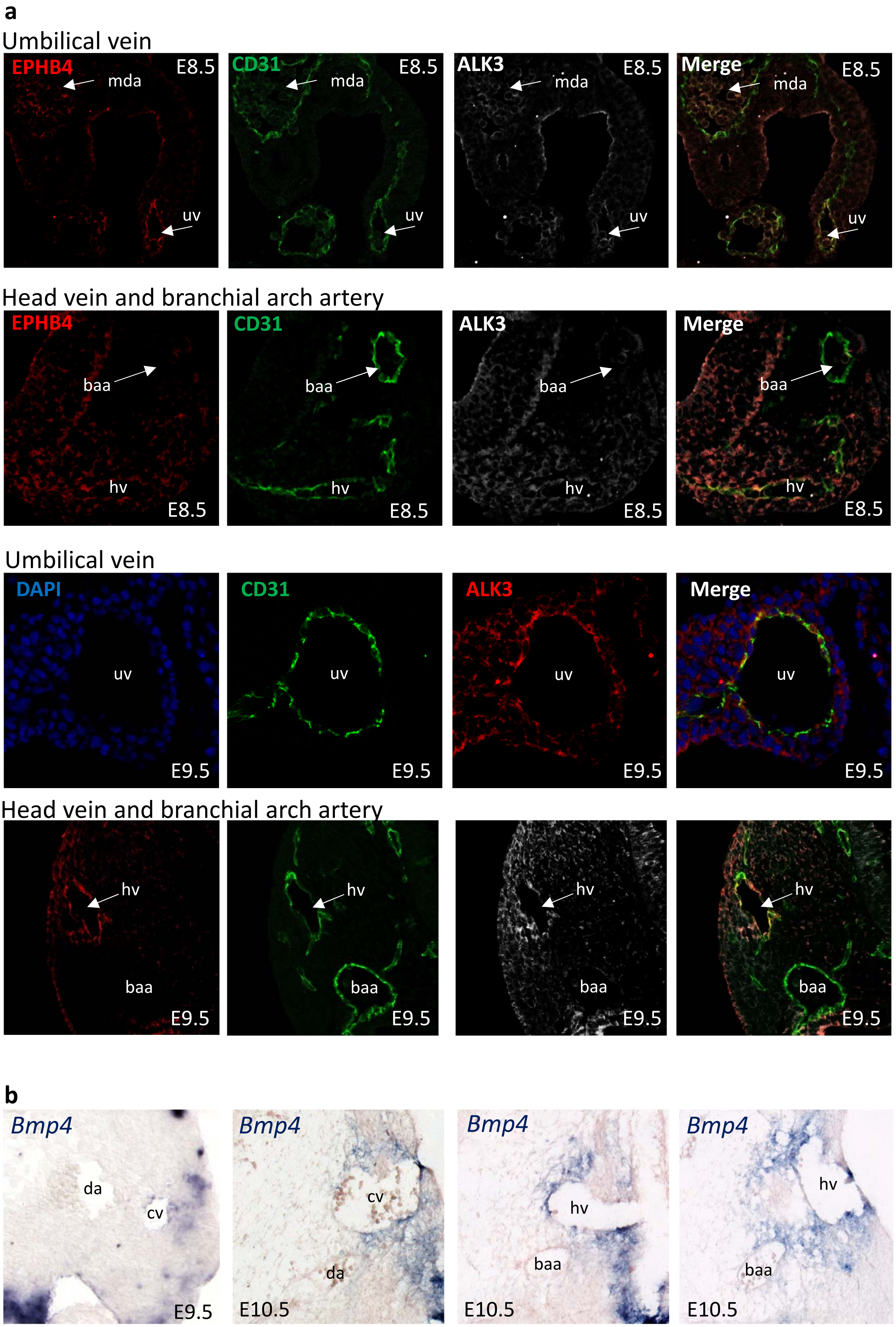
a
Umbilical vein
EPHB4
CD31
ALK3
Merge
E8.5
mda
uv
Head vein and branchial arch artery
EPHB4
CD31
ALK3
Merge
baa
hv
E8.5
Umbilical vein
DAPI
CD31
ALK3
Merge
uv
E9.5
Head vein and branchial arch artery
hv
baa
E9.5
b
Bmp4
da
cv
E9.5
E10.5
hv
baa

Supplementary Figure 15, related to Figure 9 in the main text.

ALK3 and *Bmp4* expression pattern in the early embryonic vasculature

Immunofluorescent analysis of CD31, EPHB4 and ALK3 (**a**) and *in situ* hybridization for *Bmp4* (**b**) in transverse sections from E8.5 and E9.5 mouse embryos, cv=cardinal vein, da=dorsal aorta, mda=midline dorsal aorta, uv=umbilical vein, baa=branchial arch artery, hv=head vein.

a Littermates for embryos in Figure 9

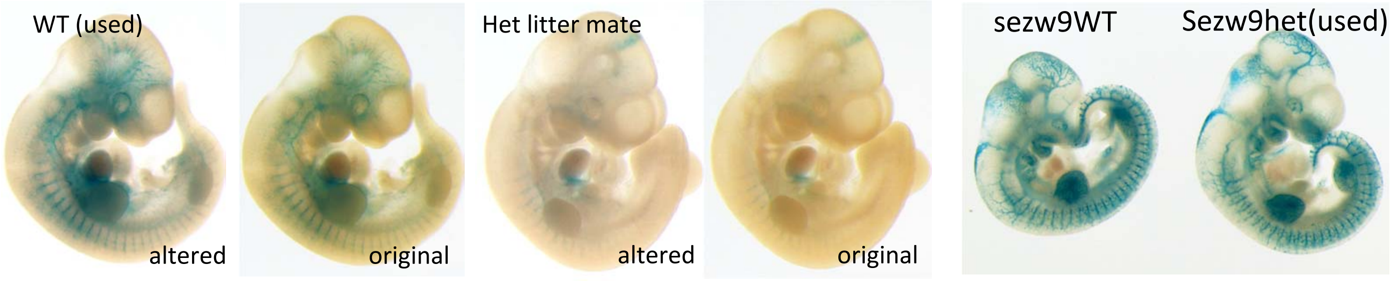

Supplementary Figure 16, relating to Figure 9 in main text.

Littermates of embryos shown in Figure 6 in cases where all genotypes were not present in a single litter. Top left and middle panel depict the same embryos after colour alteration (left) and before colour alteration (middle).

SUPPLEMENTARY REFERENCES

1. Sacilotto, N. *et al.* Analysis of Dll4 regulation reveals a combinatorial role for Sox and Notch in arterial development. *Proc. Natl. Acad. Sci USA* **110,** 11893–11898 (2013).
2. McReynolds, L. J., Gupta, S., Figueroa, M. E., Mullins, M. C. & Evans, T. Smad1 and Smad5 differentially regulate embryonic hematopoiesis. *Blood* **110,** 3881–3890 (2007).
3. Roman, B. L. *et al.* Disruption of acvrl1 increases endothelial cell number in zebrafish cranial vessels. **129,** 3009–3019 (2002).
4. Kim, J.-D. & Kim, J. Alk3/Alk3b and Smad5 mediate BMP signaling during lymphatic development in zebrafish. *Mol. Cells* **37,** 270–274 (2014).
5. Wiley, D. M. *et al.* Distinct signalling pathways regulate sprouting angiogenesis from the dorsal aorta and the axial vein. *Nat Cell Biol* **13,** 687–693 (2011).
6. Kim, J.-D. *et al.* Context-dependent proangiogenic function of bone morphogenetic protein signaling is mediated by disabled homolog 2. *Dev Cell* **23,** 441–448 (2012).
7. Sehnert, A. J. *et al.* Cardiac troponin T is essential in sarcomere assembly and cardiac contractility. *Nat Genet* **31,** 106–110 (2002).
8. Chu, G. C., Dunn, N. R., Anderson, D. C., Oxburgh, L. & Robertson, E. J. Differential requirements for Smad4 in TGFbeta-dependent patterning of the early mouse embryo. *Development* **131,** 3501–3512 (2004).
9. Park, C. *et al.* Bone morphogenetic protein receptor 1A signaling is dispensable for hematopoietic development but essential for vessel and atrioventricular endocardial cushion formation. *Development* **133,** 3473–3484 (2006).
10. Fish, J. E. *et al.* Dynamic regulation of VEGF-inducible genes by an ERK/ERG/p300 transcriptional network. *Development* **144,** 2428–2444 (2017).
11. Morikawa, M. *et al.* ChIP-seq reveals cell type-specific binding patterns of BMP-specific Smads and a novel binding motif. *Nucleic Acids Research* **39,** 8712–8727 (2011).
12. Prandini, M.-H. *et al.* The human VE-cadherin promoter is subjected to organ-specific regulation and is activated in tumour angiogenesis. *Oncogene* **24,** 2992–3001 (2005).
13. Khandekar, M. *et al.* A Gata2 intronic enhancer confers its pan-endothelia-specific regulation. *Development* **134,** 1703–1712 (2007).
14. De Val, S. *et al.* Mef2c is activated directly by Ets transcription factors through an evolutionarily conserved endothelial cell-specific enhancer. *Dev Biol* **275,** 424–434 (2004).
15. De Val, S. *et al.* Combinatorial Regulation of Endothelial Gene Expression by Ets and Forkhead Transcription Factors. *Cell* **135,** 1053–1064 (2008).
16. Kappel, A. *et al.* Identification of vascular endothelial growth factor (VEGF) receptor-2 (Flk-1) promoter/enhancer sequences sufficient for angioblast and endothelial cell-specific transcription in transgenic mice. *Blood* **93,** 4284–4292 (1999).
17. Sánchez, M. *et al.* An SCL 3' enhancer targets developing endothelium together with embryonic and adult haematopoietic progenitors. *Development* **126,** 3891–3904 (1999).

18. Göttgens, B. *et al.* The scl +18/19 stem cell enhancer is not required for hematopoiesis: identification of a 5' bifunctional hematopoietic-endothelial enhancer bound by Fli-1 and Elf-1. *Mol Cell Biol* **24,** 1870–1883 (2004).
19. Seki, T., Yun, J. & Oh, S. P. Arterial endothelium-specific activin receptor-like kinase 1 expression suggests its role in arterialization and vascular remodeling. *Circ Res* **93,** 682–689 (2003).
20. Robinson, A. S. *et al.* An arterial-specific enhancer of the human endothelin converting enzyme 1 (ECE1) gene is synergistically activated by Sox17, FoxC2, and Etv2. *Dev Biol* **395,** 379–389 (2014).
21. Becker, P. W. *et al.* An Intronic Flk1 Enhancer Directs Arterial-Specific Expression via RBPJ-Mediated Venous Repression. *Arterioscler Thromb Vasc Biol* **36,** 1209–1219 (2016).
22. Wu, J. *et al.* Molecular determinants of NOTCH4 transcription in vascular endothelium. *Mol Cell Biol* **25,** 1458–1474 (2005).
23. Chiang, I. K.-N. *et al.* SoxF factors induce Notch1 expression via direct transcriptional regulation during early arterial development. *Development* **144,** 2629–2639 (2017).
