# Supplementary material for: Venous identity requires BMP signalling through ALK3
Source: Nat Commun. 2019 Jan 28;10:453. doi: 10.1038/s41467-019-08315-w (PMC6349860; doi:10.1038/s41467-019-08315-w)
Supplement: Supplementary file 2 — Reporting Summary [file 41467_2019_8315_MOESM2_ESM.pdf]

nature research

Corresponding author(s): Sarah De Val

Reporting Summary

Nature Research wishes to improve the reproducibility of the work that we publish. This form provides structure for consistency and transparency in reporting. For further information on Nature Research policies, see Authors & Referees and the Editorial Policy Checklist.

Statistical parameters

When statistical analyses are reported, confirm that the following items are present in the relevant location (e.g. figure legend, table legend, main text, or Methods section).

n/a	Confirmed	
☐	☒	The exact sample size (n) for each experimental group/condition, given as a discrete number and unit of measurement
☐	☒	An indication of whether measurements were taken from distinct samples or whether the same sample was measured repeatedly
☐	☒	The statistical test(s) used AND whether they are one- or two-sided<br>*Only common tests should be described solely by name; describe more complex techniques in the Methods section.*
☒	☐	A description of all covariates tested
☒	☐	A description of any assumptions or corrections, such as tests of normality and adjustment for multiple comparisons
☐	☒	A full description of the statistics including central tendency (e.g. means) or other basic estimates (e.g. regression coefficient) AND variation (e.g. standard deviation) or associated estimates of uncertainty (e.g. confidence intervals)
☒	☐	For null hypothesis testing, the test statistic (e.g. F, t, r) with confidence intervals, effect sizes, degrees of freedom and P value noted<br>*Give P values as exact values whenever suitable.*
☒	☐	For Bayesian analysis, information on the choice of priors and Markov chain Monte Carlo settings
☒	☐	For hierarchical and complex designs, identification of the appropriate level for tests and full reporting of outcomes
☒	☐	Estimates of effect sizes (e.g. Cohen's d, Pearson's r), indicating how they were calculated
☐	☒	Clearly defined error bars<br>*State explicitly what error bars represent (e.g. SD, SE, CI)*

Our web collection on statistics for biologists may be useful.

Software and code

Policy information about availability of computer code

Data collection

Microscope images were analysed with ZEN, ImageJ and Adobe photoshop.

Data analysis

Statistics were analysed with Excel, graphs were generated with Excel and R (R Foundation for Statistical Computing, Vienna, Austria. URL https://www.R-project.org/).

For ChIP-Seq analysis SMAD1/5 binding information was obtained using publically available ChIP-seq data 31 via the NCBI GEO database 76, accession number GSE27661. Raw reads for HUVEC stimulated by BMP9 and PASMC stimulated by BMP4 sets were trimmed with Sickle. Reads were then aligned to human genome build hg19 using Bowtie2, duplicate PCR reads removed with rmdup, and peaks were called with MACS2. A bedgraph was generated of the significant peaks for visualization and comparison to other genomic datasets.

ERG binding information from HUVEC was obtained using publically available ChIP-seq data 29 via ArrayExpress73, accession number E-MTAB-5148. Raw reads were trimmed with Sickle, aligned to human genome build hg19 using Bowtie2, duplicate PCR reads removed with rmdup, and peaks were called with MACS2. A bedgraph was generated of the significant peaks for visualization and comparison to other genomic datasets.

For manuscripts utilizing custom algorithms or software that are central to the research but not yet described in published literature, software must be made available to editors/reviewers upon request. We strongly encourage code deposition in a community repository (e.g. GitHub). See the Nature Research guidelines for submitting code & software for further information.

Data

Policy information about availability of data

All manuscripts must include a data availability statement. This statement should provide the following information, where applicable:

- Accession codes, unique identifiers, or web links for publicly available datasets
- A list of figures that have associated raw data
- A description of any restrictions on data availability

The authors declare that the main data supporting the findings of this study are available within the article, its Supplementary Figures and Methods. All ChIP-seq datasets used in this study were previously published and are publicly available, references and accession numbers are provided within the article. Extra data are available from the corresponding author upon request.

Field-specific reporting

Please select the best fit for your research. If you are not sure, read the appropriate sections before making your selection.

☒ Life sciences ☐ Behavioural & social sciences ☐ Ecological, evolutionary & environmental sciences

For a reference copy of the document with all sections, see nature.com/authors/policies/ReportingSummary-flat.pdf

Life sciences study design

All studies must disclose on these points even when the disclosure is negative.

Sample size	Mouse: Numbers of transgenic mice used followed precedent set by similar published papers. Where significant variations in expression were detected within a single experimental set this is represented in the relevant figure by representative pictures of each outcome in combination with the n number for each example. Zebrafish: Numbers of transgenic zebrafish embryos used followed precedent set by similar published papers, and was no less than 40/transgene. Where variations in expression were detected within a single experimental set this is represented in the relevant figure by a graph summarizing variance with n numbers provided in legend. Morpholino and chemical inhibition: All MO injections and use of chemical inhibitors were conducted at least three separate times. Numbers of zebrafish embryos was no less than 40/ tg(transgene) line. Where variations in expression were detected within a single experimental set this is represented in the relevant figure by a graph summarizing variance with n numbers provided in legend. In situ hybridization: All in situ analyses were conducted at least two separate times. Analysis was qualitative not quantitative, therefore no statistical analysis was applied to the observations of staining intensity and pattern. Numbers of zebrafish embryos was no less than 20/in situ/condition. Where variations in expression were detected within a single experimental set this is represented in the relevant figure by a graph summarizing variance with n numbers provided in legend, and by the numbers next to the representative pictures. No experimental randomization was used as we did not consider this necessary.
Data exclusions	For Smad4EC/EC crosses with Ephb4Lacz/+ and Ephb4-2:LacZ, in the rare cases where the LacZ genotyping results did not agree with the pattern of X-gal staining in WT embryos, error was presumed and the entire litter was excluded.
Replication	All experimental findings were reliably reproduced at least 3 times unless otherwise clearly stated in the manuscript
Randomization	There was no randomization used
Blinding	No experimental blinding was used as phenotypes of control and treated embryos were easily detectable.

Reporting for specific materials, systems and methods

Materials & experimental systems

n/a	Involved in the study	
☒	☐	Unique biological materials
☐	☒	Antibodies
☒	☐	Eukaryotic cell lines
☒	☐	Palaeontology
☐	☒	Animals and other organisms
☒	☐	Human research participants

Methods

n/a	Involved in the study	
☒	☐	ChIP-seq
☒	☐	Flow cytometry
☒	☐	MRI-based neuroimaging

Antibodies

Antibodies used	All antibodies used are commercially available. Immunostaining: goat polyclonal to DLL4 (R&D systems, AF1389) rat monoclonal to CD31 (DIA-310, Dianova), and rabbit monoclonal to Neuropilin 1 (ab81321, Abcam), rabbit polyclonal to ALK3 (ab38560, Abcam), goat polyclonal to EPHB4 (AF446, R&D Systems), COUP-TFII (PP-H7147-00, Perseus Proteomics Inc) . Species-specific Alexa Fluor® or HRP-conjugated secondary antibodies were purchased from Thermo Fisher Scientific. For Chromatin immunoprecipitation analysis Smad1 (Iwai North America BMR00479), or IgG control (12-371 merckmillipore) .
Validation	All antibodies used are commercially available and are validated for the species and technique for which they were used. Validation data is easily accessible for all antibodies on the website of the manufacturing company by searching the product numbers that are all provided above.

Animals and other organisms

Policy information about studies involving animals; ARRIVE guidelines recommended for reporting animal research

Laboratory animals	Mouse, mus musculus: embryonic day 8.5 to post natal day 5 were used in analysis, sex unknown, and are a mixed strain of C57BL6 and and . Zebrafish, Danio rerio embryos were all used between 0 and 72 hours post fertilization
Wild animals	N/A
Field-collected samples	N/A
